# Detection of bile acids in bronchoalveolar lavage fluid defines the inflammatory and microbial landscape of the lower airways in infants with cystic fibrosis

**DOI:** 10.1186/s40168-023-01543-9

**Published:** 2023-06-13

**Authors:** Jose A. Caparrós-Martín, Montserrat Saladie, S. Patricia Agudelo-Romero, F. Jerry Reen, Robert S. Ware, Peter D. Sly, Stephen M. Stick, Fergal O’Gara, on behalf of the COMBAT study group

**Affiliations:** 1 Wal-Yan Respiratory Research Centre, Telethon Kids Institute, Perth, WA Australia; 2 Curtin Health Innovation Research Institute (CHIRI), Curtin University, Perth, WA Australia; 3 Present Address: Eurecat, Centre Tecnològic de Catalunya, Centre for Omic Sciences (COS), Joint Unit Universitat Rovira I Virgili-EURECAT, Reus, Spain; 4 The University of Western Australia, Perth, WA Australia; 5 School of Microbiology, University College Cork, Cork, Ireland; 6 Synthesis and Solid State Pharmaceutical Centre, University College Cork, Cork, Ireland; 7 Menzies Health Institute Queensland, Griffith University, Brisbane, Australia; 8 Children’s Health and Environment Program, Child Health Research Centre, The University of Queensland, Brisbane, Australia; 9 Department of Respiratory Medicine, Princess Margaret Hospital for Children, Perth, WA Australia; 10 BIOMERIT Research Centre, School of Microbiology, University College Cork, Cork, T12 K8AF Ireland

**Keywords:** Cystic fibrosis, Bile acids, Lung microbiota, Inflammation, Neutrophils, Gut-lung axis, Azithromycin

## Abstract

**Background:**

Cystic Fibrosis (CF) is a genetic condition characterized by neutrophilic inflammation and recurrent infection of the airways. How these processes are initiated and perpetuated in CF remains largely unknown. We have demonstrated a link between the intestinal microbiota-related metabolites bile acids (BA) and inflammation in the bronchoalveolar lavage fluid (BALF) from children with stable CF lung disease. To establish if BA indicate early pathological processes in CF lung disease, we combined targeted mass spectrometry and amplicon sequencing-based microbial characterization of 121 BALF specimens collected from 12-month old infants with CF enrolled in the COMBAT-CF study, a multicentre randomized placebo-controlled clinical trial comparing azithromycin versus placebo. We evaluated whether detection of BA in BALF is associated with the establishment of the inflammatory and microbial landscape of early CF lung disease, and whether azithromycin, a motilin agonist that has been demonstrated to reduce aspiration of gastric contents, alters the odds of detecting BA in BALF. We also explored how different prophylactic antibiotics regimens impact the early life BALF microbiota.

**Results:**

Detection of BA in BALF was strongly associated with biomarkers of airway inflammation, more exacerbation episodes during the first year of life, increased use of oral antibiotics with prolonged treatment periods, a higher degree of structural lung damage, and distinct microbial profiles. Treatment with azithromycin, a motilin agonist, which has been reported to reduce aspiration of gastric contents, did not reduce the odds of detecting BA in BALF. Culture and molecular methods showed that azithromycin does not alter bacterial load or diversity in BALF. Conversely, penicillin-type prophylaxis reduced the odds of detecting BAs in BALF, which was associated with elevated levels of circulating biomarkers of cholestasis. We also observed that environmental factors such as penicillin-type prophylaxis or BAs detection were linked to distinct early microbial communities of the CF airways, which were associated with different inflammatory landscapes but not with structural lung damage.

**Conclusions:**

Detection of BA in BALF portend early pathological events in CF lung disease. Benefits early in life associated with azithromycin are not linked to its antimicrobial properties.

Video Abstract

**Supplementary Information:**

The online version contains supplementary material available at 10.1186/s40168-023-01543-9.

## Background

Cystic fibrosis (CF, OMIM 219700) is a genetic disorder caused by mutations in the *Cystic Fibrosis Transmembrane Conductance Regulator* (*CFTR*) gene, which consequently alters ion homeostasis [1]. In CF, respiratory complications remain the primary contributor to morbidity and mortality [1]. CF lung disease progresses early after birth, even in asymptomatic children [2]. Neutrophilic inflammation and recurrent respiratory infections lead to the scarring of the lung epithelia, which influences clinical outcomes of the disease [3]. Consequently, early interventions are required to preserve the lung epithelia until CF patients initiate CFTR restorative therapies.

In CF, genetic and environmental factors determine a distinct lung microenvironment characterized by mucus plugging [4] and hypoxia [5]. This niche shapes the airway inflammatory landscape by promoting both an abnormal microbiota [6], and the functional reprogramming of infiltrated neutrophils [7]. As patients age the CF lung microbiota transitions from highly diverse to pathogen-dominated communities [6, 8]. This shift is associated with increased inflammation and reduced lung function [8]. While microbial diversity could be a determinant of disease progression, this may be confounded by the intensive antibiotic therapies that CF patients receive. Airway colonisation by pathogens has undeniable structural consequences on the airway epithelia, however inflammation in the absence of microbial or viral stimuli is also observed in bronchoalveolar lavage fluid (BALF) from infants with CF [9]. Furthermore, the cumulative effect of neutrophilic inflammation on structural lung damage in children with CF is higher than microbial infection [10], suggesting that identifying and targeting early inflammatory triggers could translate into long-term clinical benefits for CF patients.

Current clinical practice does not prevent permanent damage to the bronchial tree, which is evident in most school-aged children with CF [2]. Thus, a major clinical problem remains in the identification of the processes involved in igniting and maintaining the inflammatory milieu in CF lungs in early life, which eventually lead to bronchiectasis [2]. Several pathobiological factors such as neutrophil reprogramming and the accumulation of insoluble mucus flakes in the lungs have been linked to inflammation and airway remodelling in early life [4, 7, 11]. We have shown that the presence of bile acids (BA) in the BALF of pre-school aged children with CF is associated with early airway inflammation, and is a predictor of structural lung disease progression in both cross-sectional and longitudinal cohorts [12, 13]. Bile acids are intestinal metabolites closely linked to the activity of the gut microbiota [14], which may participate in the progression of CF lung disease by modulating the transmission pathway of the gut-lung axis [15].

To establish if BA indicate early pathological processes in CF lung disease, this study was conducted to profile the inflammatory and microbial landscapes in BALF related to detection of BA. Bronchial wash specimens were collected from 12-month old infants with CF enrolled in the COMBAT-CF study, a placebo-controlled clinical trial with azithromycin [16]. Considering the well-known prokinetic properties of azithromycin [17], we evaluated whether treatment with macrolides affected the odds of detecting BA in BALF. Given the young age of this patient cohort, we explored how different prophylactic antibiotic regimens impacted the BALF microbiota early in life.

## Results

### Study cohort

BALF specimens (*n* = 121) were collected from infants (12-month old) with CF enrolled in the COMBAT-CF study. COMBAT-CF was a multicentre randomized placebo-controlled double-blinded study on the efficacy of azithromycin to reduce extent of bronchiectasis at age 3-years (Clinicaltrials.gov: NCT01270074) [16]. Of the eight participating centres, five routinely used anti-*Staphylococcus* prophylaxis with either flucloxacillin or amoxicillin/clavulanic acid from diagnosis until at least 1 year of age (1 Centre), with most Centres prescribing these until the age of two. Of the 121 patients included in this study (Table S1), four reported short-term macrolide treatment during the first year of life (Table S2). None of these events occurred at the time of bronchoscopy.

### Detection of bile acids in BALF correlates with inflammation and clinical outcomes

**Fig. 1:**
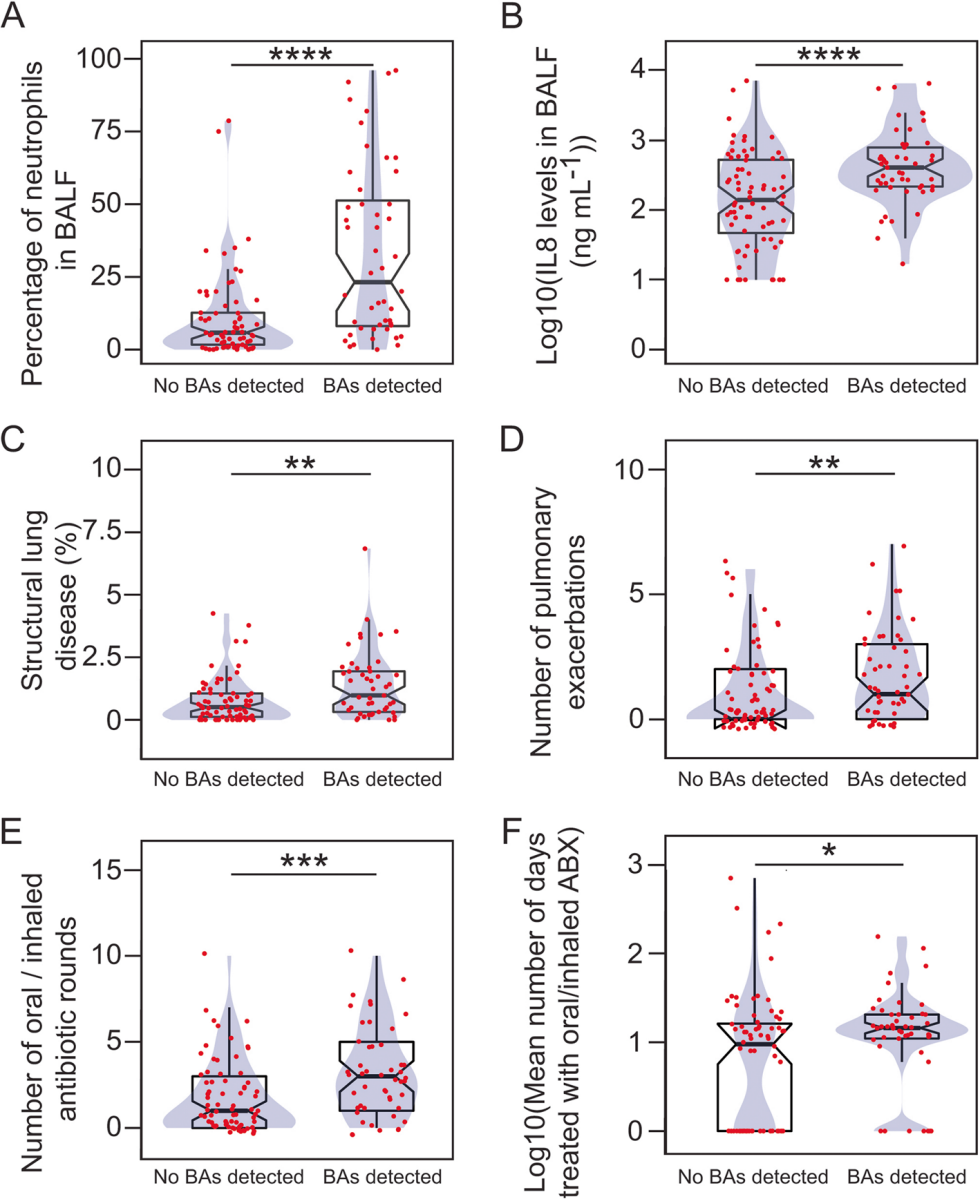
Detection of bile acids in BALF associates with airway inflammation and clinical outcomes. **A-H** Box plots overlaid with density curves (violin plots, blue) representing the proportion of neutrophils (**A**) and the levels of IL8 (**B**), the proportion of the lungs with structural disease (**C**), the number of pulmonary exacerbations (**D**), the number of oral/inhaled antibiotic rounds (**E**), the average number of days (Log10 transformed) treated with oral/inhaled antibiotics (**F**), with respect to the detection of BAs in BALF. Data in **C** was obtained from CT scan images evaluated with the PRAGMA-CF scoring system [18]. In **D-F**, the number of events during the first year of life is represented. The data represented in **F** includes the total duration of any treatment started before the collection of the BALF sample. Individual data points (red) with jitter are depicted on the top of each box plot. Groups were compared using the Wilcoxon rank-sum test: *, *p* < 0.05; **, *p* < 0.01; ***, *p* < 0.001; ****, *p* < 0.0001; n.s., *p* > 0.05

We detected bile acids (BA) in 49/121 (40%) of the BALF specimens, with a concentration range of 0.003–1.095 µM (median 0.019 µM, IQR = 0.011–0.078 µM) (Table S3). Identification of BA was associated with a higher proportion of infiltrated neutrophils (BA detected: median [IQR] 23.16 [8.08–51.25]; no BA detected: 5.80 [1.67–12.67]; Wilcoxon rank-sum test (WRST) (effect size (r) = 0.43, *p* = 0.000003)), and elevated levels of IL8 (pg mL^−1^, BA detected: 405.79 [216.59–786.20]; no BA detected: 139.52 [47.11–524.73]; WRST (*r* = 0.34, *p* = 0.0002)) (Fig. 1A-B and Fig. S1). Elevated neutrophil elastase (NE) activity was observed in fewer BALF samples (6/65, 9%) (median [IQR], 0.81 [0.40–1.80] ng mL^−1^), which also contained BA (Fig. S2). There was an association between NE and BA in BALF (*p* = 0.003, Fisher’s exact test (FET)).

Patients with BA detected in BALF demonstrated higher percentage of structural lung disease (median [IQR], BA detected: 0.97 [0.32–1.94]; no BA detected: 0.51 [0.12–1.05]; WRST (*r* = 0.27, *p* = 0.003)) (Fig. 1C and Fig. S3). Identification of BA in BALF was also associated with more exacerbations during the first year of life (median [IQR], BA detected: 1 [0–3]; no BA detected: 0 [0–2]; WRST (*r* = 0.27, *p* = 0.003)), and more courses of oral/inhaled antibiotics (median [IQR], BA detected, 3 [1-5]; no BA detected, 1 [0–3]; WRST (*r* = 0.31, *p* = 0.0008)), with longer treatment periods (days treated, median [IQR], BA detected: 13.4 [10–19.5]; no BA detected: 8.5 [0–15.2]; WRST (*r* = 0.22, *p* = 0.02)) (Fig. 1D-F and Fig. S3). We did not observe differences in the length of hospital stay in the case of exacerbations, or the number of courses of intravenous antibiotics received during the first year of life (Fig. S3-4).

### The microbial landscape of the lower airways in infants with CF

We profiled BALF-associated bacterial and fungal communities using amplicon-sequencing strategies. To monitor DNA contamination by laboratory reagents and clinical instrumentation, we processed and sequenced negative extraction controls and bronchoscope washes alongside the BALF specimens (see Methods section for a comprehensive description). We observed a good correspondence between microorganisms detected by culture and molecular methods (Table S4).

Mean concentrations of microbial DNA were similar between instrument and negative extraction controls, and lower than BALF specimens (Fig. S5 and Table S5). The most abundant operational taxonomic units (OTUs) observed in BALF were assigned to the *Stenotrophomonas* (mean (standard deviation), 36% (36)) and *Streptococcus* (22% (18)) taxa (Fig. S6-7). Alpha diversity was positively associated with bacterial burden (Fig. S8A). This relationship was driven by the proportions of oral commensals (Fig. S8C). Pathogen density had negligible effect on microbial diversity, except for a low degree correlation between Shannon diversity and *Stenotrophomonas maltophilia* load (Fig. S8B,D). Likewise, inflammatory markers were either not correlated or weakly associated with bacterial load and diversity (Fig. S9). Bacterial diversity but not bacterial burden, correlated with the number of exacerbations during the first year of life (1.37 times more exacerbations per unit increase in diversity, 95% Confidence interval, 1.12–1.70, *p* = 0.003) (Fig. S10A-B). Bacterial load or diversity in BALF were not associated with the proportion of structural lung disease at 12 month of age (Fig. S10C-D).

We obtained ITS2 library amplification from 37/121 (31%) of the BALF DNA extracts. The fungal profiles were dominated by taxa from the Phyla *Basidiomycota* (mean (standard deviation), 54% (22)) and *Ascomycota* (44% (20)) (Fig. S11). ITS2 reads were mostly assigned to amplicon sequence variants (ASVs) representing saprophytic yeasts associated with healthy oronasal cavity, human skin, breast milk, or sputum from CF patients (Table S6 and Fig. S11) [19–24]﻿. *Aspergillus* and *Candida* species were observed at markedly lower abundance and prevalence (Table S6 and Fig. S11). We confirmed the presence of genomic material from the commensal *Malassezia restricta* through qPCR; being at a higher concentration in those DNA extracts yielding ITS2 library amplification (Fig. S12A-B). *Malassezia restricta* load was not associated with antibiotic prophylaxis at the time of bronchoscopy (Fig. S12C-D). However, we observed a reduction in fungal alpha diversity in BALF from patients treated with beta-lactam and not with macrolide antibiotics (Fig. S13).

**Fig. 2:**
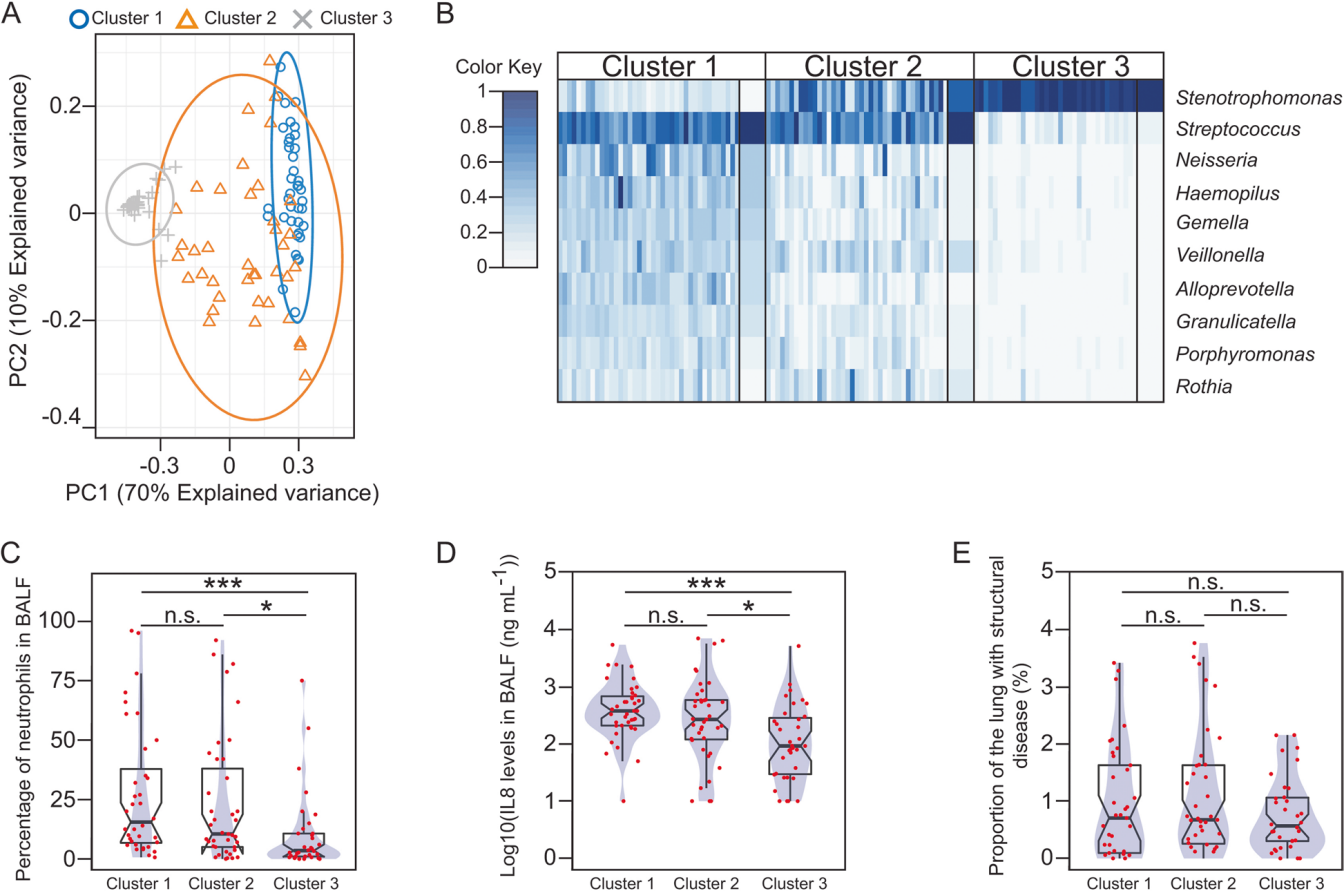
Early microbial assemblies associate with contrasting inflammatory outcomes. **A** Principal component analysis shows the linear projection of the 16S-based compositional profiles onto the first two components of the model. Each sample is labelled based on the DMM model-based cluster membership. **B** The heatmap summarises the 16S taxonomic profiles grouped by Dirichlet component (metacommunity/cluster) (cluster 1, *n* = 39; cluster 2, *n* = 39; cluster 3, *n* = 35). Rows represent OTUs, which are ordered from top to bottom based on their contribution to each metacommunity. For simplification, only the top 10 OTUs are represented. The intensity of the colour in the heatmap is proportional to the square-root of the relative abundance of each OTU. For each Dirichlet component, narrow columns represent BALF samples, and wide columns represent the component mean abundance of each OTU. **C-E** Box plots overlaid with density curves (violin plots, blue) representing the proportion of neutrophils (**C**) and the levels of IL8 (**D**) in BALF, and the proportion of the lung with structural disease (**E**), with respect to the different metacommunities. Groups were compared using the Wilcoxon rank-sum test and p-values corrected using the Bonferroni method: *, *p* < 0.05; ***, *p* < 0.001; n.s., *p* > 0.05

To evaluate whether early bacterial communities are related to different clinical outcomes, we performed a community-based clustering using Dirichlet Multinomial Mixtures [25]. Three community types were determined from the 16S rRNA profiles (Fig. 2A-B and Fig. S14). We found *Stenotrophomonas* and *Streptococcus* to be the most important drivers defining each ecotype (Fig. S15). Bacterial and *Stenotrophomonas maltophilia* density, contributed to differences between metacommunities (Fig. S16). Community type three demonstrated a significant reduction in clinical markers of inflammation including IL8 and the proportion of neutrophils in BALF (Fig. 2C-D). None of the microbial ecotypes were associated with a higher degree of lung structural damage (Fig. 2E). Interestingly, different metacommunities were linked to detection of BA in BALF (*p* = 0.000004, FET), and to penicillin-type antibiotic prophylaxis at the time of BALF collection (*p* = 0.000000002, FET), but not to azithromycin therapy (*p* = 0.9, FET) (Fig. S17A-C).

### Detection of BA in BALF correlates with distinctive microbial profiles

**Fig. 3:**
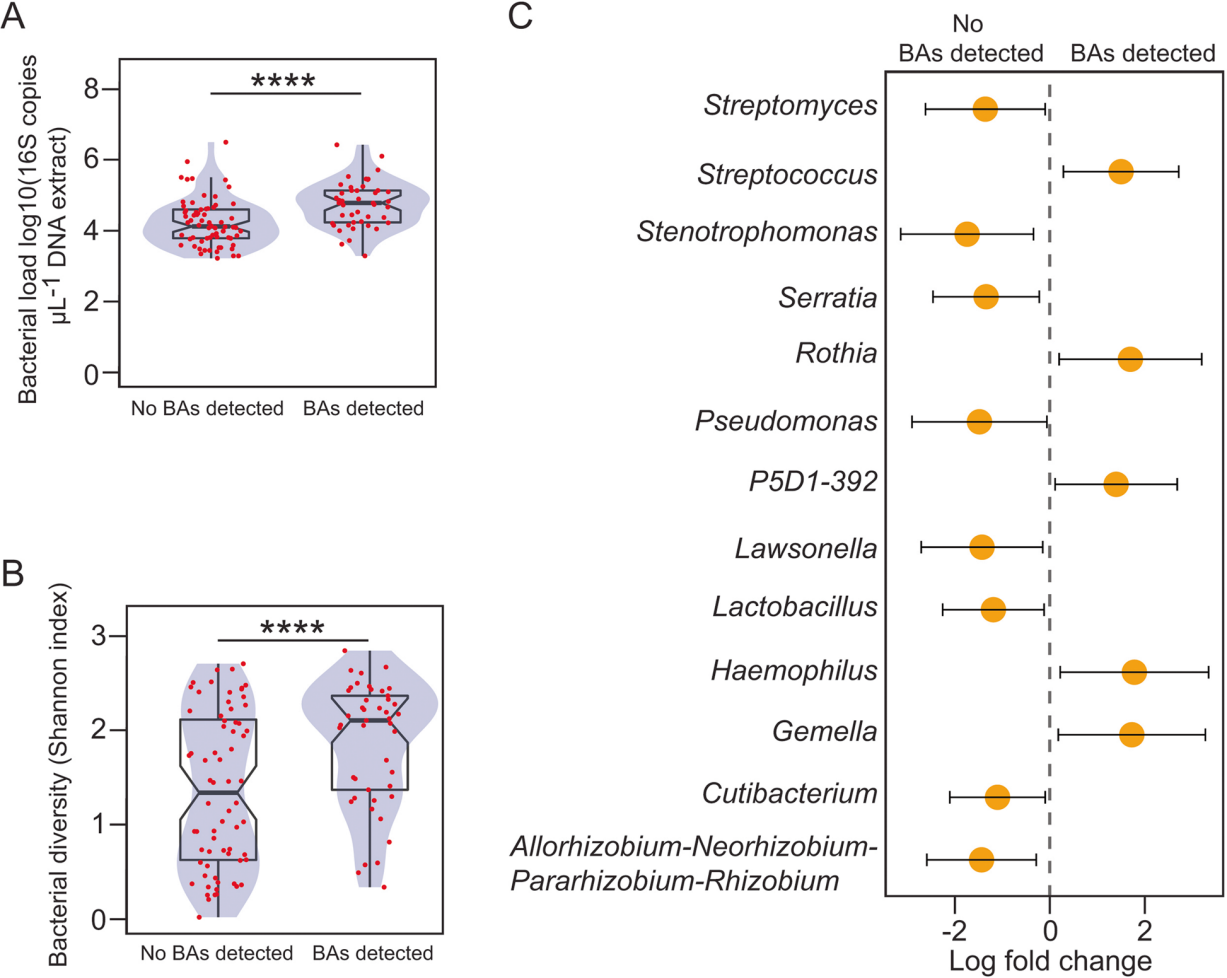
Detection of BA in BALF links to distinctive microbial profiles. Box plots overlaid with density curves (violin plots, blue) representing the bacterial burden (**A**) and bacterial diversity (**B**) with respect to the detection of BA in BALF. Individual data points (red) with jitter are represented on the top of each box plot. Notches in the boxplot represent 95% confidence interval for the median. Groups were compared using the Wilcoxon rank-sum test: ****, *p* < 0.0001. **C**. Differential abundance analysis between BALF samples with and without BA detection. Coefficients from the ANCOM-BC log linear model with pointwise 95% Bonferroni-corrected confidence intervals are plotted. Only statistically significant features are represented. Taxonomic entities enriched in BALF samples with or without BA detection are indicated with positive and negative fold change values respectively. Family *P5D1-392* represents unculturable microorganisms from the order *Lactobacillales*

Given the very young age of this cohort, we explored associations between BA detection and lung microbial profiles. BALF samples containing BA had higher bacterial burden (Log10(16S DNA copies), median [IQR], BA detected: 4.78 [4.23–5.13]; no BA detected: 4.12 [3.78–4.60]; WRST (*r* = 0.39, *p* = 0.00003)), and harboured more diverse bacterial communities (Shannon index, BA detected: 2.10 [1.37–2.37]; no BA detected: 1.34 [0.63–2.11]; WRST (*r* = 0.29, *p* = 0.002)) (Fig. 3 and Fig. S18). BALF specimens containing BA also demonstrated distinct compositional signatures typically associated with the upper respiratory tract (Fig. 3C). In line with these observations, we observed higher odds of recovering viable microorganisms from clinical microbiology cultures if BALF contained BA (Any density, odds ratio (OR) [95%CI], 2.67 [1.12–6.34],* p* = 0.03) (Fig. S19). Similar results were observed when we set the cut-off for positive culture to 10,000 colony-forming units (CFU) per mL of BALF (Any microorganism, OR 3.44 [1.55–7.66], *p* = 0.002). BA were also associated with high cell-density cultures of pathogens (Bacteria and/or Fungi) in BALF (OR 2.33 [1.09–4.96], *p* = 0.03) (Fig. S19).

### Azithromycin therapy does not affect BA detection in BALF

**Fig. 4:**
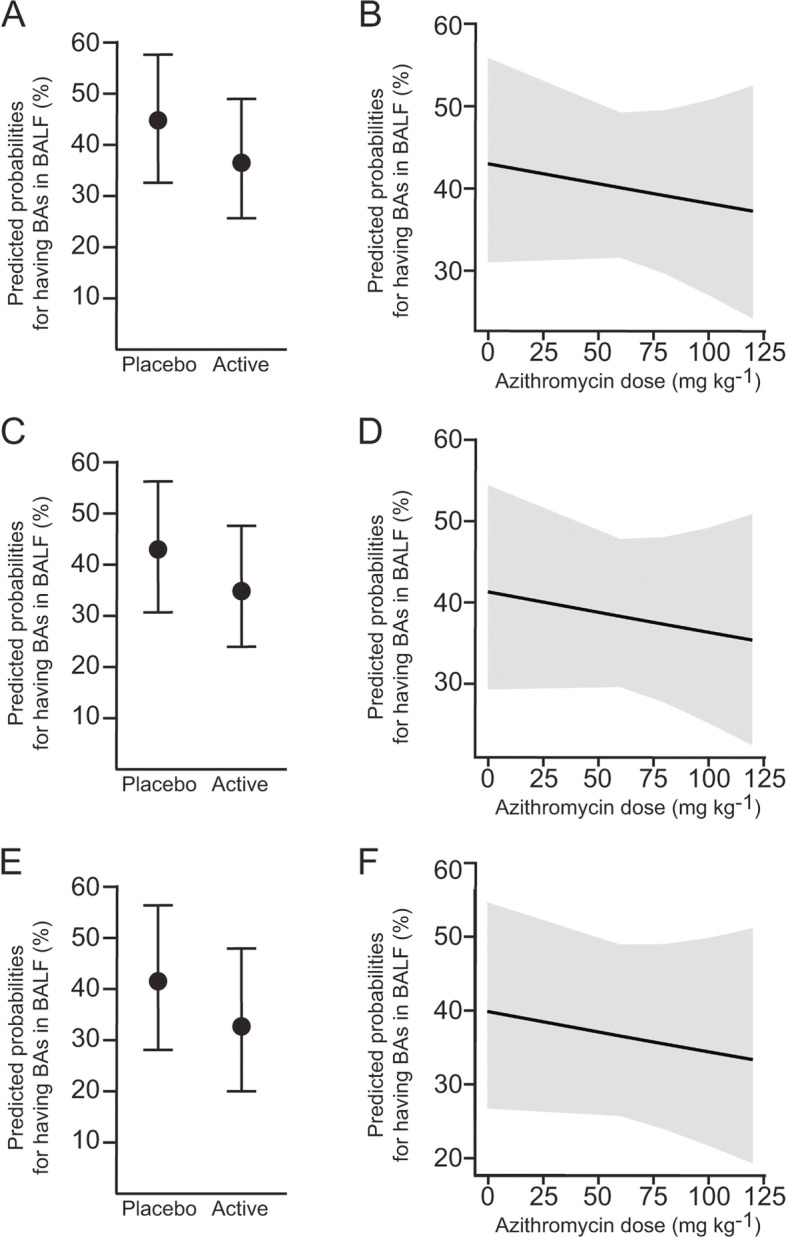
Azithromycin does not influence detection of BA in BALF. **A-F** Marginal effect of treatment arm (**A**, **C**, **E**) or azithromycin dosage (**B**, **D**, **F**), on the odds of detecting BA with pointwise 95% confidence intervals, calculated from logistic regression models. **C-F** Predicted probabilities after controlling for pancreatic insufficiency (**C-D**) or p.F508del homozigosity (**E–F**)

Azithromycin has been demonstrated to facilitate gastric emptying and reduce reflux events [17, 26, 27]. We hypothesised that if azithromycin improves gastroparesis, this therapeutic would be associated with reduced odds of detecting BA in BALF. The probability of detecting BA in BALF was neither increased in the placebo arm (OR 1.41 [0.68–2.94], *p* = 0.35), nor changed by weight-adjusted azithromycin dose at the time of BALF collection (dose (g), OR 0.14 [0–186.82], *p* = 0.59) (Fig. 4 and Fig. S20). We obtained similar results after controlling for factors related to the severity of the CF phenotype, including pancreatic insufficiency or homozygosis for the p.F508del mutation (Fig. 4 and Fig. S20).

In our cohort BA detection was more prevalent in patients from Centres who do not use anti-*Staphylococcus* prophylaxis (Fig. S21A). Patients receiving beta-lactam antibiotic prophylaxis showed a 75% relative reduction in the odds of detecting BA in BALF (OR 0.25 [0.12–0.55], *p* = 0.0005) (Fig. S21B-C and S22), as well as lower concentration of primary BA in BALF (Fig. S21E-F and S23A-C). Penicillin-based antibiotics are linked to cholestasis, which may explain these results [28]. Patients receiving anti-*Staphylococcus* prophylaxis demonstrated higher levels of serum cholestasis markers such as gamma glutamyl transpeptidase (GGT), alanine aminotransferase (ALT) and aspartate aminotransferase (AST), but not alkaline phosphatase (ALP) (Fig. S21G-J and S23D-G). In agreement with an antibiotic class-specific event, the levels of GGT, ALT, AST, ALP, as well as BA remained unaltered across azithromycin and placebo treatment arms (Fig. S24-25).

### Azithromycin does not alter the BALF microbiota in early CF

We evaluated how prophylactic antimicrobial therapies affect the composition of the BALF microbiota. Azithromycin was found not to alter bacterial load (median [IQR], azithromycin: 4.38 [4.03–4.93]; placebo: 4.44 [3.82–4.79]; WRST (*r* = 0.09, *p* = 0.31)) or diversity (azithromycin: 1.69 [0.93–2.23]; placebo: 1.73 [0.72–2.28]; WRST (*r* = 0.008, *p* = 0.93)) (Fig. S26). Similarly, clinical microbiology results were not affected by azithromycin treatment (Fig. S27). Conversely, anti-*Staphylococcal* prophylaxis at the time of bronchoscopy was associated with a reduction of bacterial biomass in BALF (median [IQR], prophylaxis: 4.14 [3.80–4.53]; no prophylaxis: 4.80 [4.34–5.26]; WRST (*r* = 0.42, *p* = 0.000009)) and diversity (prophylaxis: 1.05 [0.57–1.99]; no prophylaxis: 2.23 [1.74–2.46]; WRST (*r* = 0.56, *p* = 0.000000004)) (Fig. S28-29). Differential abundance analysis demonstrated that penicillin-type prophylaxis was associated with reduction in the proportions of oropharyngeal taxa (Fig. S30). Furthermore, the odds of retrieving positive cultures from BALF were reduced in patients taking beta-lactam antibiotics (Any culture, OR 0.33 [0.13–0.80], *p* = 0.01) (Fig. S31). We did not observe an interaction between the effects of azithromycin and anti-*Staphylococcal* prophylaxis on bacterial burden (*F*(1,108) = 0.75, *p* = 0.39) or diversity (*F*(1,108) = 0.05, *p* = 0.82) (Fig. S32).

## Discussion

In this observational study, we provide evidence supporting that detection of BAs in BALF constitutes an early predictor of both airway inflammation and clinical outcomes in CF. Specifically, we demonstrated neutrophilia and higher airway inflammation in asymptomatic infants with CF who have BAs in BALF. Our data linking BAs to disease burden suggests that profiling BAs in BALF could also help to inform clinicians of patients with worse disease trajectories, as early as at 12 months of age. We also show distinctive patterns of microbial colonisation associated with specific prophylactic antibiotic treatment as well as with detection of BAs in BALF. Thus, our study also unveils factors that should be considered when studying the relationships between the lower airway microbiota and the progression of CF lung disease.

Since the gastrointestinal environment influences the respiratory system via an uncharacterized signalisation pathway namely the gut-lung axis, it can be assumed that respiratory findings in CF patients are modulated by gastrointestinal manifestations [15]. Indeed, detection of the intestinal metabolites BA in BALF have been linked to inflammation and clinical outcomes in both CF and non-CF children [12, 13, 29], and suggest a universal pathological process that is not exclusive to CF. Furthermore, it identifies BA as important modulators in the gut-lung axis. Although determining how BA reach the airways were beyond the scope of this study, recent evidence has shown a link between delayed gastric emptying and high levels of BA in BALF [29]. It was also found that treatment with macrolides did not alter BA levels in BALF [29]. Interestingly, detection of BA in BALF was independent of reflux parameters including number of reflux episodes or type of reflux [29]. Gastrointestinal dysmotility constitutes an understudied medical problem in CF [30], and may provide an underlying mechanism facilitating the translocation of BA into the airways. We did not observe an effect of azithromycin on BA detection in BALF. Since data on gastric motility was not collected, we could not assess whether azithromycin improved gastric emptying, nor evaluate whether detection of BA in BALF was associated with parameters of gastric motility. However, azithromycin is considered an effective prokinetic agent and potent motilin agonist [17, 27]. Patients who were on antibiotic therapies known to cause cholestasis were less likely to have BA in BALF, which implies a complex interaction connecting the gastrointestinal and respiratory systems in CF. Further investigations combining both gastrointestinal and lung testing are therefore required to trace the transmission pathway in the gut-lung axis.

Determining the key processes involved in triggering the lung inflammatory response in early-life CF is a major clinical problem. Through analysis of BALF samples stratified by BA detection, we show that in CF infants, the presence of BA in BALF is strongly associated with markers of airway inflammation. However, our work does not reveal the specific role of BA in the pathophysiology of early CF lung disease, and functional studies are now required. Highlighting the complexity of the CF lung environment, BALF specimens with BA also demonstrated more diverse microbial communities enriched in oropharyngeal taxa, higher bacterial burden, and higher odds of retrieving high-cell-density microbial cultures from BALF. Thus, our findings linking BA to inflammatory markers could be confounded by alterations in the BALF-associated microbiota. While it is true that in our study, metacommunities enriched in oropharyngeal taxa exhibited higher inflammation, the effect was of lower magnitude than for BA. Furthermore, other community descriptors such as diversity or bacterial burden were either weakly associated or not correlated with markers of airway inflammation. Thus, although bacteria and BA could access lower airways via a common physiological process, our data suggest that BA themselves represent a particular pathological alteration linked to the initiation and/or maintenance of airway inflammation. Likewise, our observations support the concept that in the absence of pathogenic infections, the commensals colonising the airways may be necessary, yet insufficient for establishing the inflammatory landscape characteristic of early CF lung disease [4].

Despite the mild lung disease expected in a paediatric cohort, we observed differences in markers of disease progression related to the detection of BA in BALF. These clinical outcomes included medication-related burden, percentage of structural lung disease at the age of one, as well as the number of exacerbations during the first year of life. Elevated markers of airway inflammation provide a plausible physiological defect linking poor clinical outcomes to the presence of BA in BALF. Thus, detection of free NE activity, a precursor of bronchiectasis in CF [3], was more likely in BALF samples containing BA. Furthermore, other inflammatory markers considered predictors of pulmonary exacerbations including IL8 and percentage of neutrophils in BALF [31], were also upregulated in BALF specimens containing BA. We also observed a correlation between bacterial diversity and number of exacerbations. It has been proposed that the airway microbiota could participate in the aetiology of exacerbations by inducing episodic reconfigurations of the lung microbial communities, which subsequently dysregulates host immune responses [32]. Aspiration of oropharyngeal commensals supports this hypothesis, as they have been shown to induce both the transient rearrangement of the lower airway microbiota and a Th17-dependent tuning of host immunity [33]. In our cohort, the increase in community diversity was driven by OTUs typically associated with the oropharynx.

As CF patients age, a relationship between lung function and microbial diversity emerges [6, 8], suggesting that the initial colonisers of the airways could play a protective role. Although antibiotic treatment may confound these observations, it is important to establish whether and how these initial colonisers contribute to the pathophysiology of CF lung disease. Using metacommunity analysis we identified three bacterial community ecotypes associated with contrasting inflammatory outcomes. Interestingly, these ecological assemblies were linked to anti-*Staphylococcal* antibiotic prophylaxis or the detection of BA in BALF, but not to azithromycin. Metacommunities associated with penicillin-type prophylaxis exhibited a reduction in diversity, bacterial load, and abundance of oropharyngeal taxa, as well as with a progressive reduction in markers of airway inflammation. Ecotypes associated with the detection of BA in BALF showed opposite findings. Microaspiration is ubiquitous in healthy subjects and constitutes a physiological process for seeding the lungs with microorganisms from the upper airways [34]. Broad-spectrum penicillins could therefore have a higher bactericidal effect on these microbial colonisers. Intriguingly, BALF from infants on prophylaxis demonstrated higher proportions of *Stenotrophomonas*, and the presence of *Stenotrophomonas maltophilia* DNA was confirmed by species-specific qPCR. In our cohort, only one patient, who also was on penicillin prophylaxis, demonstrated infection by this pathogen by culture. Thus, the high incidence of *Stenotrophomonas maltophilia* DNA may be explained by transient passing, or by DNA from dead cells. We also found that beta-lactam antibiotics reduced the odds of detecting BA in BALF, likely due to a cholestatic effect. Thus, reduced inflammation in BALF from patients on penicillin prophylaxis could be secondary to keeping the airways relatively sterile and preventing the translocation of BA into the lungs. Also, our observations define early environmental attributes, which may influence colonization patterns and the microbial succession of the CF airways.

The presence of commensals in the airways prior to pathogenic infections remains controversial [4, 6, 35]. Our multicentre study supports the concept that at least in a proportion of infants with CF, complex polymicrobial communities of commensals define not only the bacterial but also the fungal component of the early lung microbiota. This is also supported by detection of non-conventional microorganisms such as *Neisseria*, *Propionibacterium acnes* or *Corynebacterium* in bacterial cultures from BALF (Table S4). Using molecular methods, we found that bacterial burden positively correlated with diversity metrics. The estimated load of oropharyngeal commensals but not traditional CF pathogens drove this relationship. We also observed that broad-spectrum penicillins significantly impacted the load and diversity of non-traditional bacterial taxa, suggesting that at least early in life and contrary to previous observations [35], they do not represent background contaminants. We profiled fungal communities in only ~ 31% of the BALF specimens, likely due to the low fungal biomass in the DNA extracts. As a result of this low sample size, we did not evaluate associations between the resulting mycobiome and clinical outcomes. We did, however, observe a significant drop in fungal diversity in specimens from patients on penicillin-type antibiotics, suggesting that the integrity of the early airway mycobiome may depend on inter-kingdom ecological interactions. As previously reported, we observed a drop in bacterial diversity and airway inflammation associated with anti-*Staphylococcal* prophylaxis [36]. We also noted that prophylactic administration of broad-spectrum penicillins correlated with a reduction in bacterial burden and lower odds of culturing microorganisms from BALF. Conversely, diversity and bacterial biomass remained unaltered by treatment with azithromycin. This result was also supported by our culture-based clinical data and agrees with prior work in older patients with CF [37]. Basal resistance to macrolides by microorganisms found in BALF could explain these unexpected observations [38] but suggests that benefits associated with azithromycin therapy early in life are independent of its antimicrobial properties.

Our study has several limitations. Firstly, it was impossible to determine whether airway exposure to BA preceded the clinical and microbiological outcomes described in this work. Thus, causal inferences cannot be drawn, and further functional studies are required for this purpose. However, associations between BA in BALF and inflammation have been replicated in non-CF patients, validating observations made here [29]. Secondly, uncertainty due to confounding bias is unavoidable in observational studies. However, we expect that unmeasured confounders associated with inflammation and lung function, were minimised by the multi-centre character of our study and the young age of our patient cohort. We have also been stringent in controlling for type-1 error when performing multiple testing. Importantly, azithromycin treatment was controlled by randomisation, thus minimising uncertainty due to confounding by random distribution of risk factors. Thirdly, BALF was collected using laryngeal masks under general anaesthesia, which does not completely rule out the possibility of salivary contamination. However, the pooled second and third lavages were used for profiling BA and extracting microbial DNA. Thus, if secretions from the upper airways entered the bronchoscope during the procedure, they would have been diluted and/or washed out with the first lavage. Finally, molecular methods do not confirm the viability of the reported BALF microbiota. Although we observed a good correspondence between molecular and culture approaches, clinical culture is biased towards medically relevant species, and most of the taxa we observed are commensals that may require specific growing conditions.

## Conclusion

Collectively, our data implies that BA either contribute to or predict early pathomechanisms involved in establishing both the microbial and inflammatory landscapes in CF lungs early in life. Although the use of a potent prokinetic does not reduce BA levels in the lower airways, other strategies to modulate the lung exposure to BA should be explored. Furthermore, the identification of non-invasive biomarkers of BA exposure could provide useful tools to predict infants at greatest risk of progressive lung disease.

## Methods

### Clinical trial details

We used the BALF specimens from 12 months old infants with Cystic Fibrosis (CF) enrolled in the COMBAT-CF clinical trial. COMBAT-CF was a multi-centre, double-blinded, randomised, placebo-controlled study to evaluate the efficacy of azithromycin for the primary prevention of bronchiectasis in children with CF [16]. Participants were randomized in blocks using a one-to-one ratio between treatment and placebo arms. Randomisation was stratified by site using an interactive web-based response system. Patients were administered with 10 mg kg^−1^ of azithromycin (Zithromax® oral suspension as 40 mg mL^−1^) or matched placebo three times per week. Weight-adjusted dosing regimens were provided at the monthly clinic visits. During the study, all the patients received standard CF therapy as recommended in each study centre, including those antibiotic regimens to eradicate bacteria found in BALF culture.

### Isolation of microbial DNA from BALF, 16S and ITS2 metabarcoding, and sequencing of the amplicon pools

Microbial DNA was isolated from (1–2 mL) of BALF supernatant using our previously published protocol, which was specifically designed to improve the recovery of microbial DNA from low biomass BALF samples [39]. Extraction of DNA, library preparation and sequencing were performed blinded to clinical data. Libraries for bacterial taxonomic profiling using V3-V4 16S rRNA region and sequencing of the amplicon products were performed at Genewiz as we described [39]. For the 16S library, we obtained 7,441,469 paired-end reads with a high base calling accuracy (> 94% of sequences with mean Phred-like Q-score greater than or equal to 30).

To identify and characterize the fungal population associated with the BALF specimens, we carried out fungal DNA barcoding using the internal transcribed spacer (ITS) 2 region. Amplicon libraries were constructed at Genewiz (China) using forward and reverse primers containing the sequences *5’-GTGAATCATCGARTC* and *5’-TCCTCCGCTTATTGAT* respectively. The specific primers were designed to contain adaptor sequences to allow uniform amplification of the library with high complexity. Libraries were validated by Agilent 2100 Bioanalyzer, quantified using Qubit 2.0 Fluorometer, multiplexed and sequenced on a MiSeq® Instrument (Illumina) using a 2 × 300/250 paired-end configuration. For the ITS2 library, we obtained 5,619,475 paired-end reads with a good base calling accuracy (> 80% of sequences with mean Phred-like Q-score greater than or equal to 25).

### Processing pipeline of the sequencing data

Quality-based pre-processing and merging of paired-end reads for all the sequencing data files was as we described [39]. This step resulted in 6,336,505 single reads (average length 426 bp) for the 16S rRNA dataset, with 99.8% of the reads showing a mean Phred-like Q-score greater than or equal to 30. In the case of the ITS2 dataset, we obtained 2,284,945 single reads (average length 340 bp), with 99.8% of the reads showing a mean Phred-like Q-score greater than or equal to 30.

For the 16S rRNA amplicon sequencing data, pre-processed joint reads were analysed following the SILVAngs pipeline [40, 41]. Briefly, merged reads were aligned by the SILVA Incremental Aligner (SINA) [42] against the SILVA SSU rRNA SEED and quality controlled [41]. We filtered out not aligned sequences or sequences with low quality alignment (less than 50% alignment identity and alignment score of 40 as calculated by SINA), as well as reads shorter than 200 bp and with more than 2% ambiguities or 2% homopolymers. Reads were clustered at 98% sequence identity using VSEARCH (Version 2.15.1) [43]. Classification of the representative read from each cluster against the last release of the standardised SILVA SSU taxonomy (release 138.1) was performed implementing a BLAST-based approach and using the best BLAST hit. To avoid spurious classifications, best hits were only accepted if “(% sequence identity + % alignment coverage)/2" was higher than 93. This is an empirically determined threshold that provides a good compromise between false negative and false positive classification likelihood. Sequences with no or weak BLAST hit as determined by the threshold above, were considered unclassified and identified as “No Relative” in the taxonomy table. Less than 1% of the reads of the final dataset were defined as “No Relative” under these conditions. Following this workflow, reads were assigned to 956 Operational Taxonomic Units (OTUs).

Processing and taxonomic assignment of the ITS2 reads was conducted in R (version 4.0.2) using the *DADA2* (version 1.18.0) pipeline [44]. De novo chimera detection and removal were done by consensus across samples using the *removeBimeraDenovo* function as implemented in *DADA2*. Contigs were mapped to the UNITE 8.2 ITS database (sh_general_release_dynamic_04.02.2020) [45], using the naïve Bayesian Classifier algorithm described by Wang [46]. Amplicon sequence variants (ASVs) were taxonomically identified based on the UNITE ITS taxonomy.

As expected, a significantly higher number of classified 16S and ITS reads in DNA extracts from the BALF specimens compared to the negative extraction controls was observed (Fig. S33A-B).

### Removal of the potential background contaminants determined by negative extraction controls

The susceptibility of contamination in low-biomass samples such as those used in this study, and the resulting implications for producing biologically meaningful datasets, have stimulated the development of guidelines and recommendations to mitigate the influence of contaminants in the interpretability of 16S-based microbial profiles [47, 48]. We controlled for environmental-, instrumentation- and reagent-related contaminants by processing and sequencing extraction batch negative controls as well as saline washes of the bronchoscope. This approach is in agreement with recommended standards for the analysis of microbial communities in low-biomass environments [47, 48]. We initially explored the impact of contaminants in the BALF raw taxonomic profiles in two ways. Firstly, using principal component analysis (PCA) we found that both negative batch extraction controls and bronchoscope washes clustered away from BALF samples (Fig. S33C-D). This observation is consistent with BALF-associated profiles representing genuine communities. Second, we conducted Procrustes analysis of the PCA ordination of the microbial community composition between BALF samples and their paired bronchoscope wash controls. This analysis demonstrated a low correlation between datasets in the bacterial taxonomic profiles (Bacteria, M^2^ = 0.74, correlation in a symmetric rotation = 0.51, *p* < 0.05) (Fig. S34 and Table S7).

For the 16S datasets, the bacterial burden and the taxonomic profiles of these technical controls were subsequently used to eliminate the bias introduced by potential contaminants in the BALF 16S-based microbial profiles. The quantification of the nucleic acids presented in these control samples allowed estimating the limit of detection to discern true biological signal from background noise. We established the background cut-off to 1376.61 16S DNA copies µL^−1^ of DNA extract, which lies three standard deviations from the mean bacterial load in the controls (770.74 16S DNA copies µL^−1^ DNA extract) (Fig. S5). Consistently with a true biological signal, 113 out of the 121 BALF DNA extracts contained bacterial DNA levels above this background threshold. Then we excluded from the taxonomic table potential PCR artefacts represented by singletons, classified as Chloroplast or Mithochondria and no classified reads. The later contributed to a small proportion of the total counts in the dataset (mean relative abundance no classified 0.0006%, standard deviation 0.001; mean relative abundance Chloroplast 0.006%, standard deviation 0.03; mean relative abundance Mithocondria 0.0001%, standard deviation 0.0007). Low-count taxa (OTUs representing less than 0.01% across all samples) were also removed. These filtering steps reduced the initial number of OTUs from 956 to 98. To detect potential contaminants introduced during the collection and/or processing steps of the BALF specimens, we implemented the R package *decontam* as we described [39]. Four OTUs were identified as potential contaminants and thus they were fully removed from the taxonomic profiles (Table S7). Procrustes analysis demonstrated that these filtering steps did not significantly alter the overall community structure of the original (unfiltered) dataset (Table S8 and Fig. S35A-B).

For the ITS2 sequence data we proceeded as follows. We removed reads assigned only at Kingdom level (10.77% of the total reads) as well as low count ASVs (ASVs with less than 0.01% abundance across the whole dataset), resulting in a reduction from 2331 to 596 ASVs. Implementation of *decontam* algorithm resulted in the removal of additional 24 ASVs putatively arising from contamination (Table S9-10). Finally, ASVs counts were aggregated by taxonomic classification, resulting in 292 unique taxonomic paths. Procrustes analysis suggested that these pre-processing steps did not remarkably impact the composition of the original fungal profiles (Table S11 and Fig. S35C-D).

### Quantitative analysis using TaqMan® assays

The protocol for qPCR using TaqMan® probes, as well as the assays for quantifying *Stenotrophomonas malthophilia* genomic content and total bacteria load in DNA extracts can be found in our previous publication [39]. The levels of *Stenotrophomonas maltophilia*, as well as the correspondence between the estimated and absolute abundance of this microorganisms can be found in Fig. S36. The TaqMan® probe against *Malasezzia restricta* was based on a previous report [49]. Standard curve for *Malasezzia restricta* was generated using ten-fold dilutions of the following gBlocks™ Gene Fragment (IGT): *5’- gtgattgcagattccgtgaatcatcagaatctttgaacgcaccttgcgctctatggcaatccgtagagcatgcccgtttgagtgccatgaaatctcccaccccaagcggtttttaaatgaaacggcttggcggatggggtctggatgggtgcctctgcctgcgctacctagcacaggctcgcccgaaatgcatgagcgccttgagacactttgcatccgcctctctgtttgggaggaggcggccaagcagtgtttttctcctggcatggcatgatacgtcatttgctatgtcacctaaaggaggaatgtttggttgtgtctgcgtgtgcttcaaacttgcctctgtggcacatcccaatttcacttctggtctcaaatcaggtaggatcacccgctgaacttaagcatatcaataagcggaggaaa.* Details about this assay are in in Table S12.

### Bile acid profiling in BALF

Bile acids were profiled from the supernatants obtained after pelleting microbial cells for DNA extraction using high-speed centrifugation and blinded to the associated clinical data. Samples were spiked with 500 pmol of each internal standard and alkalinized with 0.1 volumes of 1 M NaOH solution. Extraction of bile acid analytes was achieved through solid-phase extraction using OASIS HLB® reversed-phase sorbent (Waters, batch 163A). Cartridges containing 60 mg of OASIS® HLB sorbent were conditioned with 1 mL of methanol and then with 3 mL of water. Samples were passed twice through the column, after which two washes with 1 mL of water were performed. Retained analytes were then eluted with 1 mL of 90% acetonitrile in water, dried in a stream of nitrogen and reconstituted with 50% methanol in water. Ultra-high performance liquid chromatography-quadrupole time-of-flight mass spectrometry and bile acid identification were performed as we previously described [50].

For quantifying bile acid load in BALF we spiked the samples with a mix of deuterized internal standards 5β-cholanic acid-3α,12α-diol-2,2,4,4-d4 (CA-D4, C1070-015 Steraloids), and 5β-cholanic acid-3α,7α-diol-2,2,4,4-d4 (CDCA-D4, C0940-015 Steraloids) and one analogous BA 23-Nor-5β-cholanic acid-3α, 7α, 12α triol (23-NCA, N2450-000 Steraloids). To quantitate those bile acid species for which no deuterium-labelled internal standard was available we used 23-NCA. Quantification represents the ratio between the peak area of the analyte and the peak area of the internal standard.

The odds of detecting BAs did not change across batches, except for batch 4 in which we observed a significantly reduced probability of detecting BAs with respect to the reference batch (batch 1) (OR (95% confidence interval), 0.18(0.04–0.76), *p*-0.022) (Fig. S37). To exclude the possibility of batch 4 influencing the observed associations of this report, a sensitivity analysis excluding the samples of this batch was also run to confirm our findings. Figs. S1, S3, S18, S20, S22, S23 and S25 show the results of these analyses.

### Statistical analyses

Statistical analyses were done in R (Version 4.0.2). Normal distribution of the data was examined using the Shapiro–Wilk test. When the normality assumption was not met, we implemented non-parametric tests to compare groups. Linear models were fit with the R built-in function *lm*. We used regression diagnostics to confirm normal distribution and homoscedasticity of residuals, and the absence of high-leverage data points. Two-way ANOVA was calculated using Type III Sums of Squares using the function *Anova* of the R package *car*. Microbial community descriptors were calculated using the R package *vegan*. Differential abundance analysis was performed using ANCOM-BC [51]. Cluster analysis of the BALF microbial communities was done using Dirichlet multinomial mixture models [25]. The full list of R packages used in this study can be found in Table S13. The probability threshold for statistical significance was set at 0.05.

## Supplementary Information

**Additional file 1: Figure S1.** Related to Figure 1. Sensitivity analysis controlling for potential batch effects in bile acid detection. See material and methods and Figure S37. A-B.Box plots overlaid with density curves (violin plots, blue) representing the proportion neutrophils (A) and the levels of IL8 (B) with respect to the detection of BAs in BALF. Individual data points (red) with jitter are represented on the top of each box plot. Groups were compared using the Wilcoxon rank-sum test: ****, *p*<0.0001. **Figure S2.** Box plot overlaid with density curves (violin plots, blue) representing Neutrophil elastase (NE) activity with respect to the detection of BAs in BALF. Individual data points (red) with jitter are represented on the top of eachplot. **Figure S3.** Related to Figure 1C-H and Figure S4. Sensitivity analyses controlling for potential batch effects. See material and methods and Figure S37. A-F. Boxplots overlaid with density curves (violin plots, blue) representing the proportion of the lung with structural disease (A), the number of pulmonary exacerbations (B), the number of days hospitalised in the case of a pulmonary exacerbation (C), the number of oral/inhaled antibiotic rounds (D), theaveraged number of days (Log10 transformed) treated with oral/inhaled antibiotics (E), and the number of intravenous antibiotics rounds (F), during the first year of life with respect to the detection of BAs in BALF. Individual data points (red) with jitter are represented on the top of each box plot. The proportion of the lung with structural damage in A was determined from CT scans using the PRAGMA scoring system [1]. The data represented in E includes the total duration of any treatment started before the collection of the BALF samples. Groups were compared using the Wilcoxon rank-sum test: ***, *p*<0.001; **, *p*<0.01; *, *p*<0.05; n.s., no significant (*p*>0.05). **Figure S4.** A-B. Box plots overlaid with density curves (violin plots, blue) representing the number of days hospitalised in the case of a pulmonary exacerbation (A), and the number of intravenous antibiotics rounds (B), during the first year of life with respect to the detection of BAs in BALF. Individual data points (red) with jitter are represented on the top of each box plot. Groups were compared using the Wilcoxon rank-sum test: n.s., no significant (*p*>0.05). **Figure S5.** The dot plot shows the quantification by qPCR of the bacterial burden in the DNA extracts from the indicated sample type. The mean bacterial load in the controls (including bronchoscope washes and negative extraction controls) is indicated with a horizontal red line. The horizontal black line lays 3 standard deviations from the mean bacterial load in the controls (red line), and it was set as the lower limit of detection in our cohort. **Figure S6.** A-B. Bar plots representing the bacterial compositional profiles of the BALF samples as relative proportions (A), or as normalized levels (relativeproportions multiplied by the total bacterial load of each sample) (B). Only the top 11 OTUs are represented. Specimens are grouped per collection Centre. Centres performing anti-*Staphylococcus *prophylaxis are labelled in red. **Figure S7.** Barplot represents the mean and standard deviation for the top 11 bacterial OTUs at genus level resolution. **Figure S8.** A-E. Linear relationship between microbial diversity (Shannon index) and total bacterial burden (A), and estimated pathogen (B) or oral bacteria (C) load in bacterial DNA extracts from BALF. D. Dot plot showing the association between microbial diversity and absolute abundance of *Stenotrophomonas maltophilia* in BALF. Line of best fit for each linear model is plotted with 95% confidence interval. Estimated absolute abundance in (B) and (C) was calculated by multiplying the relative abundance of each bacterial group by the total bacterial load obtained through qPCR. We considered the following OTUs as CF pathogens (*Stenotrophomonas*, *Haemophilus*, *Staphylococcus*, *Pseudomonas*,* Bordetella*, *Escherichia-Shigella*) [2]. Oral bacteria group represents the following taxonomic entities (*Streptococcus*, *Neisseria*, *Veillonella*, *Alloprevotella*, *Gemella*, *Rothia*, *Porphyromonas*, *Granulicatella*, *Prevotella_7*, *Leptotrichia*), which were found at least at 1% relative abundance in our dataset. The regression coefficient,coefficient of determination, and the results of the F-test for each model are shown on the top of each plot. **Figure S9.** A-D. Dot plots representing the relationship between inflammatory markers and microbial diversity (A-B) or bacterial biomass (C-D) in BALF. Foreach linear model, we have plotted the best-fit regression line with 95%confidence interval. The regression coefficient, coefficient of determination, and the results of the F-test for each model are shown on the top of each plot. **Figure S10.** A-B. Marginal effect of bacterial diversity (A) and bacterial burden (B) in BALF on the number of exacerbations with pointwise 95% confidence intervals, calculated from a Poisson regression model. Accordingly with the model in A, for one unit increase in diversity there was 1.37 (95% confidence interval1.12-1.70, *p*=0.003) times more exacerbations. We did not observe a relationship between the number of exacerbations during the first year of life and the bacterial load (number of exacerbations per unit increase in bacterial load 1.15, 95% confidence interval 0.93-1.43, *p*=0.20). C-D. Dot plots representing the relationship between percentage of structural lung disease and microbial diversity (C) or bacterial biomass (D) in BALF. For each linear model, we have plotted the best-fit regression line with 95% confidence interval. The regression coefficient, coefficient of determination, and the results of the F-test for each model are shown on the top of each plot. **Figure S11.** A-B. Bar plots representing the fungal compositional profiles of theBALF samples as relative proportions at specie (A), and at phylum levels (B). Only the top 11 ASVs are represented. Specimens are grouped per collection centre. Centres performing anti-*Staphylococcus *prophylaxis are labelled in red. C-D. Barplots represent the mean and standard deviation for the top 11 ASVs at species level resolution (C), or for the top 2 phyla (D). **Figure S12.** A. Quantification of *Malassezia restricta* genetic material in the DNA extracts from the indicated sample type. C-D. Box plots overlaid with density curves (violin plots, blue) representing the load of *Malassezia restricta* in BALF with respect to the samples that yielded ITS2 library amplification (B), the anti-staphylococcal regimen at the time of BALF collection (C), or the treatment arm (azithromycin or placebo) (D). Individualdata points (red) with jitter are represented on the top of each box plot. Notches in the boxplot represent 95% confidence interval for the median. Groups were compared using the Wilcoxon rank-sum test: ****, *p*<0.0001; n.s., no significant (*p*>0.05). Conditioned probabilities in A were corrected using the false discovery rate method. **Figure S13.** A-B. Box plots overlaid with density curves (violin plots, blue) representing the fungal diversity (Shannon Index) in BALF with respect to the anti-staphylococcal regimen at the time of BALF collection (A), or the treatment arm (azithromycin or placebo) (B). Individual data points (red) with jitter are represented on the top of each box plot. Groups were compared using the Wilcoxon rank-sum test: *, *p*<0.05; n.s., no significant (*p*>0.05). **Figure S14.** Goodness-of-fit measures Bayesian Information Criterion (BIC), Akaike Information Criterion (AIC) and Laplace approximation (Laplace) were use to evaluate the fit of each number of microbial communities to the 16S-based microbial taxonomic profiles. **Figure S15.** Barplots represent the contribution of the indicated taxonomic groups to each metacommunity. **Figure S16.** Box plots overlaid with density curves (violin plots, blue) representing the bacterial burden (A) and diversity (B), and the density of  *Stenotrophomonas maltophilia* in the DNA extracts from BALF. Individual data points (red) with jitter are represented on the top of each box plot. Notches in the boxplot represent 95% confidence interval for the median. Groups were compared using the Wilcoxon rank-sum test and p-values adjusted for multiple comparisons usingthe Bonferroni correction: ****, *p*<0.0001;***, *p*<0.001; **, *p*<0.01; *, *p*<0.05. **Figure S17.** A-C. Percent stacked barcharts representing the proportion of BALF samples within each microbial community type collected from patients being treated with penicillin-type antibiotics (A) or azithromycin (B) at the time ofBALF collection, or being positive for bile acid detection (BAs) (C). A Fisher’s exact test of independence was used to compare the proportions of BALF samples within each community type. P-values are adjusted using the Bonferroni correction: ****, *p*<0.0001; ***, *p*<0.001; **, *p*<0.01; *, *p*<0.05; n.s., no significant (*p*>0.05). **Figure S18.** Related to Figure 3. Sensitivity analysis controlling for potential batch effects. See material and methods and Figure S37. A-B. Box plots overlaid with density curves (violin plots, blue) representing the bacterial diversity (A) and burden (B) in BALF with respect to treatment with penicillin-like antibiotics. Individual data points (red) with jitter are represented on the top of each box plot. Notches in the boxplot represent 95% confidence interval for the median. Groups were compared using the Wilcoxon rank-sum test: ***, *p*<0.001; **, *p*<0.01. **Figure S19.** The forest plot graphs the association between the detection of bile acids in BALF and the recovery of viable microorganisms using culture-based methods at any density, or at a density equal or higher than 10,000 cfu. Odds ratios with pointwise 95% confidence intervals are plotted. Oral-like flora represents the growth of these microorganisms: Mixed oral flora, *Streptococcus*, *Streptococcus salivarius*, *Streptococcus viridans*, *Neisseria*, *Rothia mucilaginosa*, *Propionibacterium acnes*, *Corynebacterium*. Bacterial pathogensgroup represents the following microorganisms: methicillin-resistant *Staphylococcus aureus*, *Pseudomonas aeruginosa*, *Moraxella catharralis*, *Streptococcus pneumoniae*, *Stenotrophomonas maltophilia*, *Staphylococcus aureus*, *Haemophilus influenza*, *Escherichia coli*, *Kebsiella oxytoca*, coagulase negative *Staphylococcus*, *Staphylococcus*, *Haemophilus*, *Haemophilus parainfluenzae*, mixed gram negative, *Enterobacter cloacae*, *Citrobacter freundii*, *Sphingomonas paucimobilis*, *Serratia marescens*. The following microorganisms are considered as fungal pathogens: *Candida albicans*, *Candida sp*, *Aspergillus*, *Aspergillus fumigatus*. **Figure S20.** Related to Figure 4. Sensitivity analysis controlling for potential batch effects. See material and methods and Figure S37. A-F. Marginal effect of treatment arm (A, C, E) or azithromycin dosage (B, D, F), on the odds of detecting bile acids with pointwise 95% confidence intervals, calculated from logistic regression models. C-F. Predicted probabilities after controlling for pancreatic insufficiency (C-D) or p.F508del homozigosity (E-F). **Figure S21.** A. Percentage of BALF samples with bile acid detection in each participating Centre. Each Hospital is identified with a three-letter code. Centres routinely performing anti-*Staphylococcus* prophylaxis are labelled in blue. Centres not administering penicillin-type prophylaxis are depicted in red.  The number of BALF specimens collected from each Centre is indicated in white over the corresponding bar. B-C. Marginal effect of anti-*Staphylococcus* prophylaxis (B) and specific penicillin-type antibiotic regimens (C), on the odds of detecting bile acids with pointwise 95% confidence intervals, calculated from logistic regression models. P-values in C represent the level of statistical significance using a Wald test for the difference in log odds between each category and the reference group (Augmentin). ***, *p*<0.001; n.s., no significant (*p*>0.05). D-J. Box plots overlaid with density curves (violin plots, blue) representing the concentration of bile acids in BALF (D-F), and the levels of circulating markers of liver damage (G-J) related to anti-*Staphylococcus* prophylaxis at the time of BALF collection. Concentrations of primary and secondary bile acids are represented in E and F respectively.  Individual data points (red) with jitter are represented on the top of each box plot. Notches in the boxplot represent 95% confidence interval for the median. In D-J, groups were compared using the Wilcoxon rank-sum test. ****, *p*<0.0001; **, *p*<0.01; n.s., no significant (*p*>0.05). Abbreviations: ADL, Women’s and Children’s Hospital Adelaide; PER, Princess Margaret Hospital Perth; MRC, Royal Children’s Hospital Melbourne; SSC, Sydney Children’s Hospital; SWC, The Children’s Hospital at Westmead Sydney; MMC, Monash Medical Centre Melbourne;BRC, Royal Children’s Hospital Brisbane; BMC, Mater Children’s Hospital Brisbane; LCC, Queensland Children’s Hospital Brisbane; NZA, Starship Children’s Hospital Auckland; GGT, gamma glutamyl transpeptidase; ALT, alanineaminotransferase; AST, aspartate aminotransferase; ALP, alkaline phosphatase. **Figure S22.** Related to Figure S20B. Sensitivity analysis controlling for potential batch effects. See material and methods and Figure S37. Marginal effect of anti-*Staphylococcus* prophylaxis on the odds of detecting bile acids with pointwise 95% confidence intervals, calculated from logistic regression models. **Figure S23.** Related to Figure S20D-J. Sensitivity analysis controlling for potential batch effects. See material and methods and Figure S37. A-G. Box plots overlaid with density curves (violin plots, blue) representing the concentration of bile acids in BALF (A-C), and the levels of circulating markers of liver damage (D-G) related to anti-*Staphylococcus *prophylaxis at the time of BALF collection. Concentrations of primary and secondary bile acids are represented in B and C respectively.  Individual data points (red) with jitter are represented on the top of each box plot. Notches in the boxplot represent 95%confidence interval for the median. Groups were compared using the Wilcoxon rank-sum test. ***, *p*<0.0001; **, *p*<0.01; *, *p*<0.05; n.s., no significant (*p*>0.05). Abbreviations: GGT, gamma glutamyl transpeptidase; ALT, alanine aminotransferase; AST, aspartate aminotransferase; ALP, alkaline phosphatase. **Figure S24.** A-G. Box plots overlaid with density curves (violin plots, blue) representing the concentration of bile acids in BALF (A-C), and the levels of circulating markers of liver damage (D-G) related to the treatment arm (azithromycin or placebo). Concentrations of primary and secondary bile acids are represented in B and C respectively. Individual data points (red) with jitter are represented on the top of each box plot. Notches in the boxplot represent 95% confidence interval for the median. Groups were compared using the Wilcoxon rank-sum test: n.s., no significant (*p*>0.05). Abbreviations: GGT, gamma glutamyl transpeptidase; ALT, alanine aminotransferase; AST, aspartate aminotransferase; ALP, alkaline phosphatase. **Figure S25.** Related to Figure S18. Sensitivity analysis controlling for potential batch effects. See material and methods and Figure S37. A-G. Box plots overlaidwith density curves (violin plots, blue) representing the concentration of bile acids in BALF (A-C), and the levels of circulating markers of liver damage (D-G) related to the treatment arm (azithromycin or placebo). Concentrations of primary and secondary bile acids are represented in B and C respectively.  Individual data points (red) with jitter are represented on the top of each box plot. Notches in the boxplot represent 95% confidence interval for the median. Groups were compared using the Wilcoxon rank-sum test: n.s., no significant (*p*>0.05). Abbreviations: GGT, gamma glutamyl transpeptidase; ALT, alanine aminotransferase; AST, aspartate aminotransferase; ALP, alkaline phosphatase. **Figure S26.** A. Principal component analysis (PCA) plot shows the linear projection of the 16S-based compositional profiles onto a two-dimensional space. Eachsample is labelled based on the treatment arm membership: azithromycin (blue circle) or placebo (orange triangle). B. Loadings for PC1 representing the weights of the indicated variables defining component 1. C-D. Box plots overlaid with density curves (violin plots, blue) representing the bacterial diversity (C) and burden (D) in BALF with respect to the treatment arm (azithromycin or placebo). Individual data points (red) with jitter are represented on the top of each box plot. Notches in the boxplot represent 95%confidence interval for the median. Groups were compared using the Wilcoxon rank-sum test: n.s., no significant (*p*>0.05). **Figure S27.** The forest plot graph the association between azithromycin therapy and the recovery from BALF of viable microorganisms using culture-based methods at any density, or at a density equal or higher than 10,000 cfu. Odds ratios with pointwise 95% confidence intervals are plotted. Oral-like flora represents the growth of these microorganisms: Mixed oral flora, *Streptococcus*, *Streptococcus salivarius*, *Streptococcus viridans*, *Neisseria*, *Rothia mucilaginosa*, *Propionibacterium acnes*, *Corynebacterium*. Bacterial pathogens group represents the following microorganisms: methicillin-resistant *Staphylococcus aureus*, *Pseudomonas aeruginosa*, *Moraxella catharralis*, *Streptococcus pneumoniae*, *Stenotrophomonas maltophilia*, *Staphylococcus aureus*, *Haemophilus influenza*, *Escherichia coli*, *Kebsiella oxytoca*, coagulase negative *Staphylococcus*, *Staphylococcus*, *Haemophilus*, *Haemophilus parainfluenzae*, mixed gram negative, *Enterobacter cloacae*, *Citrobacter freundii*, *Sphingomonas paucimobilis*, *Serratia marescens*. The followingmicroorganisms are considered as fungal pathogens: *Candida albicans*, *Candida sp*, *Aspergillus*, *Aspergillus fumigatus*. **Figure S28.** A. Principal component analysis (PCA) plot shows the linear projection of the 16S-based compositional profiles onto a two-dimensional space. Each sample is labelled based on anti-Staphylococcus prophylaxis regimen at the time of BALF collection: prophylaxis (blue circle) or no prophylaxis (orange triangle). B. Loadings for PC1 representing the weights of the indicated variables defining component 1. C-D. Box plots overlaid with density curves (violin plots, blue) representing the bacterial diversity (C) and burden (D) in BALF with respect to treatment with penicillin-like antibiotics. Individual data points (red) with jitter are represented on the top of each box plot. Notches in the boxplot represent 95% confidence interval for the median. Groups were compared using the Wilcoxon rank-sum test: ****, *p*<0.0001. **Figure S29.** A. Principal component analysis (PCA) plot shows the linear projection of the 16S-based compositional profiles onto a two-dimensional space. Each sample is labelled based on the type of penicillin-like therapy at the time of BALF collection. “Both” refer to flucloxacillin and augmentin regimen B-C. Boxplots representing total bacterial burden (B) and the microbial diversity (C) in BALF with respect to the type of penicillin-like antibiotic taken. Notches represent 95% confidence interval for the median. Groups were compared using one-way ANOVA and *p*-values estimated with Dunnett’s posthoc test: ****, *p*<0.0001; ***, *p*<0.001; **, *p*<0.01. **Figure S30.** The dotplot displays the results of the differential abundance analysis between BALF-associated bacterial communities from patients taking anti-staphylococcal prophylaxis or not at the time of BALF collection. Coefficients from the ANCOMBC log linear model with pointwise 95% Bonferroni-corrected confidence intervals are plotted. Only statistically significant features with an absolute Log fold change value higher than 1.8 arerepresented. Taxonomic entities enriched in BALF samples from patients taking or not penicillin-like antibiotics at the time of BALF collection are represented with positive and negative fold change values respectively. **Figure S31.** The forest plot graph the association between the penicillin-type prophylaxis and the recovery from BALF of viable microorganisms using culture-based methods at any density, or at a density equal or higher than 10,000 cfu. Odds ratios with pointwise 95% confidence intervals are plotted.Oral-like flora represents the growth of these microorganisms: Mixed oral flora, *Streptococcus*, *Streptococcus salivarius*, *Streptococcus viridans*, *Neisseria*, *Rothia mucilaginosa*, *Propionibacterium acnes*, *Corynebacterium*. Bacterial pathogens group represents the following microorganisms: methicillin-resistant *Staphylococcus aureus*, *Pseudomonas aeruginosa*, *Moraxella catharralis*, *Streptococcus pneumoniae*, *Stenotrophomonas maltophilia*, *Staphylococcus aureus*, *Haemophilus influenza*, *Escherichia coli*, *Kebsiella oxytoca*, coagulase negative *Staphylococcus*, *Staphylococcus*, *Haemophilus*, *Haemophilus parainfluenzae*, mixed gram negative, *Enterobacter cloacae*, *Citrobacter freundii*, *Sphingomonas paucimobilis*, *Serratia marescens*. The following microorganisms are considered as fungal pathogens: *Candida albicans*, *Candida sp*, *Aspergillus*, *Aspergillus fumigatus*. **Figure S32.** A-B. Interaction between azithromycin and anti-staphylococcal prophylaxis on bacterial burden (A) and diversity (B) was evaluated through a two-way ANOVA. Only the effect of prophylaxis was statistically significant at *p*<0.05. **Figure S33.** A-B. Dot plots showing the number of 16S (A) and ITS2 (B) reads in BALF samples (blue), negative extraction controls (red) and bronchoscope washes (red). Groups were compared in the context of the Wilcoxon rank-sum test and p-values corrected using the false discovery rate method: ****, *p*<0.0001. C-D. Principal component analysis (PCA) plots showing the linear projection of the 16S- (C) and ITS2-based (D) compositional profiles onto a two-dimensional space. Each sampleis labelled based on the group membership: BALF specimens (blue circle), Negative extraction controls (orange triangle) and bronchoscope washes (grey cross). Ellipse plots defines 95% confidence region for the indicated sample groups. **Figure S34.** A. PCA plot showing the linear projection of the BALF samples (BALF, blue coloured) and the corresponding bronchoscope washes (wash, orange coloured) based on their bacterial compositional profiles. Paired BALF andbronchoscope washes are indicated with the same number. B. Procrustes analysis of the correlation between the bronchoscope washes and the paired BALF samples.Circles represent the spatial location of the BALF samples in the ordination to be rotated (PCA in BALF specimens), and blue arrows point to location of thepaired bronchoscope wash in the target ordination (PCA in bronchoscope washes). Solid black lines indicate the rotation of the indicated axes required to match samples in both ordinations as close as possible **Figure S35.** A-D. Procrustes analysis shows that the filtering steps did not remarkably impact the composition of the original bacterial (A-B) and fungal (C-D) profiles. Procrustes analyses of the correlation between the unfiltered dataset and the dataset without low counts, singletons and unclassified reads are shownin A and C. Plots in B and D, represent the degree of match between the microbial profiles in the original dataset and the dataset without low counts, singletons, unclassified reads and putative contaminants. Circles represent the spatial location of the samples in the first ordination (filtered dataset), and blue arrows point to location of the respective sample in the second ordination (unfiltered dataset). Solid black lines indicate the rotation of the indicated axes required to match samples in both ordinations as close as possible. **Figure S36.** A. Quantification of *Stenotrophomonas maltophilia* genetic material in the DNA extracts from the indicated sample type. B. Relationship between the absolute load of *Stenotrophomonas maltophilia* DNA using a specific qPCR assay, and the estimated abundance (relative abundance multiplied by the total bacterial load). The blue line and shaded areas represent the regression line and 95% confidence interval respectively. The good fit of the model suggests that the estimated burden constitutes a good approximation to the absolute load of *Stenotrophomonas maltophilia *(b 0.79,R^2^ 0.60, F(1,131)=198.10 *p*<0.0001)*.* **Figure S37.** Marginal effect of batch extraction on the odds of detecting bile acids with pointwise 95% confidence intervals, calculated from logistic regression models. Statistical significance was assessed using a Wald test for thedifference in log odds between each category and the reference group (Batch 1, B1). *, *p*<0.05; n.s., no significant. **Table S1.** Patient demographics. **Table S2.** Details about the study protocol deviation events. Asterisks indicate a patient who underwent two rounds with macrolides. **Table S3.** Bile acid profiles in BALF (µM). GDCA, glycodeoxycholic acid; GCDCA, glycochenodeoxycholic acid; GUDCA, glycoursodeoxycholic acid; TDCA, taurodeoxycholic acid; TCA, taurocholic acid; CA, cholic acid; TCDCA,taurochenodeoxycholic acid. **Table S4.** Correspondence between culture-based and molecular-based microbial profiles in BALF. Cultures identified as “mixed oral flora” are not included in this table, as these microorganisms are not identified to the species level. **Table S5.** Descriptive statistics for the bacterial load observed in negative extraction controls, bronchoscope washes and BALF specimens. The negative extraction controls were used as reference group when comparing the bacterial load between sample types. **Table S6.** This table contains information in regards to the relative abundance (mean and standard deviation) and prevalence of the top 11 fungal ASVs obtained from the BALF DNA extracts. The profile contains fungi associated with the nasal (*Aureobasidium pullulans*, *Alternaria*, *Candida albicans*) and oral (*Malassezia*, *Alternaria*, *Candida*,* Aspergillus*, *Filobasidium*,* Aureobasidium* and *Cryptococcus *species) cavities [3–5], skin commensals (*Malassezia restricta*, *Malassezia globosa*, *Candida parasilopsis*, *Alternaria*, *Rhodotorula*, *Cryptococcus*, *Aspergillus*) [6], and fungi described in the respiratory tract of CF patients (*Malassezia restricta*, *Malassezia globosa* and *Candida albicans*) [7, 8]. Interestingly, many of the observed species in the fungal profile have been also described at high prevalence in the indoor environment, and have been proposed to colonize the lungs through environmental exposure [9]. *,Formerly known as *Cryptococcus albidus*;**, *Meyerozyma guilliermondii* is the teleomorph specie of *Candida guilliermondii*. **Table S7.** Absolute read counts for the OTUs detected as contaminants by the Rpackage *decontam* [10]. BALF specimens are identified by an alphanumeric code in which the three first letters identify the Centre (PER, BRC, BMC, NZA, SWC, SSC, MRC, MMC, ADL, LCC). Negative extraction controls are identified with “CNT”, while bronchoscope washes are named with “WASH” followed by the code of the paired BALF specimen. **Table S8.** Results of the permutational test of significance of the Procrustes analyses (using the function *protest *implemented in the R package *vegan* [11]). The great concordance shown between the compared datasets (low M^2^ and high correlation coefficient) suggests that the filtering steps did not remarkably impact the composition of the original microbial profiles (Rawcounts). **Table S9.** Absolute read counts for the ASVs detected as contaminants by the Rpackage *decontam* in the ITS2-based fungal profiles [10]. BALF specimens are identified by an alphanumeric code in which the three first letters identify the Centre (BRC, BMC, NZA, SWC, SSC, MRC, LCC). Negative extraction controls are identified with “CNT”, while bronchoscope washes are named with “WASH” followed by the code of the paired BALF specimen. For simplification ASVs are indicated with numbers and identified in Supplemental Table 9. **Table S10.** Biological classification of the ASVs identified as potential contaminants shown in Supplemental Table 8. Eleven different ASVs were assigned to the *Malassezia restricta* species rank, of which one was identified as potential contaminants. Anagously, eight ASVs were classified as *Aspergillus*, being two of them was predicted as a putative contaminant. **Table S11.** Results of the permutational test of significance of the Procrustes analyses (using the function *protest *implemented in the R package *vegan* [11]). The great concordance shown between the compared datasets (low M^2^ and high correlation coefficient) suggests that the filtering steps did not remarkably impact the composition of the original microbial profiles (Raw counts). **Table S12.** Technical information related to the TaqMan® assay for *Malassezia restricta.* **Table S13.** System information and R packages used in this study.

## Data Availability

All the sequencing data generated in this study have been deposited in the Sequencing Read Archive (BIOPROJECT PRJNA819196).

## References

[1] Elborn JS (2016). Cystic fibrosis. Lancet.

[2] Ranganathan SC, Hall GL, Sly PD, Stick SM, Douglas TA, Australian Respiratory Early Surveillance Team for Cystic F (2017). Early lung disease in infants and preschool children with cystic fibrosis. What have we learned and what should we do about it?. Am J Respir Crit Care Med.

[3] Sly PD, Gangell CL, Chen L, Ware RS, Ranganathan S, Mott LS (2013). Risk factors for bronchiectasis in children with cystic fibrosis. N Engl J Med.

[4] Esther CR, Muhlebach MS, Ehre C, Hill DB, Wolfgang MC, Kesimer M (2019). Mucus accumulation in the lungs precedes structural changes and infection in children with cystic fibrosis. Sci Transl Med.

[5] Montgomery ST, Mall MA, Kicic A, Stick SM, Arest CF (2017). Hypoxia and sterile inflammation in cystic fibrosis airways: mechanisms and potential therapies. Eur Respir J.

[6] Zemanick ET, Wagner BD, Robertson CE, Ahrens RC, Chmiel JF, Clancy JP (2017). Airway microbiota across age and disease spectrum in cystic fibrosis. Eur Respir J.

[7] Tirouvanziam R, Gernez Y, Conrad CK, Moss RB, Schrijver I, Dunn CE (2008). Profound functional and signaling changes in viable inflammatory neutrophils homing to cystic fibrosis airways. Proc Natl Acad Sci U S A.

[8] Cuthbertson L, Walker AW, Oliver AE, Rogers GB, Rivett DW, Hampton TH (2020). Lung function and microbiota diversity in cystic fibrosis. Microbiome.

[9] Khan TZ, Wagener JS, Bost T, Martinez J, Accurso FJ, Riches DW (1995). Early pulmonary inflammation in infants with cystic fibrosis. Am J Respir Crit Care Med.

[10] Rosenow T, Mok LC, Turkovic L, Berry LJ, Sly PD, Ranganathan S (2019). The cumulative effect of inflammation and infection on structural lung disease in early cystic fibrosis. Eur Respir J.

[11] Margaroli C, Garratt LW, Horati H, Dittrich AS, Rosenow T, Montgomery ST (2019). Elastase exocytosis by airway neutrophils is associated with early lung damage in children with cystic fibrosis. Am J Respir Crit Care Med.

[12] Caparros-Martin JA, Flynn S, Reen FJ, Woods DF, Agudelo-Romero P, Ranganathan SC (2020). The detection of bile acids in the lungs of paediatric cystic fibrosis patients is associated with altered inflammatory patterns. Diagnostics (Basel).

[13] Flynn S, Reen FJ, Caparros-Martin JA, Woods DF, Peplies J, Ranganathan SC (2020). Bile acid signal molecules associate temporally with respiratory inflammation and microbiome signatures in clinically stable cystic fibrosis patients. Microorganisms.

[14] Wahlstrom A, Sayin SI, Marschall HU, Backhed F (2016). Intestinal crosstalk between bile acids and microbiota and its impact on host metabolism. Cell Metab.

[15] Price CE, O’Toole GA (2021). The gut-lung axis in cystic fibrosis. J Bacteriol.

[16] Stick SM, Foti A, Ware RS, Tiddens H, Clements BS, Armstrong DS, et al. The effect of azithromycin on structural lung disease in infants with cystic fibrosis (COMBAT CF): a phase 3, randomised, double-blind, placebo-controlled clinical trial. Lancet Respir Med. 2022;10(8):776-84. 10.1016/S2213-2600(22)00165-5. 10.1016/S2213-2600(22)00165-5 35662406

[17] Broad J, Sanger GJ (2013). The antibiotic azithromycin is a motilin receptor agonist in human stomach: comparison with erythromycin. Br J Pharmacol.

[18] Rosenow T, Oudraad MC, Murray CP, Turkovic L, Kuo W, de Bruijne M (2015). PRAGMA-CF. A quantitative structural lung disease computed tomography outcome in young children with cystic fibrosis. Am J Respir Crit Care Med.

[19] Buzina W, Braun H, Freudenschuss K, Lackner A, Habermann W, Stammberger H (2003). Fungal biodiversity–as found in nasal mucus. Med Mycol.

[20] Willger SD, Grim SL, Dolben EL, Shipunova A, Hampton TH, Morrison HG (2014). Characterization and quantification of the fungal microbiome in serial samples from individuals with cystic fibrosis. Microbiome.

[21] Findley K, Oh J, Yang J, Conlan S, Deming C, Meyer JA (2013). Topographic diversity of fungal and bacterial communities in human skin. Nature.

[22] Boix-Amoros A, Martinez-Costa C, Querol A, Collado MC, Mira A (2017). Multiple approaches detect the presence of fungi in human breastmilk samples from healthy mothers. Sci Rep.

[23] Dupuy AK, David MS, Li L, Heider TN, Peterson JD, Montano EA (2014). Redefining the human oral mycobiome with improved practices in amplicon-based taxonomy: discovery of Malassezia as a prominent commensal. PLoS One.

[24] Limon JJ, Skalski JH, Underhill DM (2017). Commensal fungi in health and disease. Cell Host Microbe.

[25] Holmes I, Harris K, Quince C (2012). Dirichlet multinomial mixtures: generative models for microbial metagenomics. PLoS One.

[26] Rohof WO, Bennink RJ, de Ruigh AA, Hirsch DP, Zwinderman AH, Boeckxstaens GE (2012). Effect of azithromycin on acid reflux, hiatus hernia and proximal acid pocket in the postprandial period. Gut.

[27] Larson JM, Tavakkoli A, Drane WE, Toskes PP, Moshiree B (2010). Advantages of azithromycin over erythromycin in improving the gastric emptying half-time in adult patients with gastroparesis. J Neurogastroenterol Motil.

[28] Padda MS, Sanchez M, Akhtar AJ, Boyer JL (2011). Drug-induced cholestasis. Hepatology.

[29] Rosen R, Lurie M, Kane M, DiFilippo C, Cohen A, Freiberger D (2021). Risk factors for bile aspiration and its impact on clinical outcomes. Clin Transl Gastroenterol.

[30] Henen S, Denton C, Teckman J, Borowitz D, Patel D (2021). Review of gastrointestinal motility in cystic fibrosis. J Cyst Fibros.

[31] Ishak A, Stick SM, Turkovic L, Ranganathan SC, King L, Harrison J (2020). BAL inflammatory markers can predict pulmonary exacerbations in children with cystic fibrosis. Chest.

[32] Dickson RP, Martinez FJ, Huffnagle GB (2014). The role of the microbiome in exacerbations of chronic lung diseases. Lancet.

[33] Wu BG, Sulaiman I, Tsay JJ, Perez L, Franca B, Li Y (2021). Episodic aspiration with oral commensals induces a MyD88-dependent, pulmonary T-Helper cell type 17 response that mitigates susceptibility to streptococcus pneumoniae. Am J Respir Crit Care Med.

[34] Dickson RP, Erb-Downward JR, Freeman CM, McCloskey L, Falkowski NR, Huffnagle GB (2017). Bacterial topography of the healthy human lower respiratory tract. mBio.

[35] Jorth P, Ehsan Z, Rezayat A, Caldwell E, Pope C, Brewington JJ (2019). Direct lung sampling indicates that established pathogens dominate early infections in children with cystic fibrosis. Cell Rep.

[36] Pittman JE, Wylie KM, Akers K, Storch GA, Hatch J, Quante J (2017). Association of antibiotics, airway microbiome, and inflammation in infants with cystic fibrosis. Ann Am Thorac Soc.

[37] Acosta N, Thornton CS, Surette MG, Somayaji R, Rossi L, Rabin HR (2021). Azithromycin and the microbiota of cystic fibrosis sputum. BMC Microbiol.

[38] Mac Aogain M, Lau KJX, Cai Z, Kumar Narayana J, Purbojati RW, Drautz-Moses DI (2020). Metagenomics reveals a core macrolide resistome related to microbiota in chronic respiratory disease. Am J Respir Crit Care Med.

[39] Saladie M, Caparros-Martin JA, Agudelo-Romero P, Wark PAB, Stick S, O'Gara F (2020). Microbiomic analysis on low abundant respiratory biomass samples; improved recovery of microbial DNA from bronchoalveolar lavage fluid. Front Microbiol.

[40] Klindworth A, Pruesse E, Schweer T, Peplies J, Quast C, Horn M (2013). Evaluation of general 16S ribosomal RNA gene PCR primers for classical and next-generation sequencing-based diversity studies. Nucleic Acids Res.

[41] Quast C, Pruesse E, Yilmaz P, Gerken J, Schweer T, Yarza P (2013). The SILVA ribosomal RNA gene database project: improved data processing and web-based tools. Nucleic Acids Res.

[42] Pruesse E, Peplies J, Glockner FO (2012). SINA: accurate high-throughput multiple sequence alignment of ribosomal RNA genes. Bioinformatics.

[43] Rognes T, Flouri T, Nichols B, Quince C, Mahe F (2016). VSEARCH: a versatile open source tool for metagenomics. Peer J.

[44] Callahan BJ, McMurdie PJ, Rosen MJ, Han AW, Johnson AJA, Holmes SP (2016). DADA2: high-resolution sample inference from Illumina amplicon data. Nat Methods.

[45] Abarenkov K, Zirk A, Piirmann T, Pöhönen R, Ivanov F, Nilsson RH, et al. UNITE general FASTA release for Fungi. UNITE Community. 2020. 10.15156/BIO/786368.

[46] Wang Q, Garrity GM, Tiedje JM, Cole JR (2007). Naive Bayesian classifier for rapid assignment of rRNA sequences into the new bacterial taxonomy. Appl Environ Microbiol.

[47] Marsh RL, Nelson MT, Pope CE, Leach AJ, Hoffman LR, Chang AB (2018). How low can we go? The implications of low bacterial load in respiratory microbiota studies. Pneumonia (Nathan).

[48] Eisenhofer R, Minich JJ, Marotz C, Cooper A, Knight R, Weyrich LS (2019). Contamination in low microbial biomass microbiome studies: issues and recommendations. Trends Microbiol.

[49] Ilahi A, Hadrich I, Neji S, Trabelsi H, Makni F, Ayadi A (2017). Real-time PCR identification of six malassezia species. Curr Microbiol.

[50] Caparros-Martin JA, Lareu RR, Ramsay JP, Peplies J, Reen FJ, Headlam HA (2017). Statin therapy causes gut dysbiosis in mice through a PXR-dependent mechanism. Microbiome.

[51] Lin H, Peddada SD (2020). Analysis of compositions of microbiomes with bias correction. Nat Commun.

