# Supplementary material for: Detection of bile acids in bronchoalveolar lavage fluid defines the inflammatory and microbial landscape of the lower airways in infants with cystic fibrosis
Source: Microbiome. 2023 Jun 13;11:132. doi: 10.1186/s40168-023-01543-9 (PMC10262387; doi:10.1186/s40168-023-01543-9)
Supplement: Supplementary file 2 — Additional file 1: Figure S1. Related to Figure 1. Sensitivity analysis controlling for potential batch effects in bile acid detection. See material and methods and Figure S37. A-B.Box plots overlaid with density curves (violin plots, blue) representing the proportion neutrophils (A) and the levels of IL8 (B) with respect to the detection of BAs in BALF. Individual data points (red) with jitter are represented on the top of each box plot. Groups were compared using the Wilcoxon rank-sum test: ****, p<0.0001. Figure S2. Box plot overlaid with density curves (violin plots, blue) representing Neutrophil elastase (NE) activity with respect to the detection of BAs in BALF. Individual data points (red) with jitter are represented on the top of eachplot. Figure S3. Related to Figure 1C-H and Figure S4. Sensitivity analyses controlling for potential batch effects. See material and methods and Figure S37. A-F. Boxplots overlaid with density curves (violin plots, blue) representing the proportion of the lung with structural disease (A), the number of pulmonary exacerbations (B), the number of days hospitalised in the case of a pulmonary exacerbation (C), the number of oral/inhaled antibiotic rounds (D), theaveraged number of days (Log10 transformed) treated with oral/inhaled antibiotics (E), and the number of intravenous antibiotics rounds (F), during the first year of life with respect to the detection of BAs in BALF. Individual data points (red) with jitter are represented on the top of each box plot. The proportion of the lung with structural damage in A was determined from CT scans using the PRAGMA scoring system [1]. The data represented in E includes the total duration of any treatment started before the collection of the BALF samples. Groups were compared using the Wilcoxon rank-sum test: ***, p<0.001; **, p<0.01; *, p<0.05; n.s., no significant (p>0.05). Figure S4. A-B. Box plots overlaid with density curves (violin plots, blue) representing the number of days hospita [file 40168_2023_1543_MOESM1_ESM.docx]

**DETECTION OF BILE ACIDS IN BRONCHOALVEOLAR LAVAGE FLUID DEFINES THE INFLAMMATORY AND MICROBIAL LANDSCAPE OF THE LOWER AIRWAYS IN INFANTS WITH CYSTIC FIBROSIS.**

**SUPPLEMENTAL MATERIAL**

Jose A. Caparrós-Martín, Montserrat Saladie, S. Patricia Agudelo-Romero, F. Jerry Reen, Robert S. Ware, Peter D. Sly, Stephen M. Stick and Fergal O’Gara on behalf of the COMBAT study group.


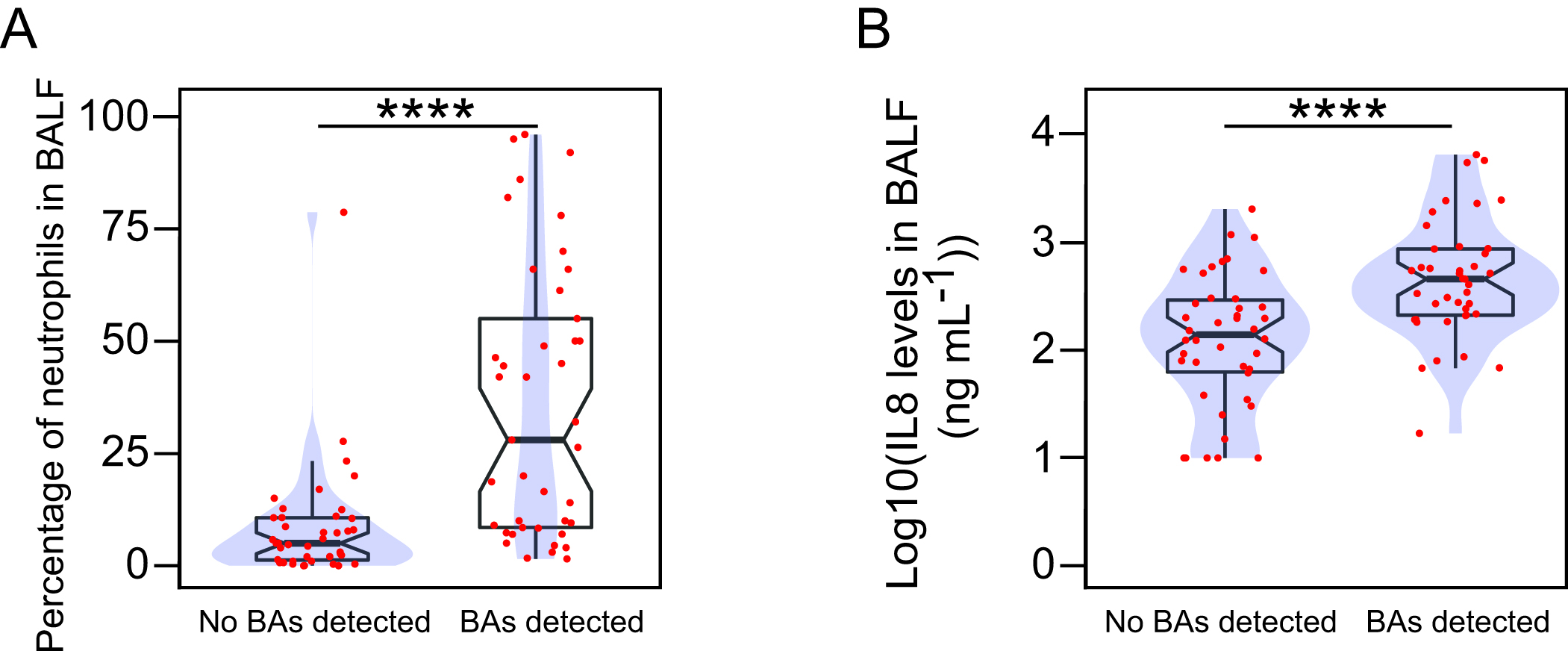


**Figure S1.** Related to Figure 1. Sensitivity analysis controlling for potential batch effects in bile acid detection. See material and methods and Figure S37. **A-B.** Box plots overlaid with density curves (violin plots, blue) representing the proportion neutrophils (**A**) and the levels of IL8 (**B**) with respect to the detection of BAs in BALF. Individual data points (red) with jitter are represented on the top of each box plot. Groups were compared using the Wilcoxon rank-sum test: ****, *p*<0.0001.

**
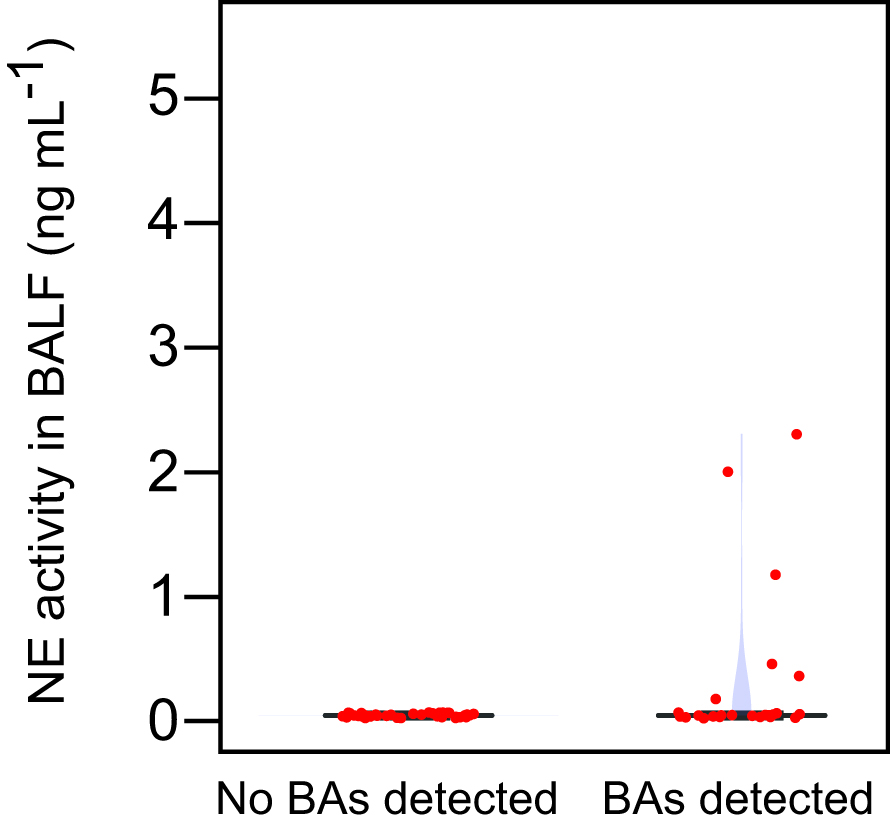
**

**Figure S2.** Box plot overlaid with density curves (violin plots, blue) representing Neutrophil elastase (NE) activity with respect to the detection of BAs in BALF. Individual data points (red) with jitter are represented on the top of each plot.


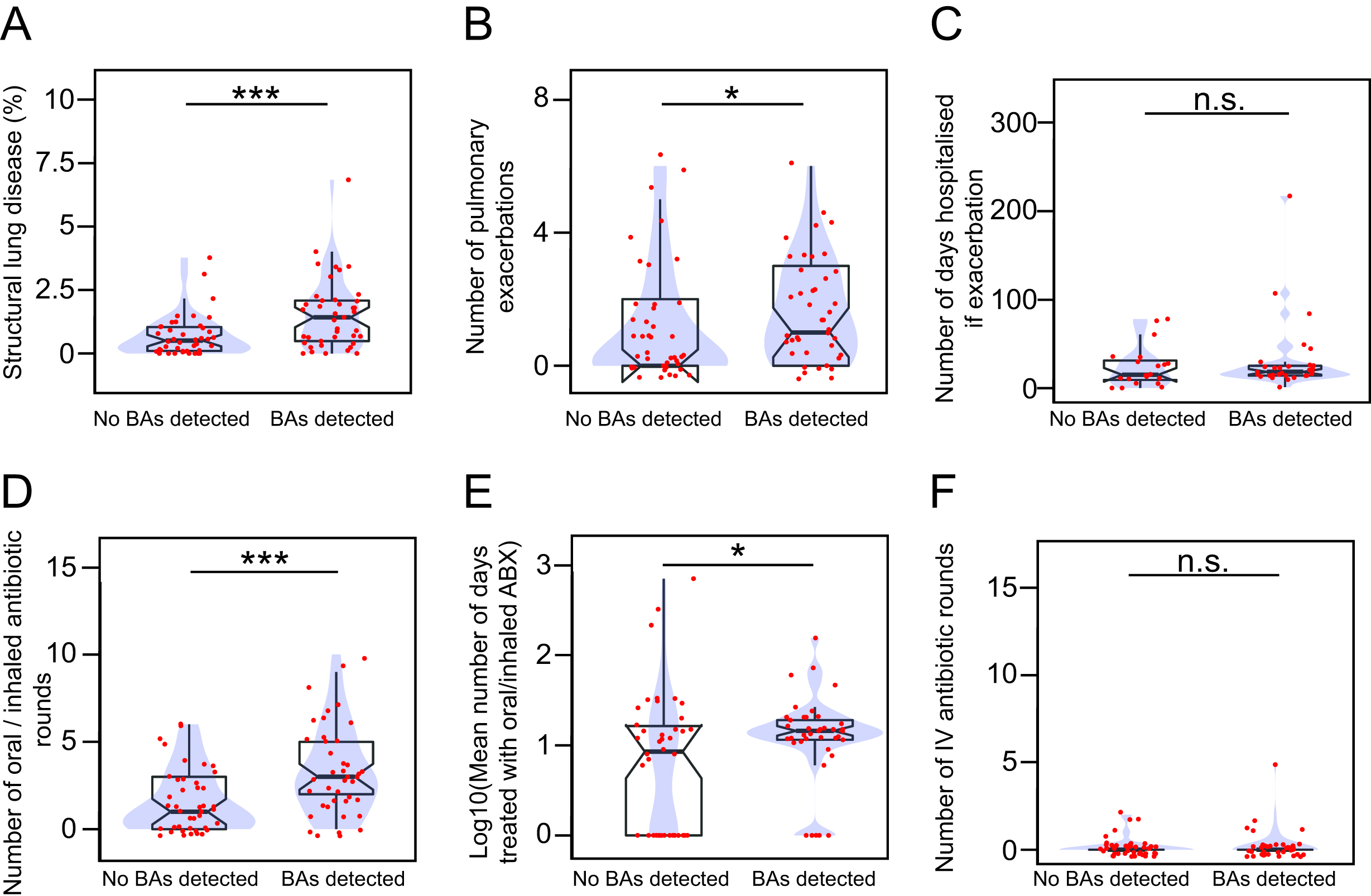


**Figure S3.** Related to Figure 1C-H and Figure S4. Sensitivity analyses controlling for potential batch effects. See material and methods and Figure S37. **A-F.** Box plots overlaid with density curves (violin plots, blue) representing the proportion of the lung with structural disease (**A**), the number of pulmonary exacerbations (**B**), the number of days hospitalised in the case of a pulmonary exacerbation (**C**), the number of oral/inhaled antibiotic rounds (**D**), the averaged number of days (Log10 transformed) treated with oral/inhaled antibiotics (**E**), and the number of intravenous antibiotics rounds (**F**), during the first year of life with respect to the detection of BAs in BALF. Individual data points (red) with jitter are represented on the top of each box plot. The proportion of the lung with structural damage in **A** was determined from CT scans using the PRAGMA scoring system [[1](#_ENREF_1)]. The data represented in **E** includes the total duration of any treatment started before the collection of the BALF samples. Groups were compared using the Wilcoxon rank-sum test: ***, *p*<0.001; **, *p*<0.01; *, *p*<0.05; n.s., no significant (*p*>0.05).


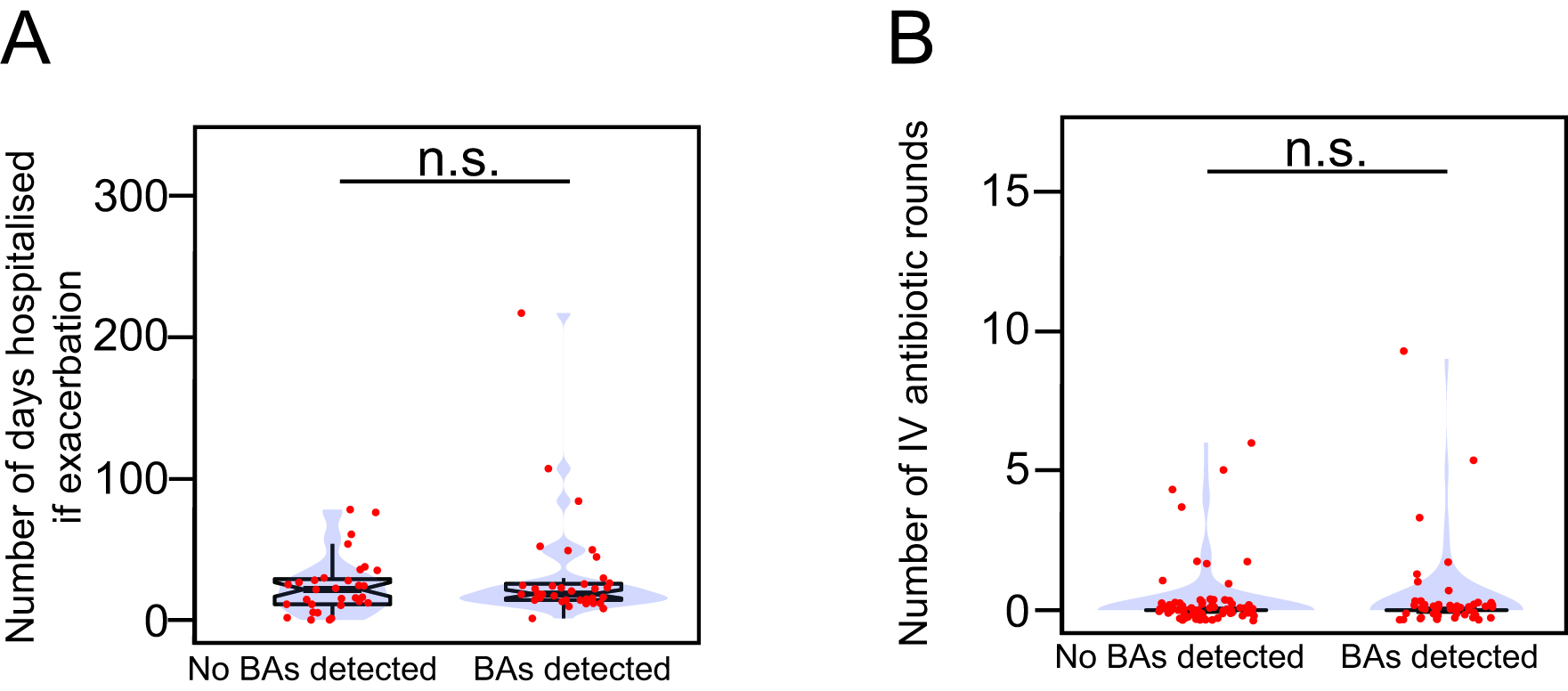


**Figure S4.** **A-B.** Box plots overlaid with density curves (violin plots, blue) representing the number of days hospitalised in the case of a pulmonary exacerbation (**A**), and the number of intravenous antibiotics rounds (**B**), during the first year of life with respect to the detection of BAs in BALF. Individual data points (red) with jitter are represented on the top of each box plot. Groups were compared using the Wilcoxon rank-sum test: n.s., no significant (*p*>0.05).


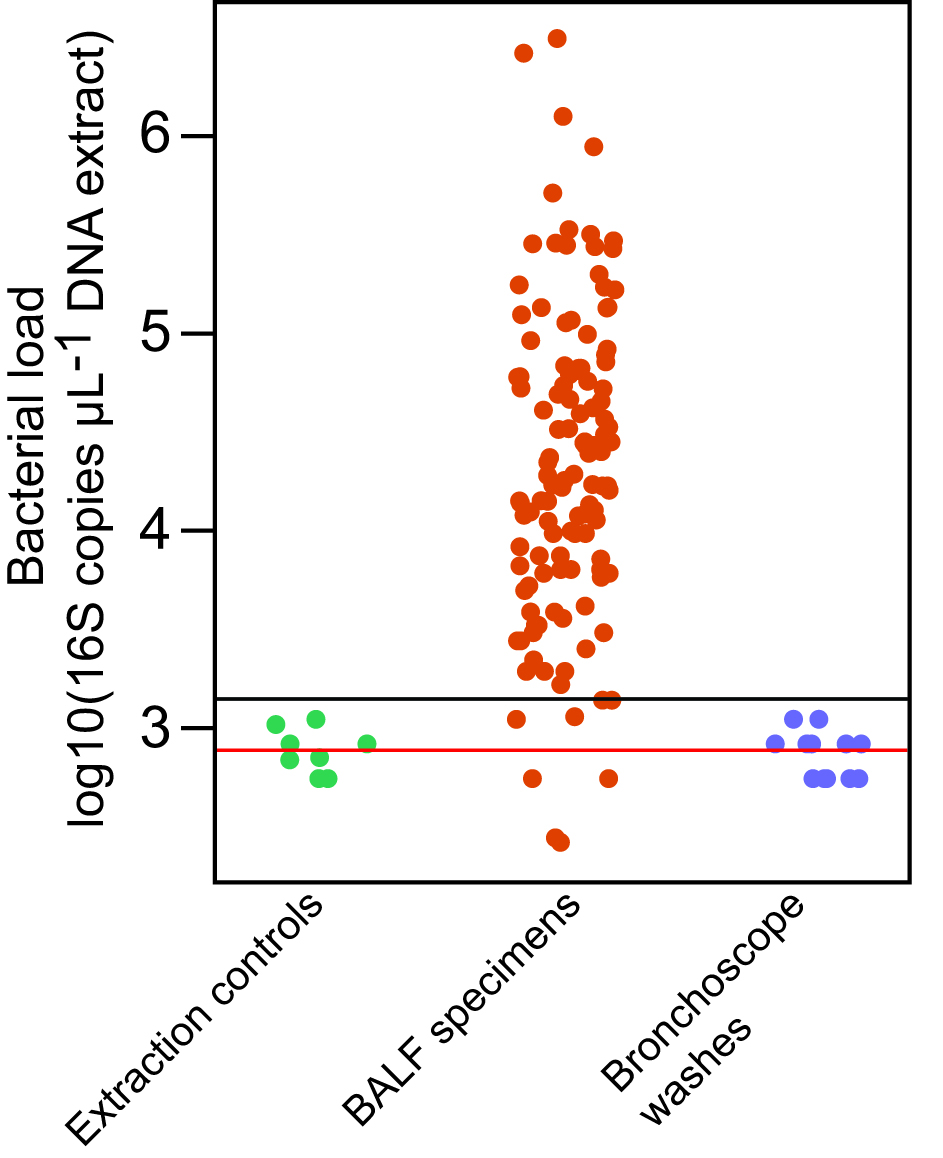


**Figure S5.** The dot plot shows the quantification by qPCR of the bacterial burden in the DNA extracts from the indicated sample type. The mean bacterial load in the controls (including bronchoscope washes and negative extraction controls) is indicated with a horizontal red line. The horizontal black line lays 3 standard deviations from the mean bacterial load in the controls (red line), and it was set as the lower limit of detection in our cohort.


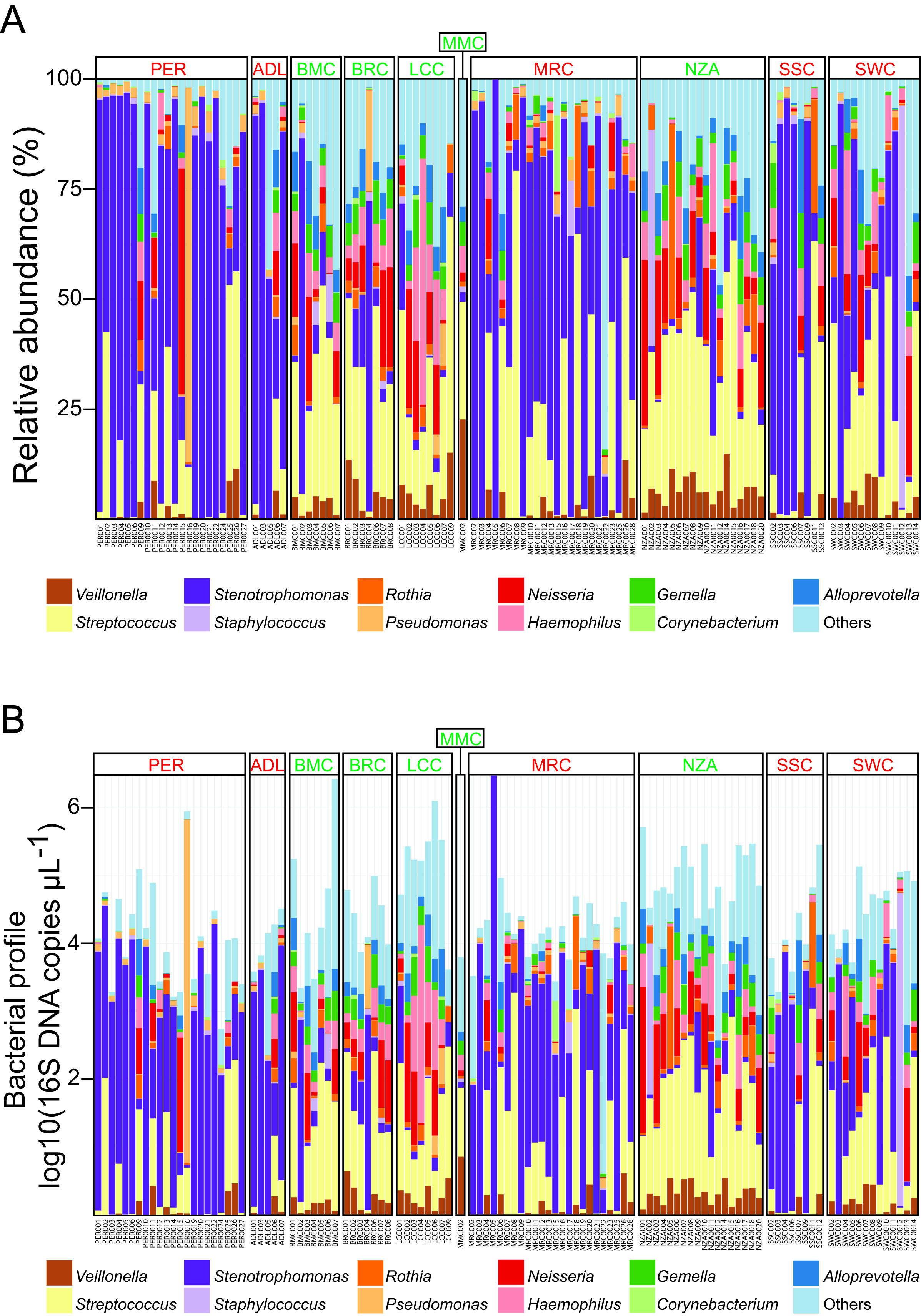


**Figure S6.** **A-B.** Bar plots representing the bacterial compositional profiles of the BALF samples as relative proportions (**A**), or as normalized levels (relative proportions multiplied by the total bacterial load of each sample) (**B**). Only the top 11 OTUs are represented. Specimens are grouped per collection Centre. Centres performing anti-*Staphylococcus* prophylaxis are labelled in red.


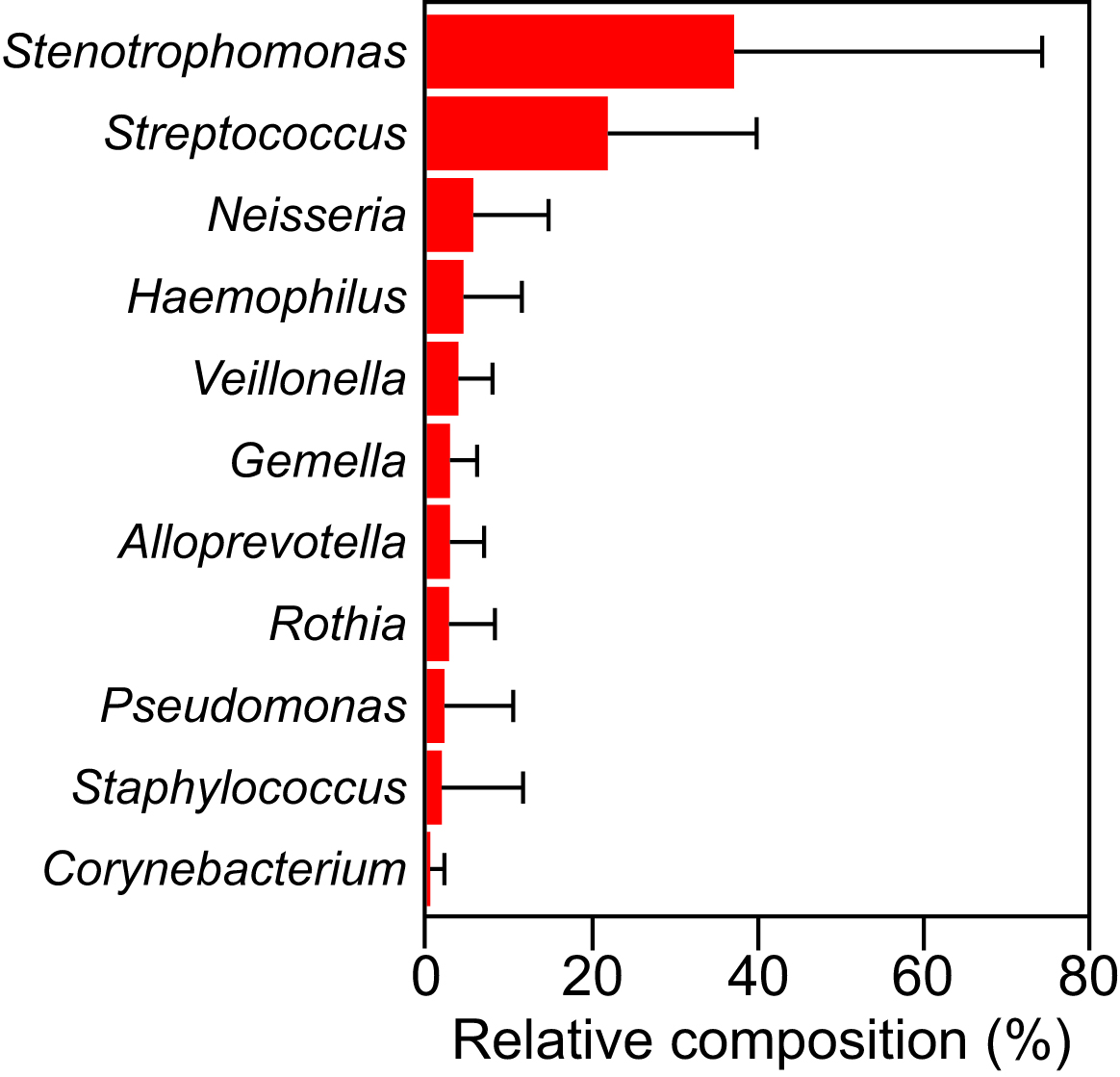


**Figure S7.** Barplot represents the mean and standard deviation for the top 11 bacterial OTUs at genus level resolution.


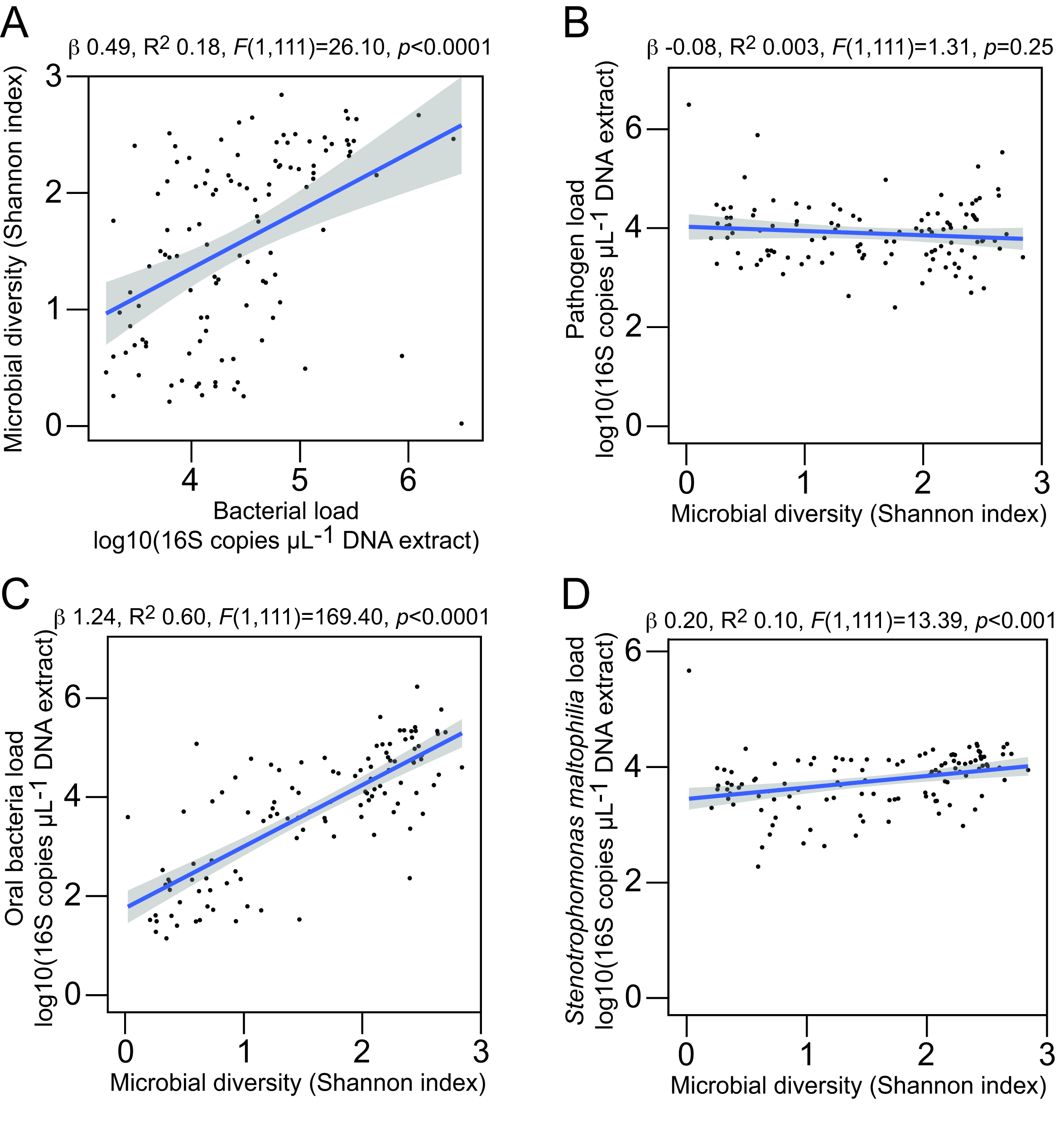


**Figure S8.** **A-E**. Linear relationship between microbial diversity (Shannon index) and total bacterial burden (**A**), and estimated pathogen (**B**) or oral bacteria (**C**) load in bacterial DNA extracts from BALF. **D.** Dot plot showing the association between microbial diversity and absolute abundance of *Stenotrophomonas maltophilia* in BALF. Line of best fit for each linear model is plotted with 95% confidence interval. Estimated absolute abundance in (**B**) and (**C**) was calculated by multiplying the relative abundance of each bacterial group by the total bacterial load obtained through qPCR. We considered the following OTUs as CF pathogens (*Stenotrophomonas*, *Haemophilus*, *Staphylococcus*, *Pseudomonas*, *Bordetella*, *Escherichia-Shigella*) [[2](#_ENREF_2)]. Oral bacteria group represents the following taxonomic entities (*Streptococcus*, *Neisseria*, *Veillonella*, *Alloprevotella*, *Gemella*, *Rothia*, *Porphyromonas*, *Granulicatella*, *Prevotella_7*, *Leptotrichia*), which were found at least at 1% relative abundance in our dataset. The regression coefficient, coefficient of determination, and the results of the F-test for each model are shown on the top of each plot.

**
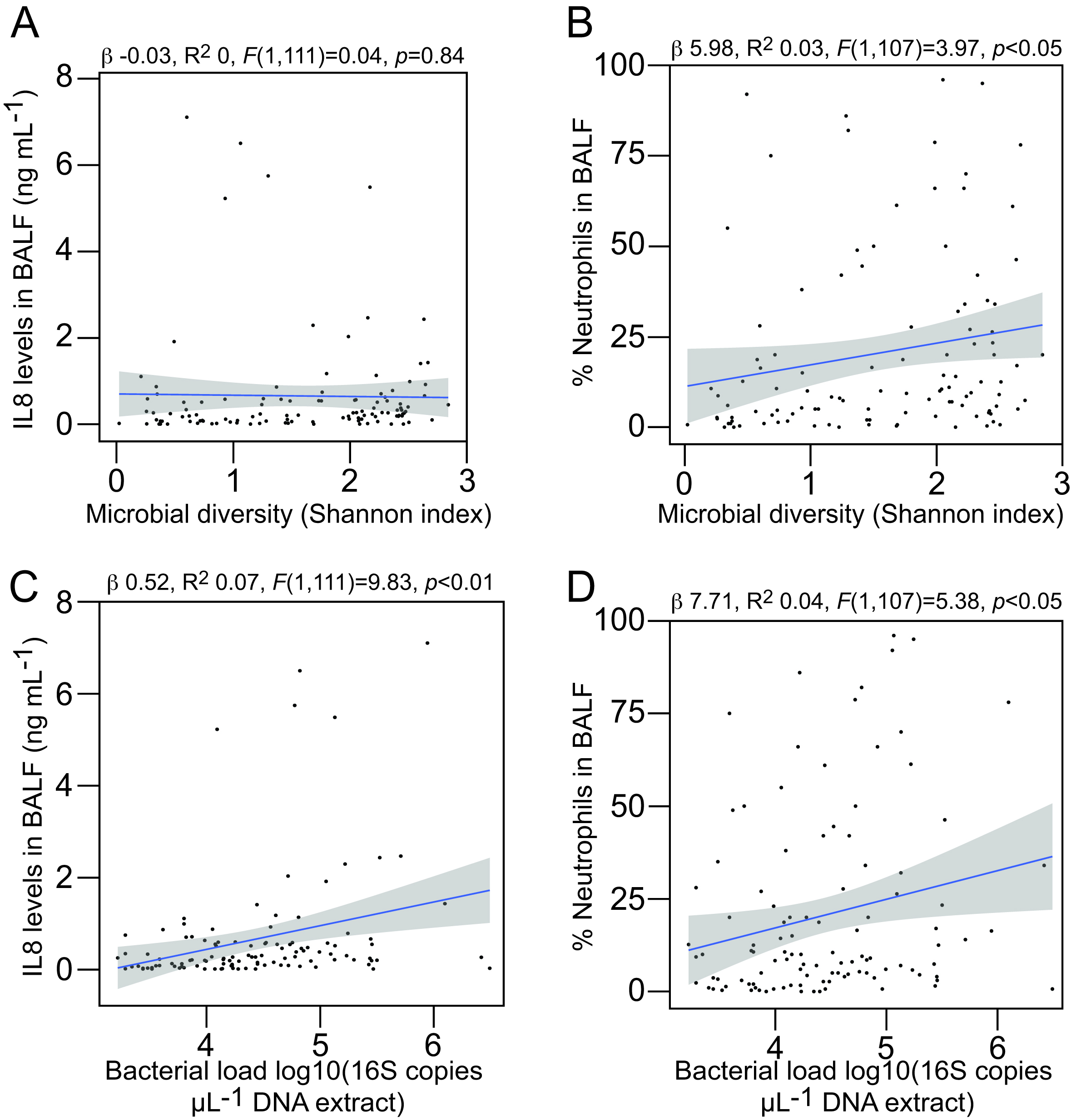
**

**Figure S9.** **A-D**. Dot plots representing the relationship between inflammatory markers and microbial diversity (**A-B**) or bacterial biomass (**C-D**) in BALF. For each linear model, we have plotted the best-fit regression line with 95% confidence interval. The regression coefficient, coefficient of determination, and the results of the F-test for each model are shown on the top of each plot.


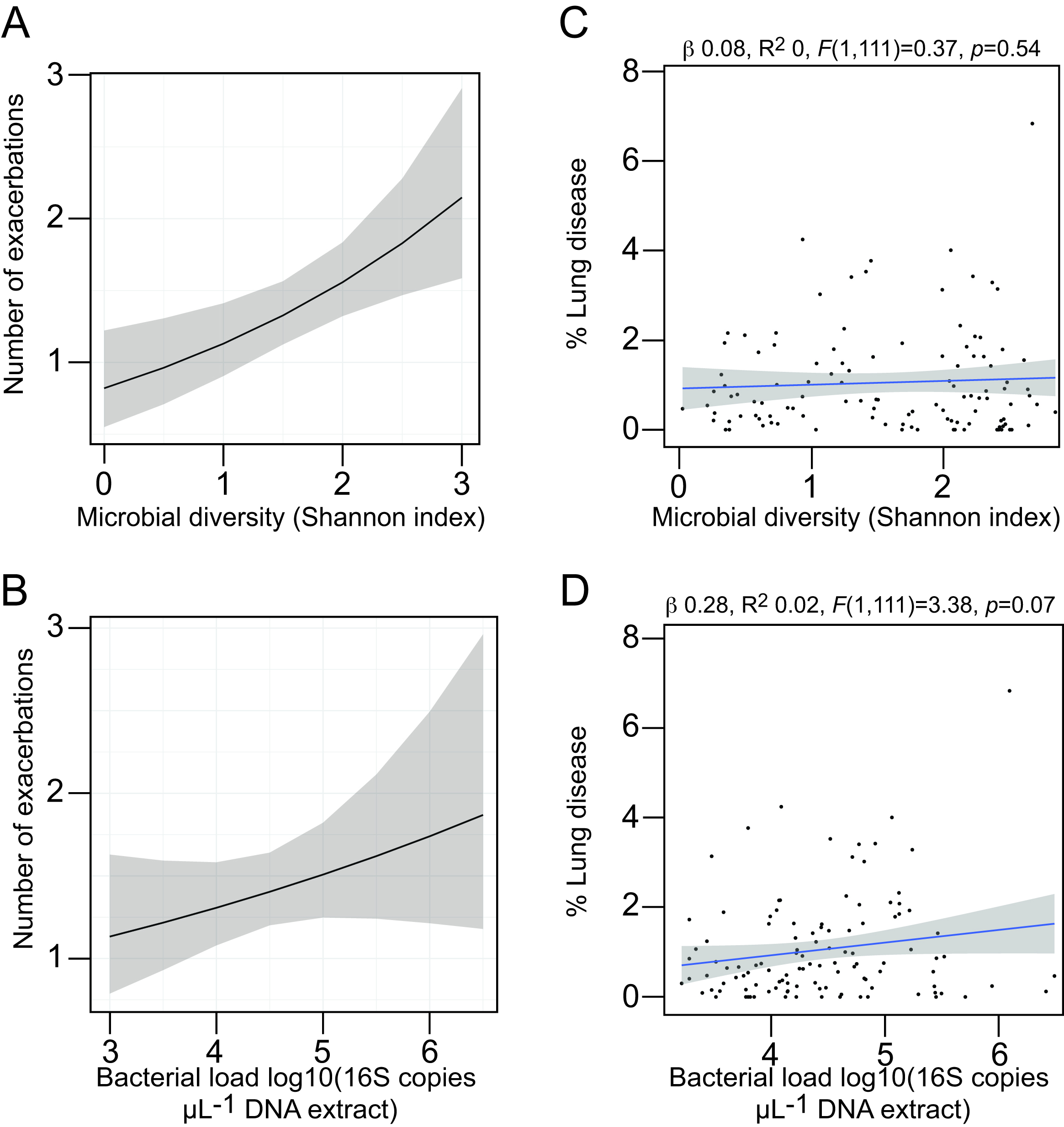


**Figure S10.** **A-B**. Marginal effect of bacterial diversity (**A**) and bacterial burden (**B**) in BALF on the number of exacerbations with pointwise 95% confidence intervals, calculated from a Poisson regression model. Accordingly with the model in **A**, for one unit increase in diversity there was 1.37 (95% confidence interval 1.12-1.70, *p*=0.003) times more exacerbations. We did not observe a relationship between the number of exacerbations during the first year of life and the bacterial load (number of exacerbations per unit increase in bacterial load 1.15, 95% confidence interval 0.93-1.43, *p*=0.20). **C-D**. Dot plots representing the relationship between percentage of structural lung disease and microbial diversity (**C**) or bacterial biomass (**D**) in BALF. For each linear model, we have plotted the best-fit regression line with 95% confidence interval. The regression coefficient, coefficient of determination, and the results of the F-test for each model are shown on the top of each plot.


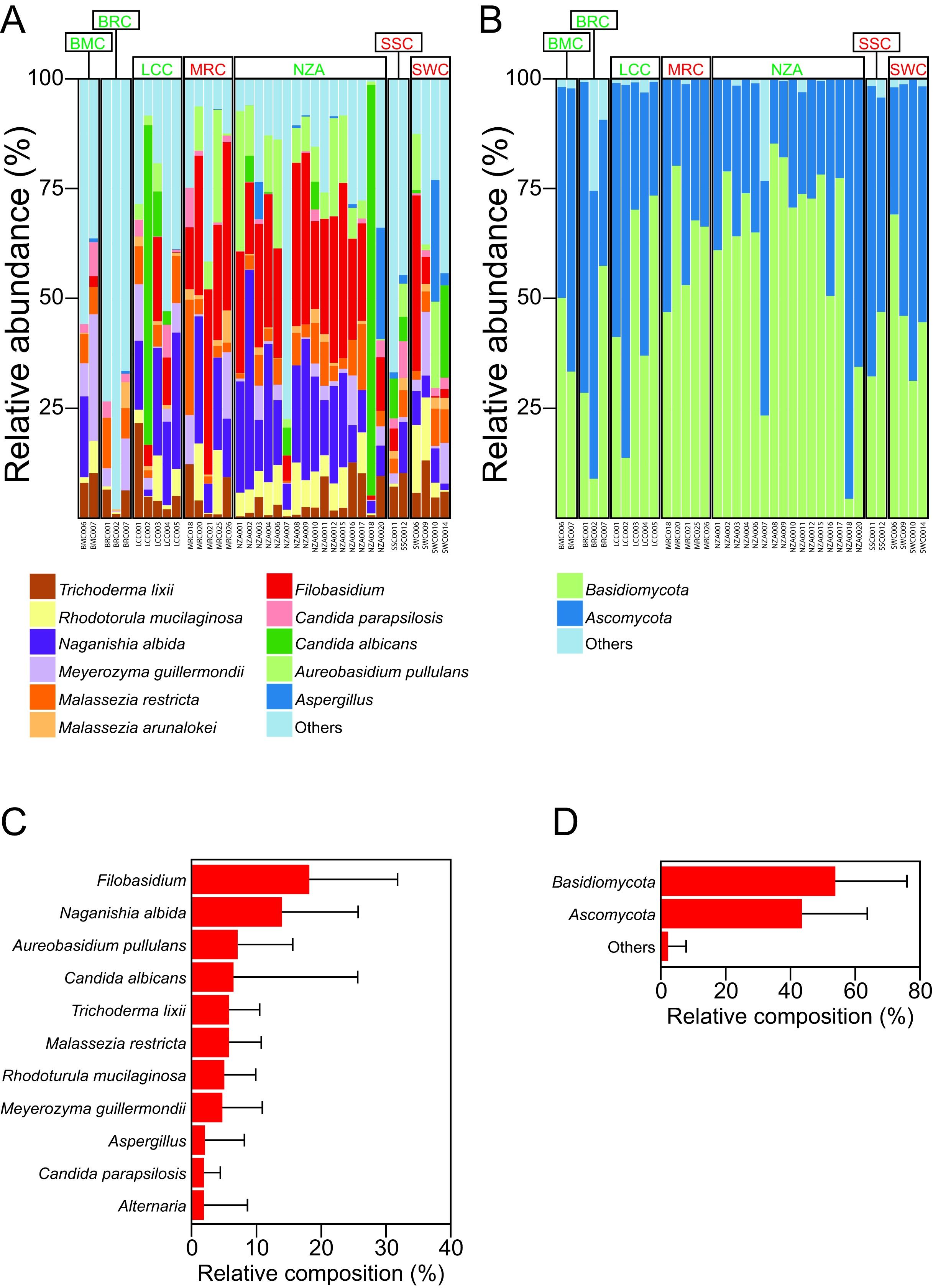


**Figure S11.** **A-B.** Bar plots representing the fungal compositional profiles of the BALF samples as relative proportions at specie (**A**), and at phylum levels (**B**). Only the top 11 ASVs are represented. Specimens are grouped per collection centre. Centres performing anti-*Staphylococcus* prophylaxis are labelled in red. **C-D.** Barplots represent the mean and standard deviation for the top 11 ASVs at species level resolution (**C**), or for the top 2 phyla (**D**).

**
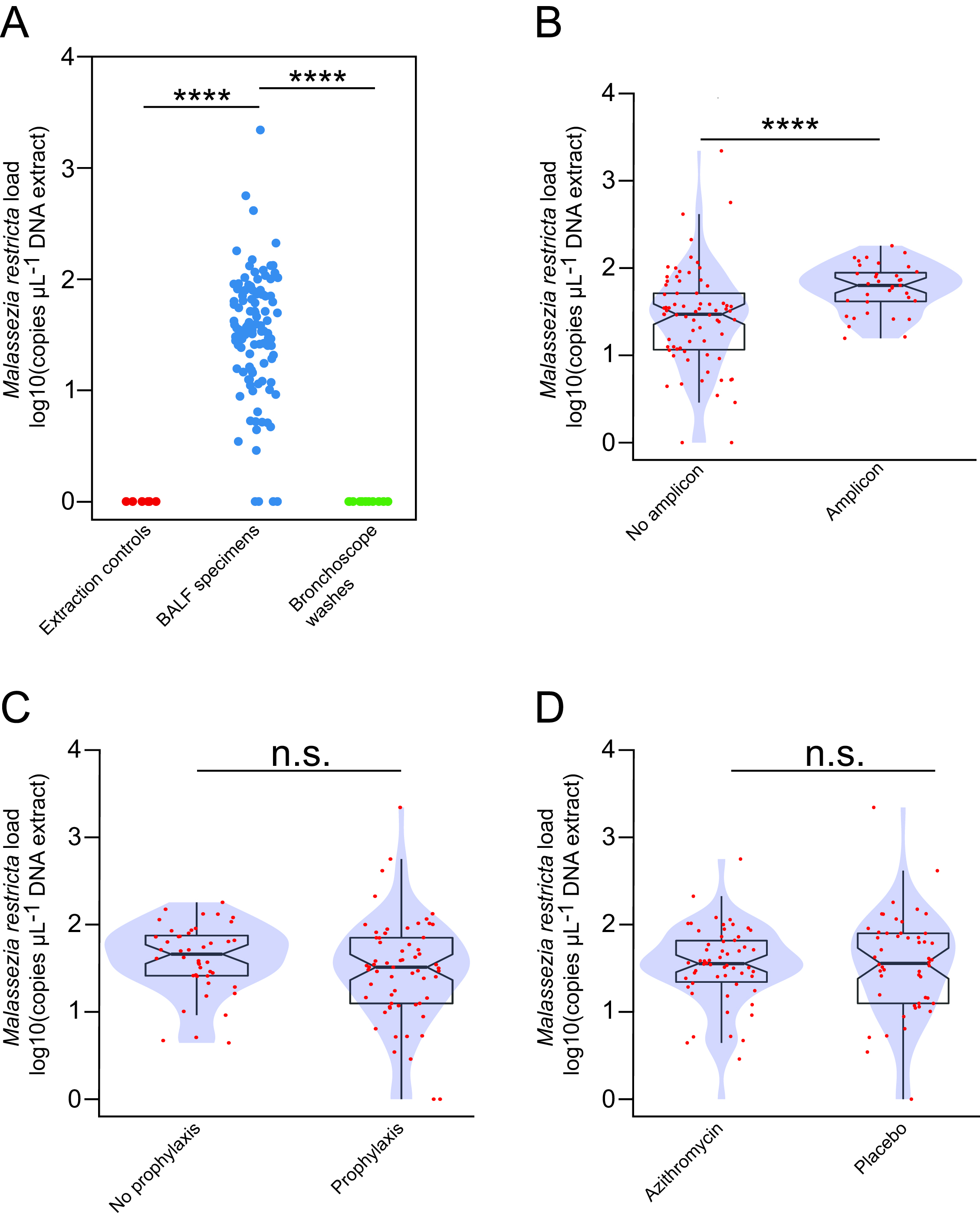
**

**Figure S12.** **A.** Quantification of *Malassezia restricta* genetic material in the DNA extracts from the indicated sample type. **C-D.** Box plots overlaid with density curves (violin plots, blue) representing the load of *Malassezia restricta* in BALF with respect to the samples that yielded ITS2 library amplification (**B**), the anti-staphylococcal regimen at the time of BALF collection (**C**), or the treatment arm (azithromycin or placebo) (**D**). Individual data points (red) with jitter are represented on the top of each box plot. Notches in the boxplot represent 95% confidence interval for the median. Groups were compared using the Wilcoxon rank-sum test: ****, *p*<0.0001; n.s., no significant (*p*>0.05). Conditioned probabilities in **A** were corrected using the false discovery rate method.


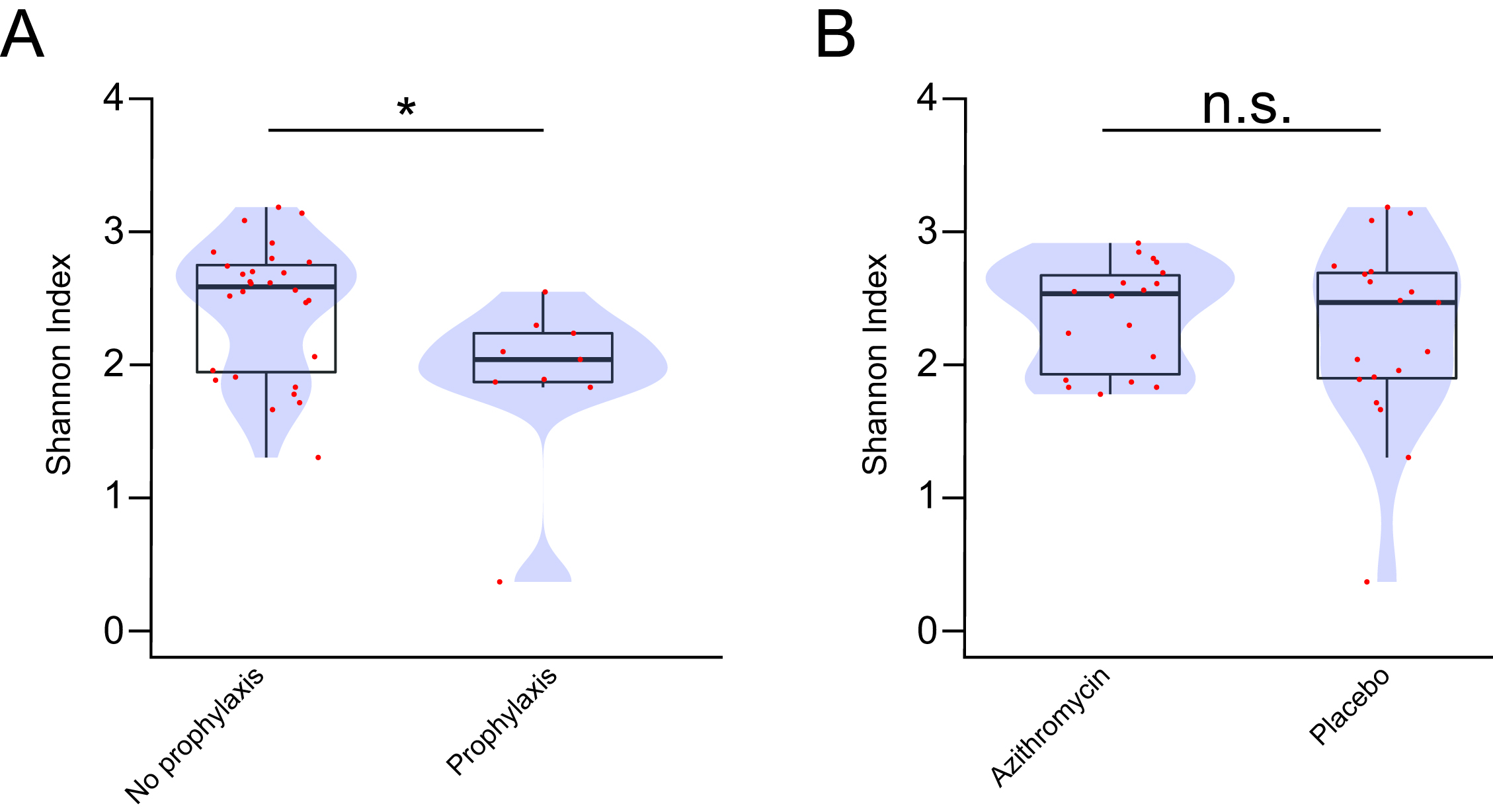


**Figure S13.** **A-B.** Box plots overlaid with density curves (violin plots, blue) representing the fungal diversity (Shannon Index) in BALF with respect to the anti-staphylococcal regimen at the time of BALF collection (**A**), or the treatment arm (azithromycin or placebo) (**B**). Individual data points (red) with jitter are represented on the top of each box plot. Groups were compared using the Wilcoxon rank-sum test: *, *p*<0.05; n.s., no significant (*p*>0.05).


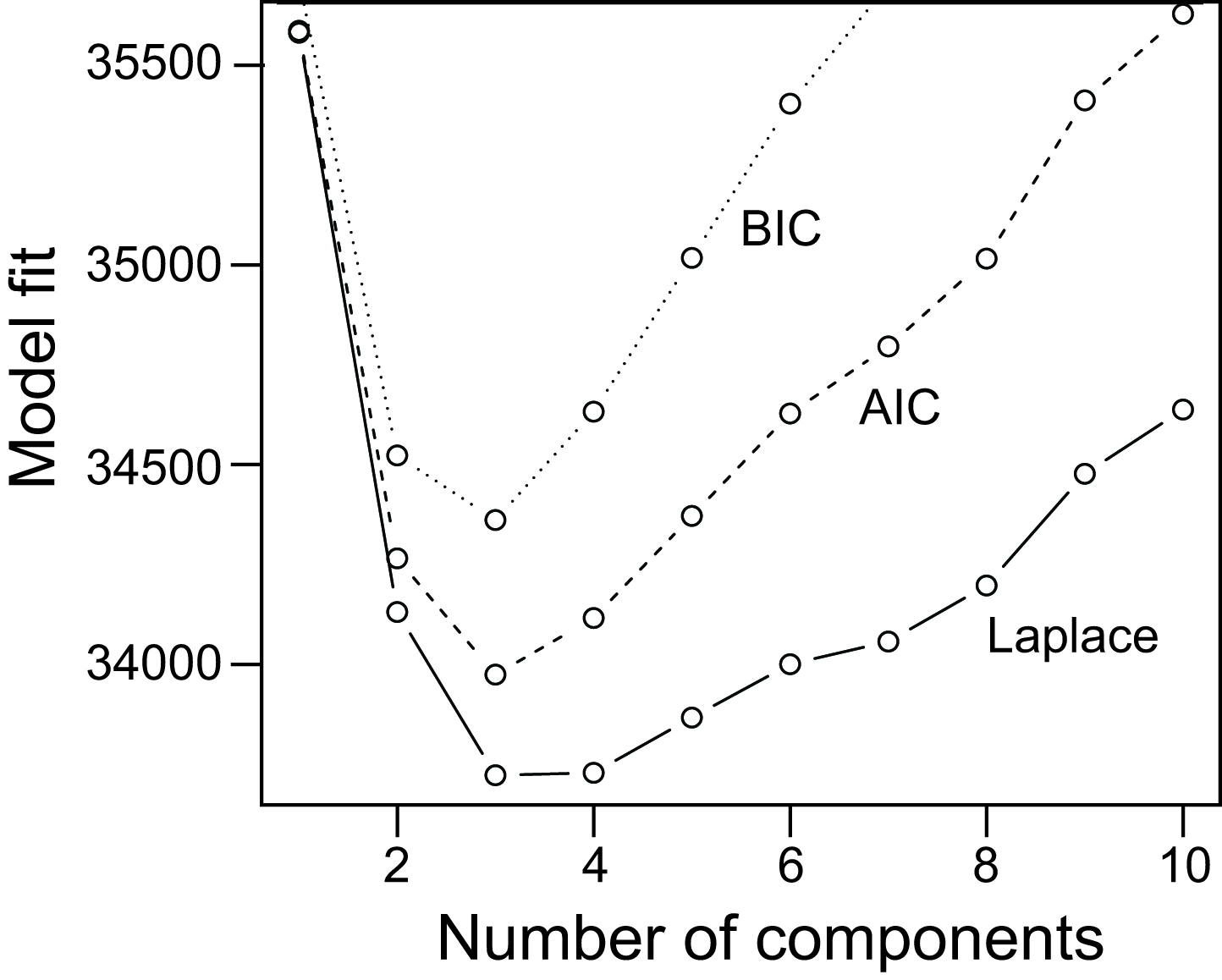


**Figure S14.** Goodness-of-fit measures Bayesian Information Criterion (BIC), Akaike Information Criterion (AIC) and Laplace approximation (Laplace) were use to evaluate the fit of each number of microbial communities to the 16S-based microbial taxonomic profiles.


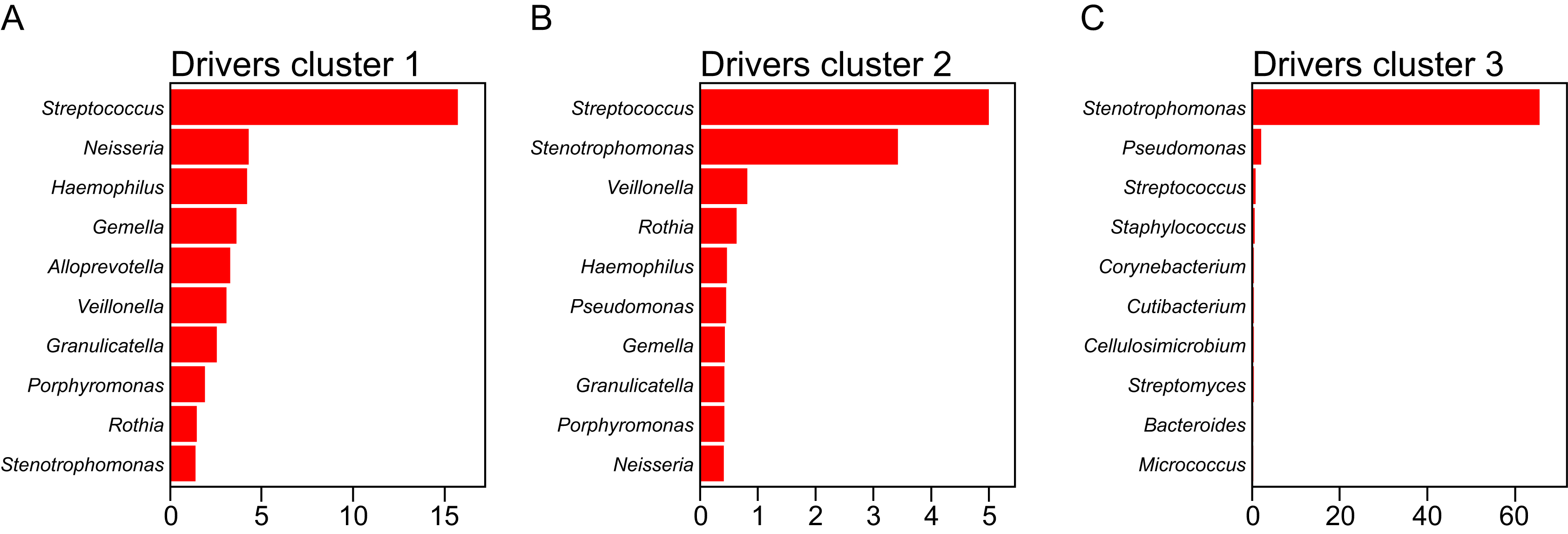


**Figure S15.** Barplots represent the contribution of the indicated taxonomic groups to each metacommunity.


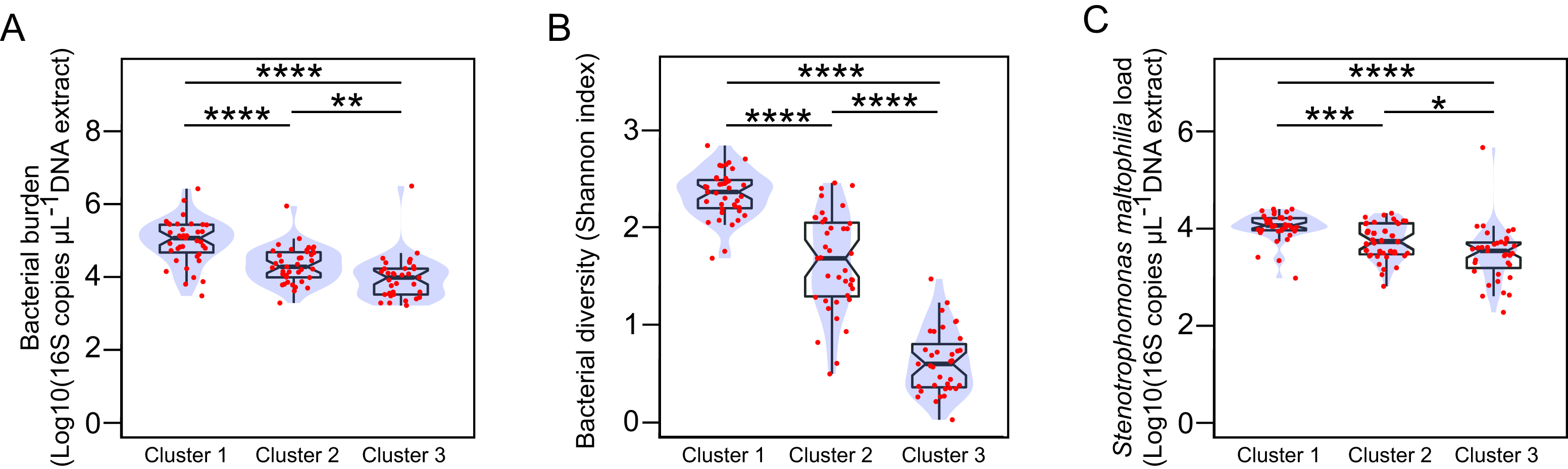


**Figure S16.** Box plots overlaid with density curves (violin plots, blue) representing the bacterial burden (**A**) and diversity (**B**), and the density of *Stenotrophomonas maltophilia* in the DNA extracts from BALF. Individual data points (red) with jitter are represented on the top of each box plot. Notches in the boxplot represent 95% confidence interval for the median. Groups were compared using the Wilcoxon rank-sum test and p-values adjusted for multiple comparisons using the Bonferroni correction: ****, *p*<0.0001; ***, *p*<0.001; **, *p*<0.01; *, *p*<0.05.


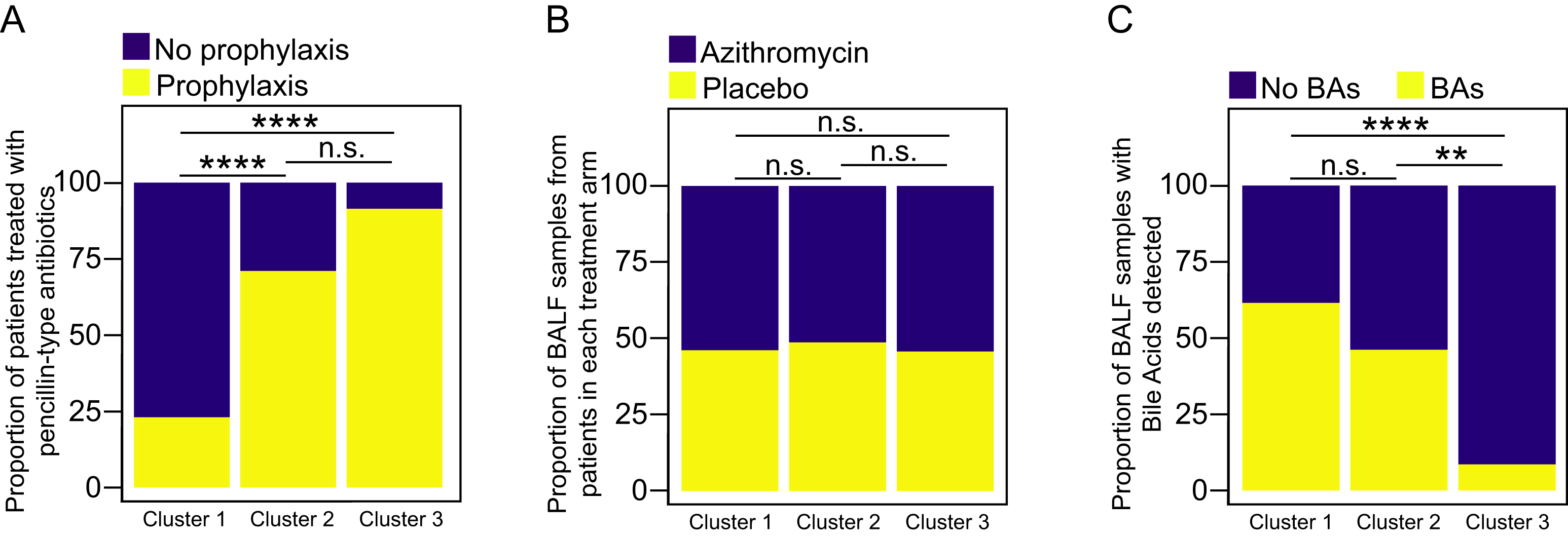


**Figure S17. A-C.** Percent stacked barcharts representing the proportion of BALF samples within each microbial community type collected from patients being treated with penicillin-type antibiotics (**A**) or azithromycin (**B**) at the time of BALF collection, or being positive for bile acid detection (BAs) (**C**). A Fisher’s exact test of independence was used to compare the proportions of BALF samples within each community type. P-values are adjusted using the Bonferroni correction: ****, *p*<0.0001; ***, *p*<0.001; **, *p*<0.01; *, *p*<0.05; n.s., no significant (*p*>0.05).


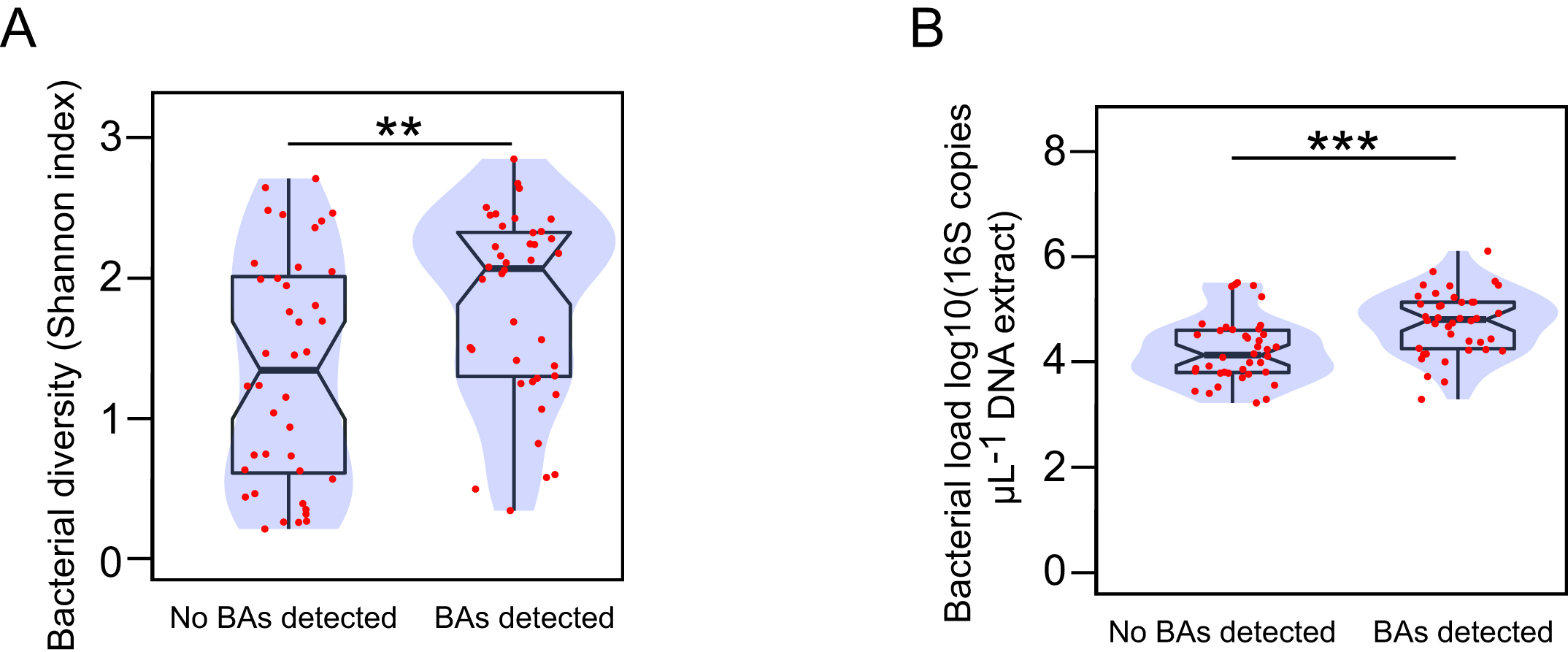


**Figure S18.** Related to Figure 3. Sensitivity analysis controlling for potential batch effects. See material and methods and Figure S37. **A-B.** Box plots overlaid with density curves (violin plots, blue) representing the bacterial diversity (**A**) and burden (**B**) in BALF with respect to treatment with penicillin-like antibiotics. Individual data points (red) with jitter are represented on the top of each box plot. Notches in the boxplot represent 95% confidence interval for the median. Groups were compared using the Wilcoxon rank-sum test: ***, *p*<0.001; **, *p*<0.01.


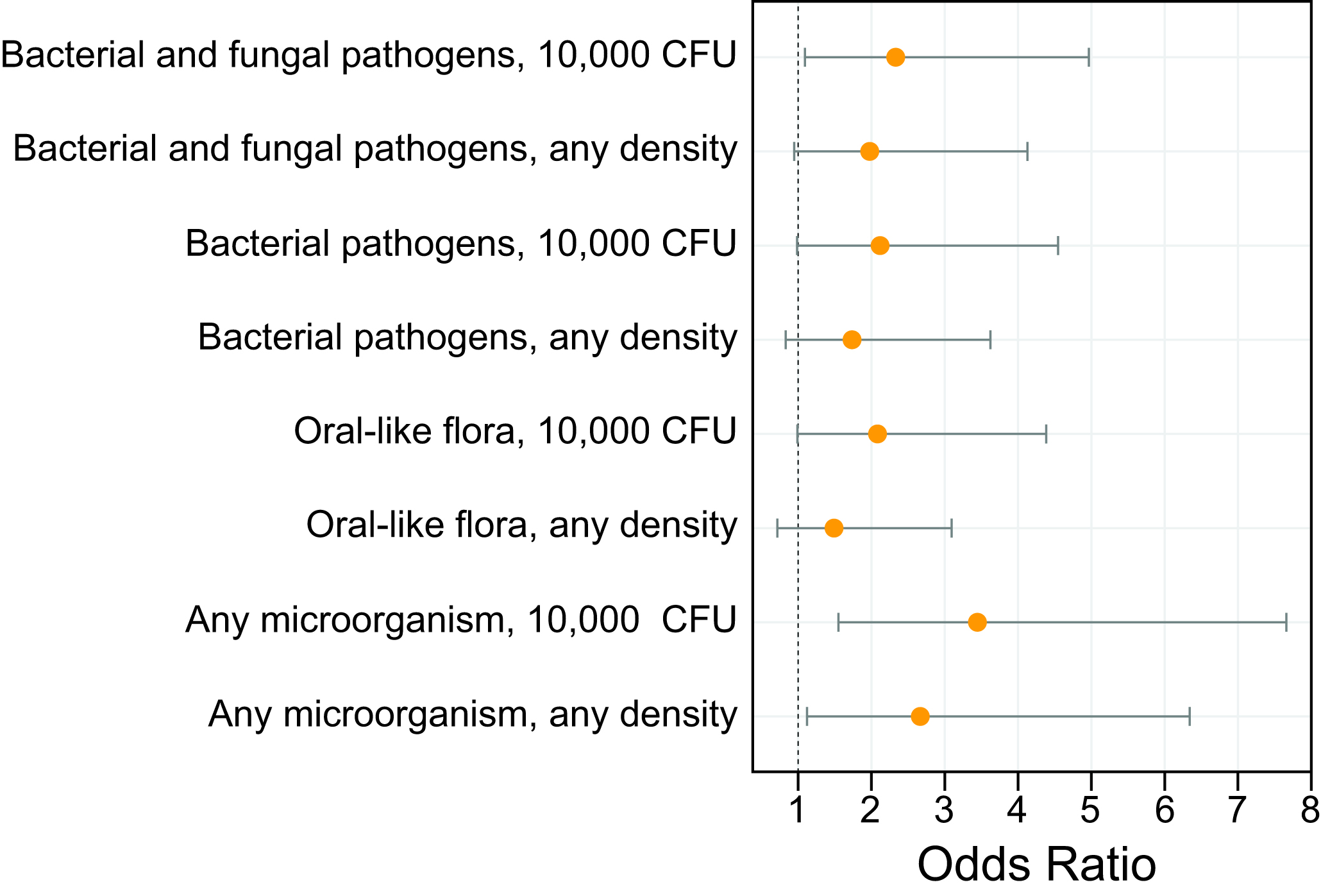


**Figure S19.** The forest plot graphs the association between the detection of bile acids in BALF and the recovery of viable microorganisms using culture-based methods at any density, or at a density equal or higher than 10,000 cfu. Odds ratios with pointwise 95% confidence intervals are plotted. Oral-like flora represents the growth of these microorganisms: Mixed oral flora, *Streptococcus*, *Streptococcus salivarius*, *Streptococcus viridans*, *Neisseria*, *Rothia mucilaginosa*, *Propionibacterium acnes*, *Corynebacterium*. Bacterial pathogens group represents the following microorganisms: methicillin-resistant *Staphylococcus aureus*, *Pseudomonas aeruginosa*, *Moraxella catharralis*, *Streptococcus pneumoniae*, *Stenotrophomonas maltophilia*, *Staphylococcus aureus*, *Haemophilus influenza*, *Escherichia coli*, *Kebsiella oxytoca*, coagulase negative *Staphylococcus*, *Staphylococcus*, *Haemophilus*, *Haemophilus parainfluenzae*, mixed gram negative, *Enterobacter cloacae*, *Citrobacter freundii*, *Sphingomonas paucimobilis*, *Serratia marescens*. The following microorganisms are considered as fungal pathogens: *Candida albicans*, *Candida sp*, *Aspergillus*, *Aspergillus fumigatus*.


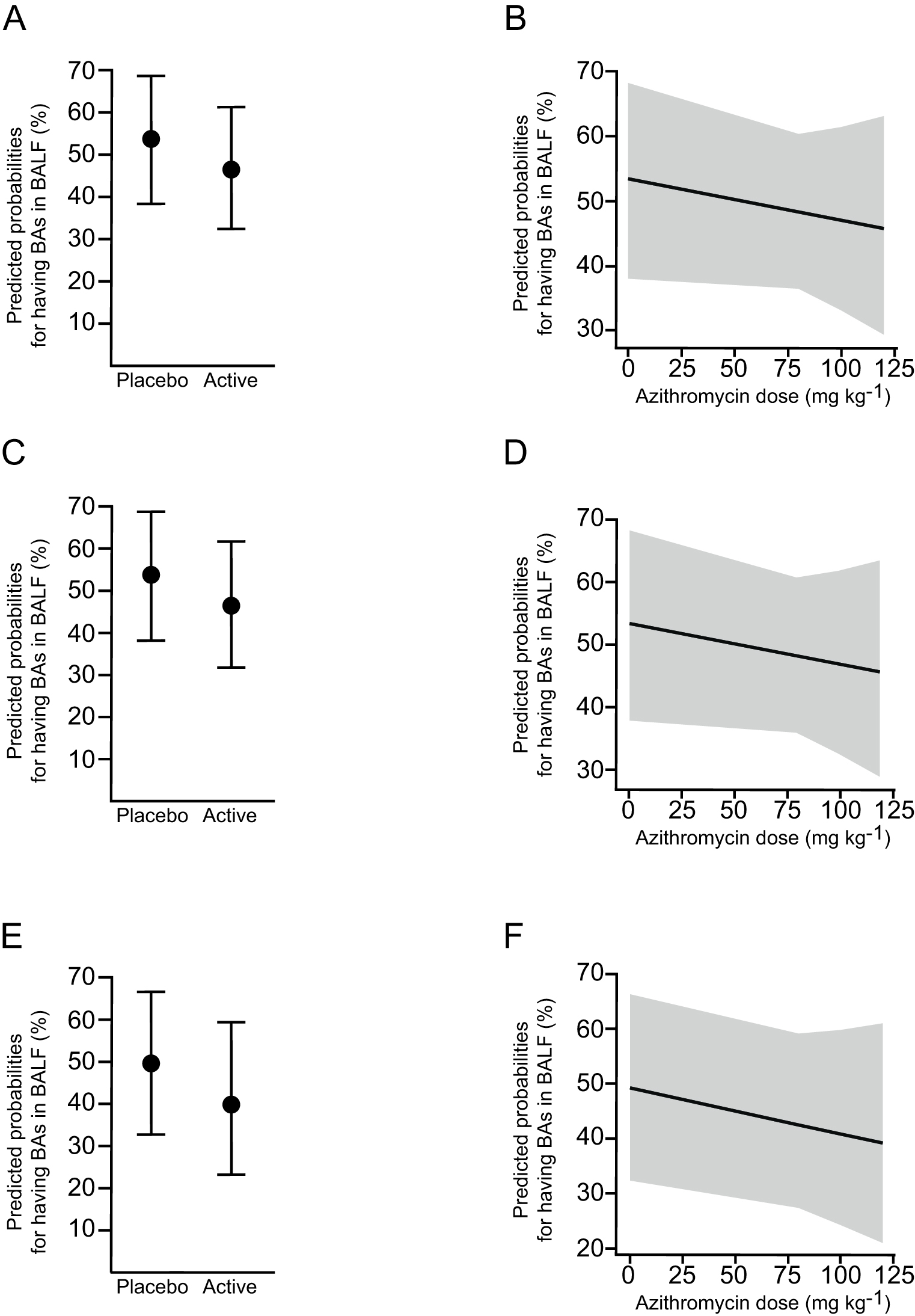


**Figure S20.** Related to Figure 4. Sensitivity analysis controlling for potential batch effects. See material and methods and Figure S37. **A-F.** Marginal effect of treatment arm (**A, C, E**) or azithromycin dosage (**B, D, F**), on the odds of detecting bile acids with pointwise 95% confidence intervals, calculated from logistic regression models. **C-F**. Predicted probabilities after controlling for pancreatic insufficiency (**C-D**) or p.F508del homozigosity (**E-F**).


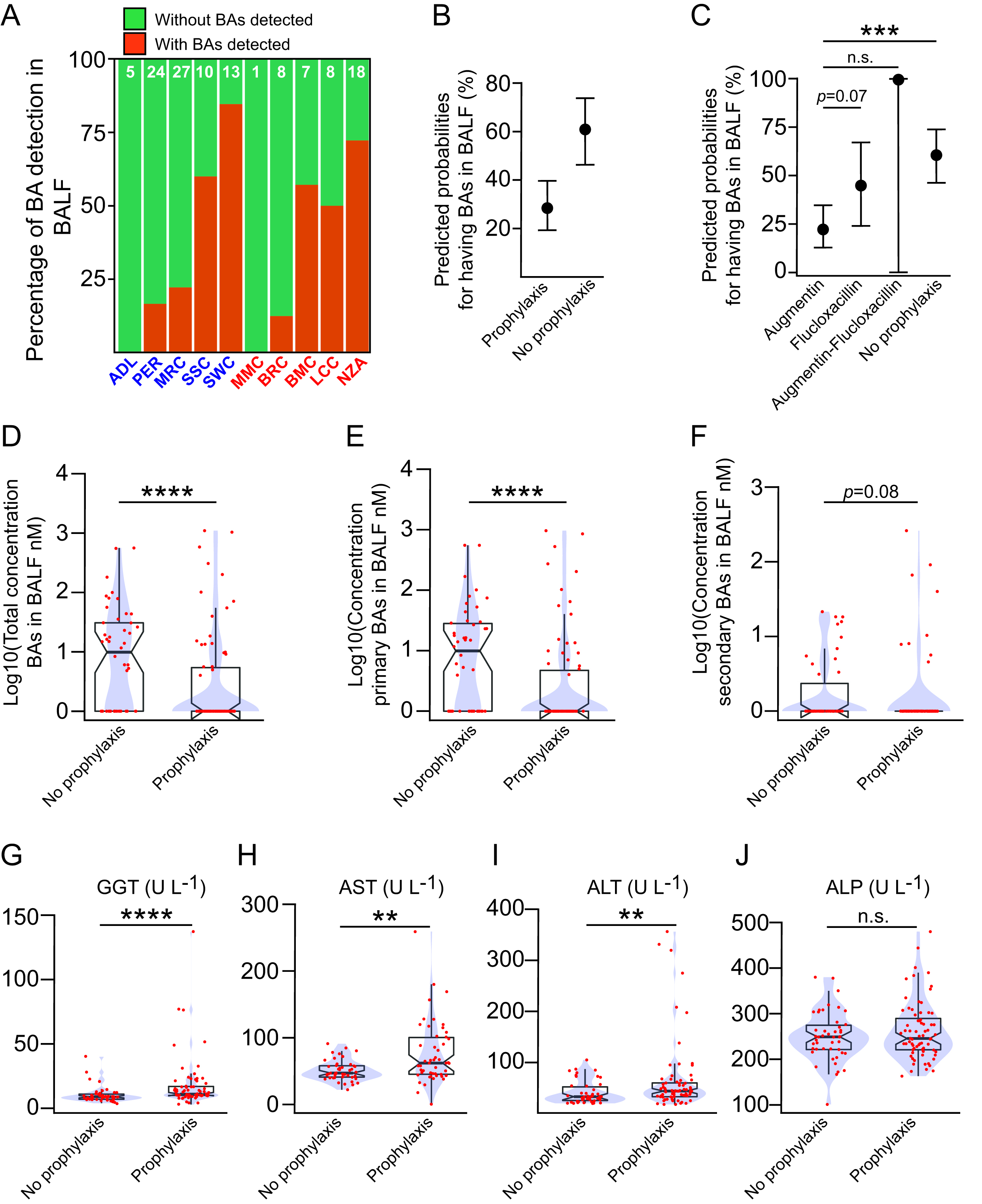


**Figure S21.** **A.** Percentage of BALF samples with bile acid detection in each participating Centre. Each Hospital is identified with a three-letter code. Centres routinely performing anti-*Staphylococcus* prophylaxis are labelled in blue. Centres not administering penicillin-type prophylaxis are depicted in red.  The number of BALF specimens collected from each Centre is indicated in white over the corresponding bar. **B-C.** Marginal effect of anti-*Staphylococcus* prophylaxis (**B**) and specific penicillin-type antibiotic regimens (**C**), on the odds of detecting bile acids with pointwise 95% confidence intervals, calculated from logistic regression models. P-values in **C** represent the level of statistical significance using a Wald test for the difference in log odds between each category and the reference group (Augmentin). ***, *p*<0.001; n.s., no significant (*p*>0.05). **D-J**. Box plots overlaid with density curves (violin plots, blue) representing the concentration of bile acids in BALF (**D-F**), and the levels of circulating markers of liver damage (**G-J**) related to anti-*Staphylococcus* prophylaxis at the time of BALF collection. Concentrations of primary and secondary bile acids are represented in **E** and **F** respectively.  Individual data points (red) with jitter are represented on the top of each box plot. Notches in the boxplot represent 95% confidence interval for the median. In **D-J**, groups were compared using the Wilcoxon rank-sum test. ****, *p*<0.0001; **, *p*<0.01; n.s., no significant (*p*>0.05). Abbreviations: ADL, Women’s and Children’s Hospital Adelaide; PER, Princess Margaret Hospital Perth; MRC, Royal Children’s Hospital Melbourne; SSC, Sydney Children’s Hospital; SWC, The Children’s Hospital at Westmead Sydney; MMC, Monash Medical Centre Melbourne; BRC, Royal Children’s Hospital Brisbane; BMC, Mater Children’s Hospital Brisbane; LCC, Queensland Children’s Hospital Brisbane; NZA, Starship Children’s Hospital Auckland; GGT, gamma glutamyl transpeptidase; ALT, alanine aminotransferase; AST, aspartate aminotransferase; ALP, alkaline phosphatase.

**
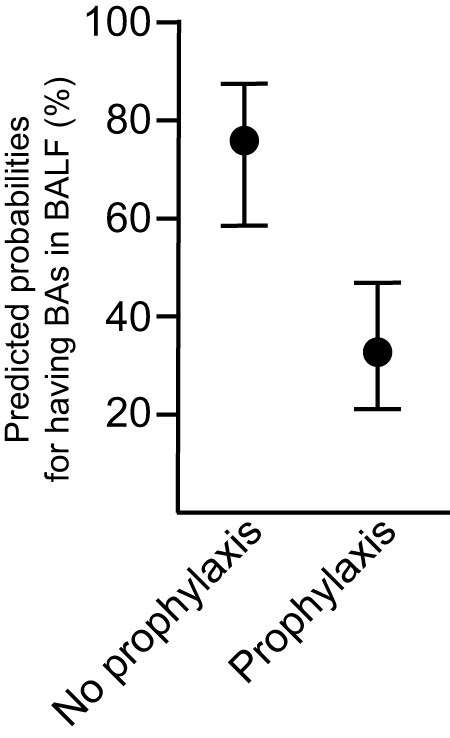
**

**Figure S22.** Related to Figure S20B. Sensitivity analysis controlling for potential batch effects. See material and methods and Figure S37. Marginal effect of anti-*Staphylococcus* prophylaxis on the odds of detecting bile acids with pointwise 95% confidence intervals, calculated from logistic regression models.

**
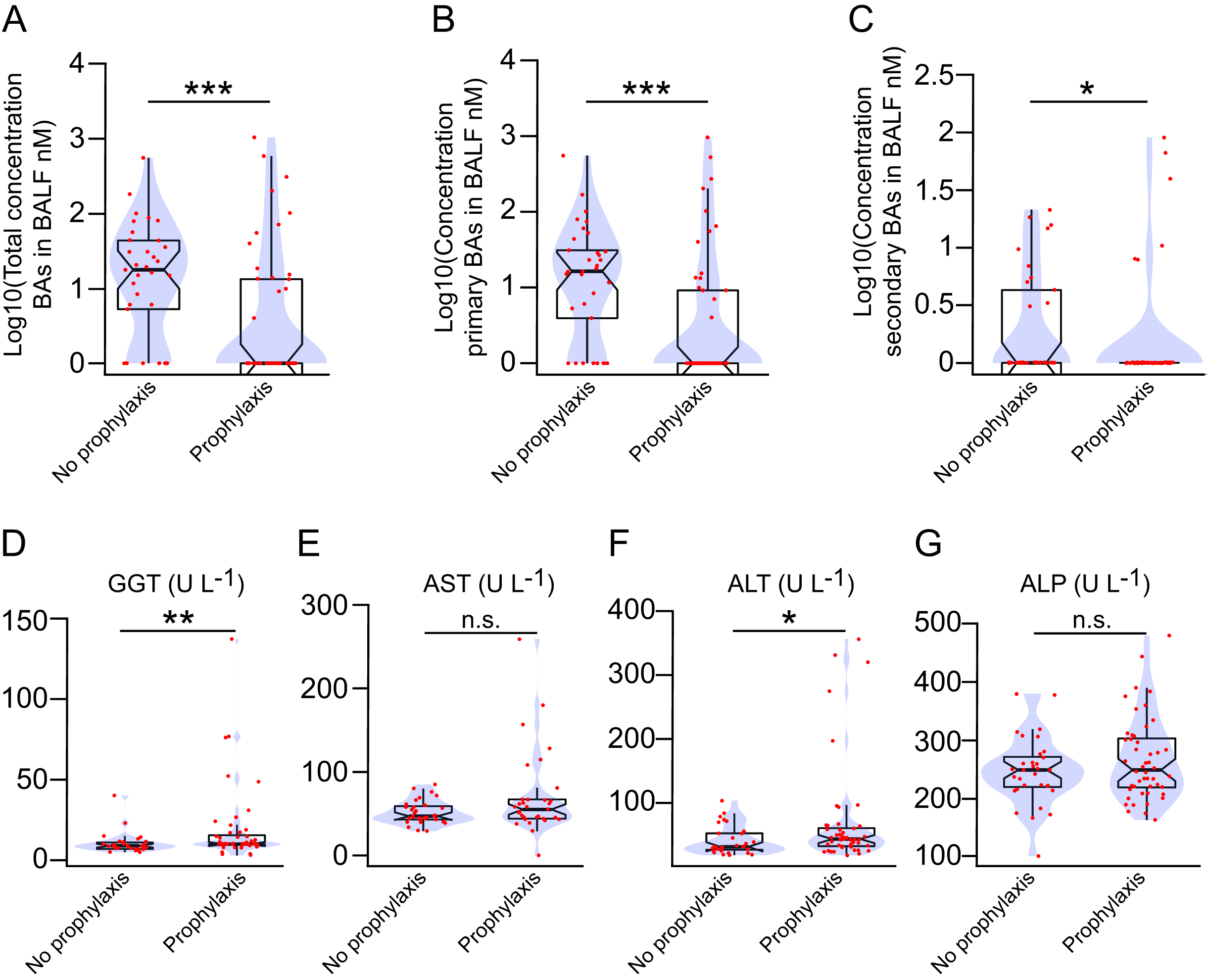
**

**Figure S23.** Related to Figure S20D-J. Sensitivity analysis controlling for potential batch effects. See material and methods and Figure S37. **A-G**. Box plots overlaid with density curves (violin plots, blue) representing the concentration of bile acids in BALF (**A-C**), and the levels of circulating markers of liver damage (**D-G**) related to anti-*Staphylococcus* prophylaxis at the time of BALF collection. Concentrations of primary and secondary bile acids are represented in **B** and **C** respectively. Individual data points (red) with jitter are represented on the top of each box plot. Notches in the boxplot represent 95% confidence interval for the median. Groups were compared using the Wilcoxon rank-sum test. ***, *p*<0.0001; **, *p*<0.01; *, *p*<0.05; n.s., no significant (*p*>0.05). Abbreviations: GGT, gamma glutamyl transpeptidase; ALT, alanine aminotransferase; AST, aspartate aminotransferase; ALP, alkaline phosphatase.

**
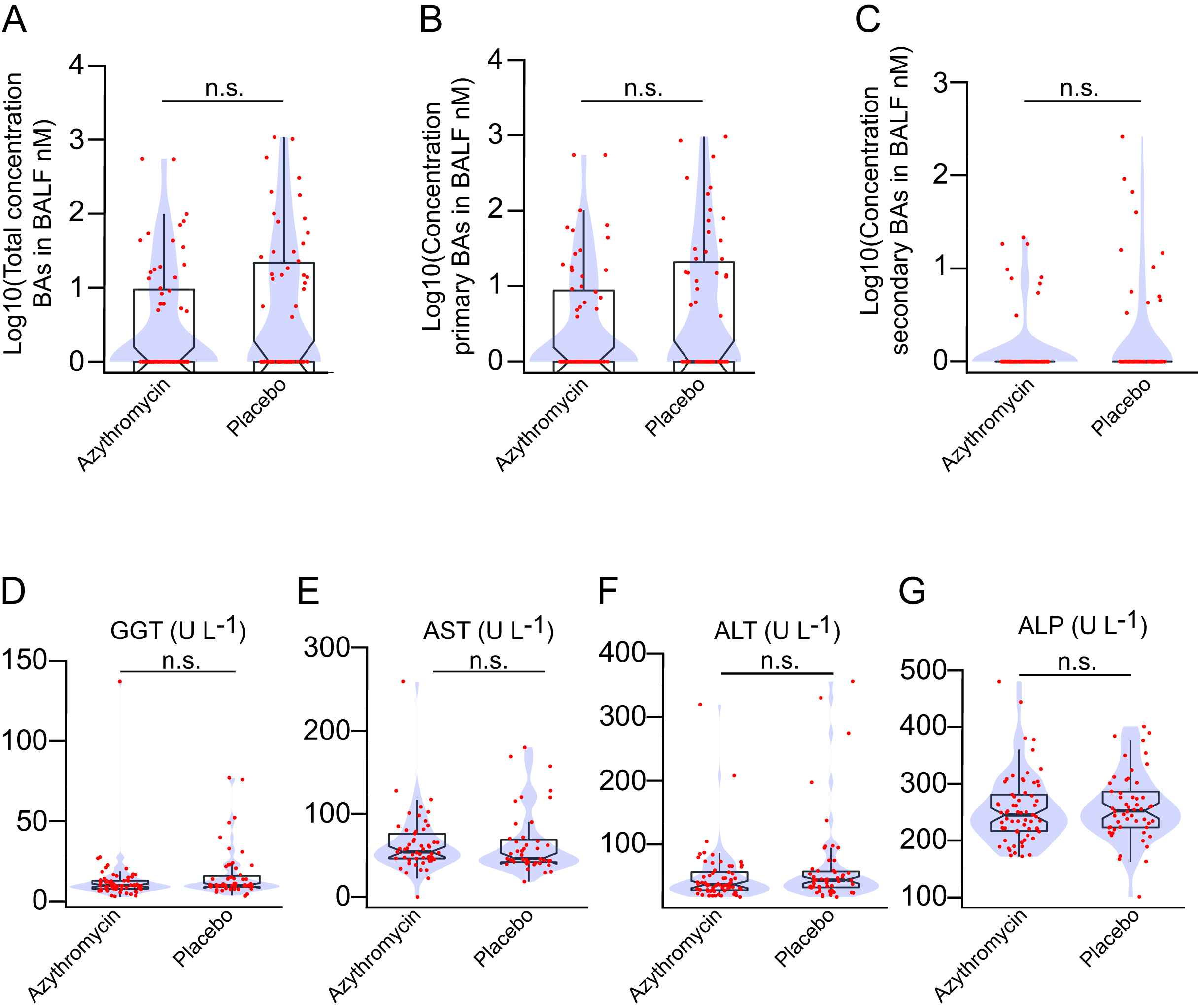
**

**Figure S24.** **A-G.** Box plots overlaid with density curves (violin plots, blue) representing the concentration of bile acids in BALF (**A-C**), and the levels of circulating markers of liver damage (**D-G**) related to the treatment arm (azithromycin or placebo). Concentrations of primary and secondary bile acids are represented in **B** and **C** respectively. Individual data points (red) with jitter are represented on the top of each box plot. Notches in the boxplot represent 95% confidence interval for the median. Groups were compared using the Wilcoxon rank-sum test: n.s., no significant (*p*>0.05). Abbreviations: GGT, gamma glutamyl transpeptidase; ALT, alanine aminotransferase; AST, aspartate aminotransferase; ALP, alkaline phosphatase.

**
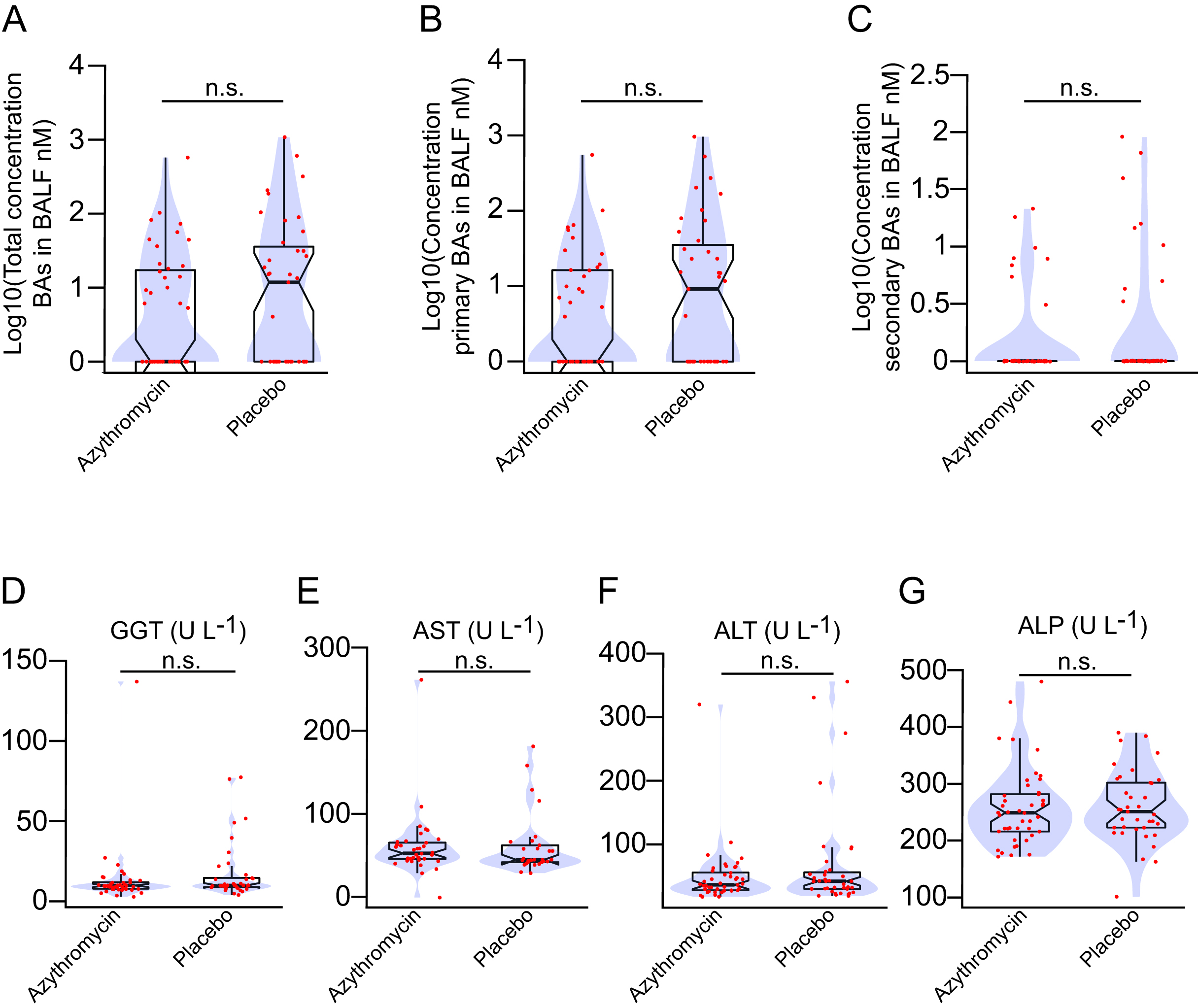
**

**Figure S25.** Related to Figure S18. Sensitivity analysis controlling for potential batch effects. See material and methods and Figure S37. **A-G.** Box plots overlaid with density curves (violin plots, blue) representing the concentration of bile acids in BALF (**A-C**), and the levels of circulating markers of liver damage (**D-G**) related to the treatment arm (azithromycin or placebo). Concentrations of primary and secondary bile acids are represented in **B** and **C** respectively. Individual data points (red) with jitter are represented on the top of each box plot. Notches in the boxplot represent 95% confidence interval for the median. Groups were compared using the Wilcoxon rank-sum test: n.s., no significant (*p*>0.05). Abbreviations: GGT, gamma glutamyl transpeptidase; ALT, alanine aminotransferase; AST, aspartate aminotransferase; ALP, alkaline phosphatase.


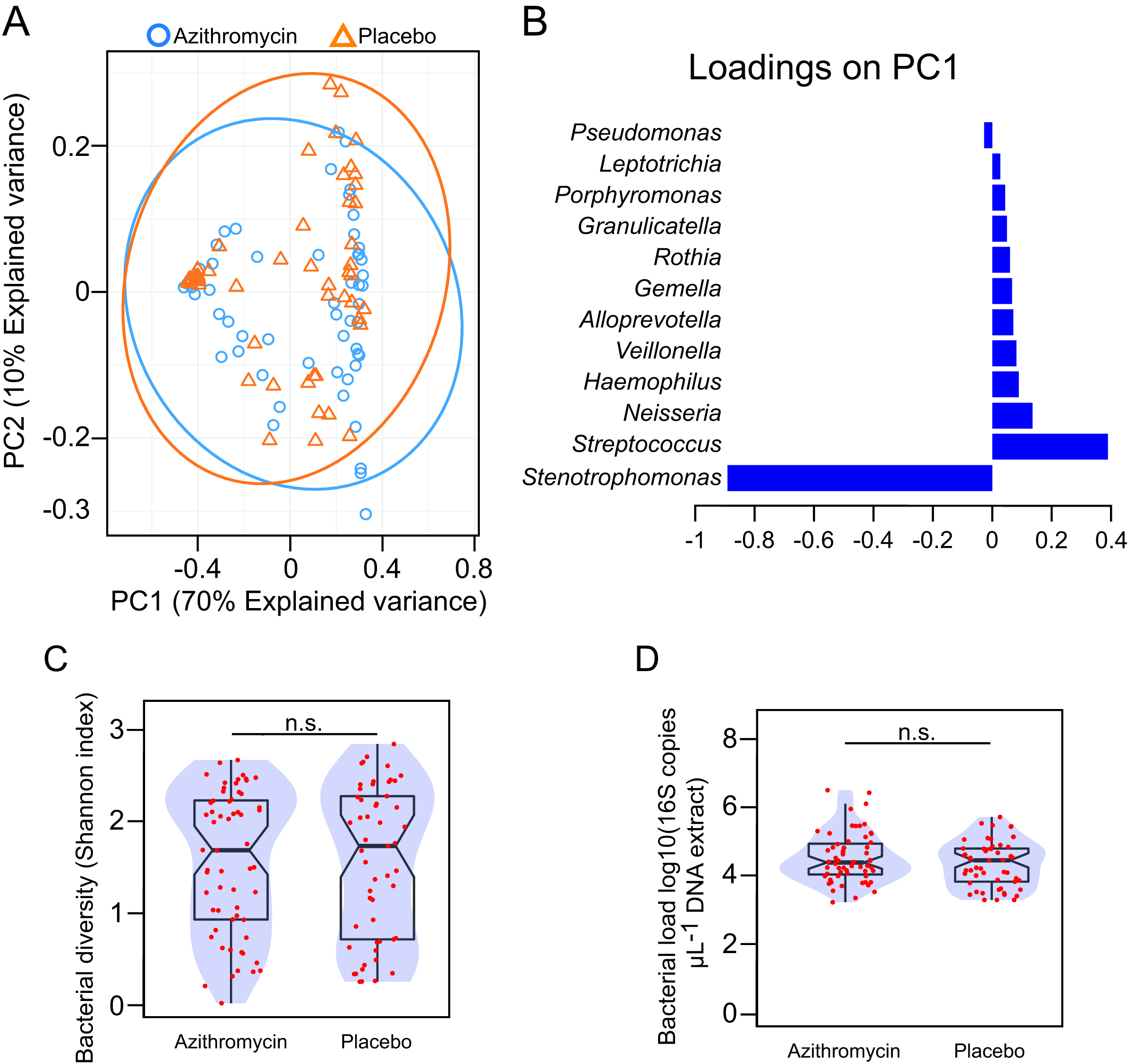


**Figure S26.** **A.** Principal component analysis (PCA) plot shows the linear projection of the 16S-based compositional profiles onto a two-dimensional space. Each sample is labelled based on the treatment arm membership: azithromycin (blue circle) or placebo (orange triangle). **B.** Loadings for PC1 representing the weights of the indicated variables defining component 1. **C-D.** Box plots overlaid with density curves (violin plots, blue) representing the bacterial diversity (**C**) and burden (**D**) in BALF with respect to the treatment arm (azithromycin or placebo). Individual data points (red) with jitter are represented on the top of each box plot. Notches in the boxplot represent 95% confidence interval for the median. Groups were compared using the Wilcoxon rank-sum test: n.s., no significant (*p*>0.05).


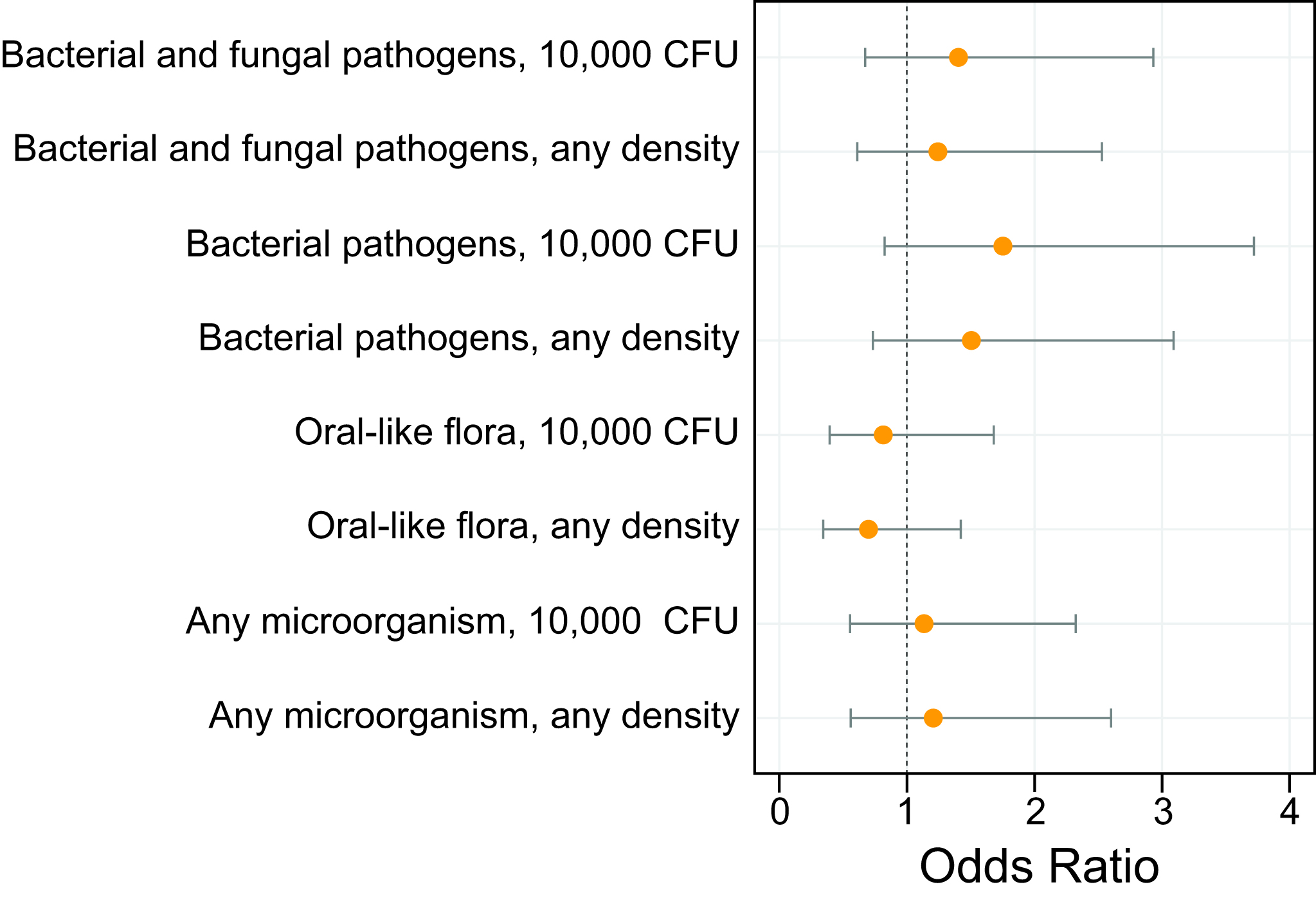


**Figure S27.** The forest plot graph the association between azithromycin therapy and the recovery from BALF of viable microorganisms using culture-based methods at any density, or at a density equal or higher than 10,000 cfu. Odds ratios with pointwise 95% confidence intervals are plotted. Oral-like flora represents the growth of these microorganisms: Mixed oral flora, *Streptococcus*, *Streptococcus salivarius*, *Streptococcus viridans*, *Neisseria*, *Rothia mucilaginosa*, *Propionibacterium acnes*, *Corynebacterium*. Bacterial pathogens group represents the following microorganisms: methicillin-resistant *Staphylococcus aureus*, *Pseudomonas aeruginosa*, *Moraxella catharralis*, *Streptococcus pneumoniae*, *Stenotrophomonas maltophilia*, *Staphylococcus aureus*, *Haemophilus influenza*, *Escherichia coli*, *Kebsiella oxytoca*, coagulase negative *Staphylococcus*, *Staphylococcus*, *Haemophilus*, *Haemophilus parainfluenzae*, mixed gram negative, *Enterobacter cloacae*, *Citrobacter freundii*, *Sphingomonas paucimobilis*, *Serratia marescens*. The following microorganisms are considered as fungal pathogens: *Candida albicans*, *Candida sp*, *Aspergillus*, *Aspergillus fumigatus*.


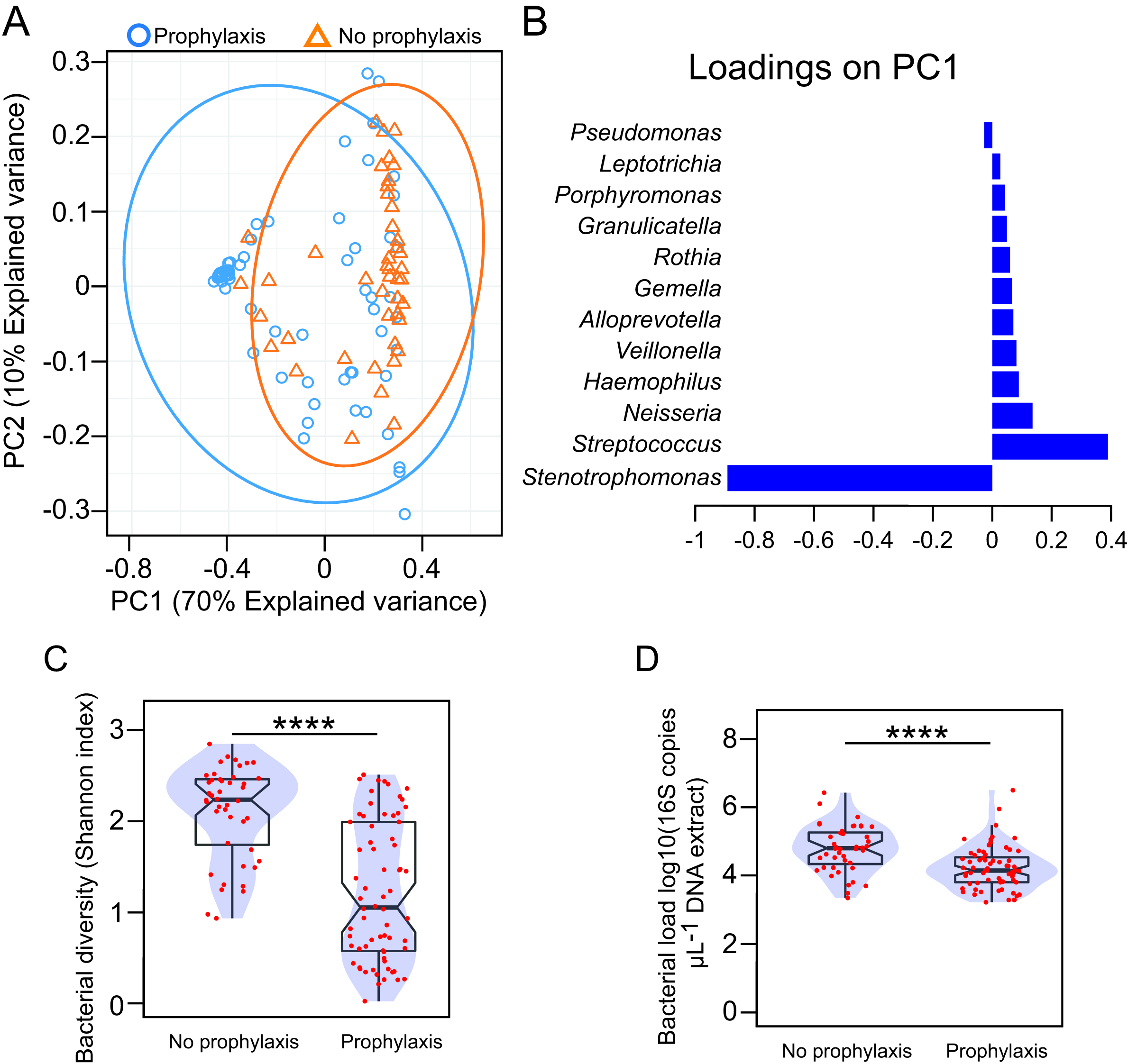


**Figure S28.** **A.** Principal component analysis (PCA) plot shows the linear projection of the 16S-based compositional profiles onto a two-dimensional space. Each sample is labelled based on anti-Staphylococcus prophylaxis regimen at the time of BALF collection: prophylaxis (blue circle) or no prophylaxis (orange triangle). **B.** Loadings for PC1 representing the weights of the indicated variables defining component 1. **C-D.** Box plots overlaid with density curves (violin plots, blue) representing the bacterial diversity (**C**) and burden (**D**) in BALF with respect to treatment with penicillin-like antibiotics. Individual data points (red) with jitter are represented on the top of each box plot. Notches in the boxplot represent 95% confidence interval for the median. Groups were compared using the Wilcoxon rank-sum test: ****, *p*<0.0001.


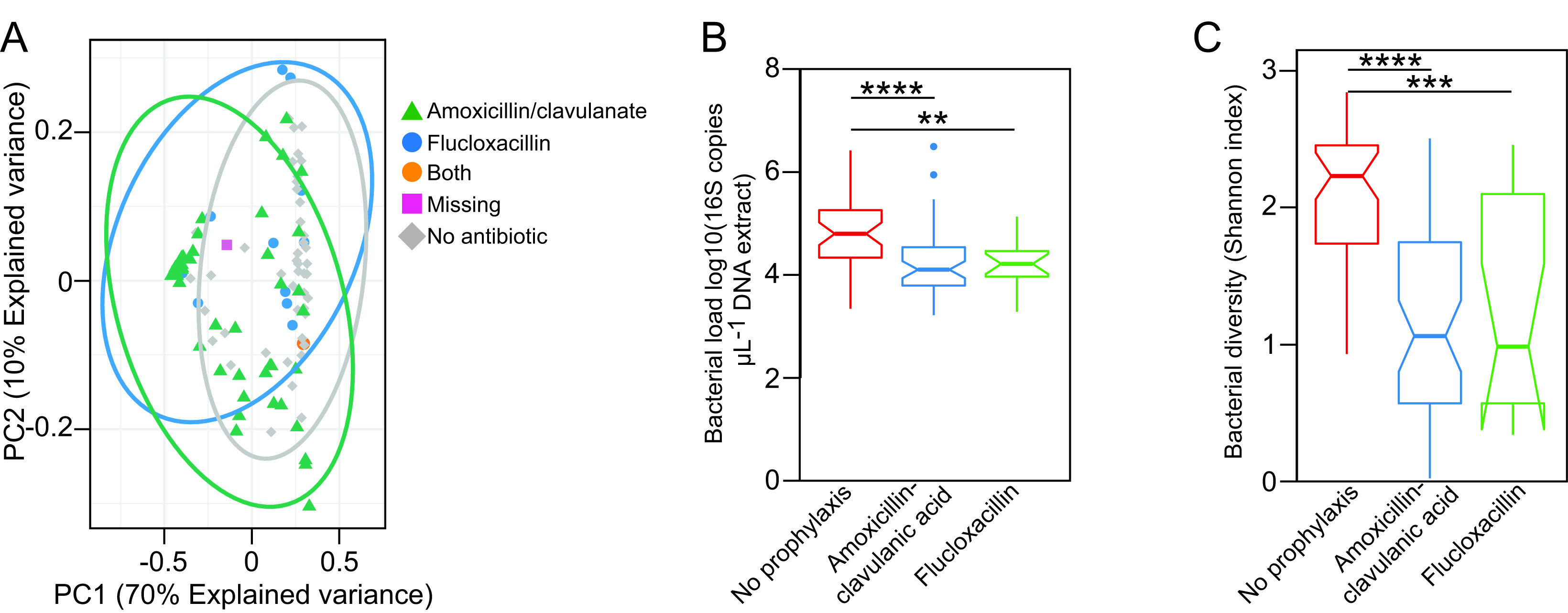


**Figure S29.** **A.** Principal component analysis (PCA) plot shows the linear projection of the 16S-based compositional profiles onto a two-dimensional space. Each sample is labelled based on the type of penicillin-like therapy at the time of BALF collection. “Both” refer to flucloxacillin and augmentin regimen **B-C.** Box plots representing total bacterial burden (**B)** and the microbial diversity (**C**) in BALF with respect to the type of penicillin-like antibiotic taken. Notches represent 95% confidence interval for the median. Groups were compared using one-way ANOVA and *p*-values estimated with Dunnett’s posthoc test: ****, *p*<0.0001; ***, *p*<0.001; **, *p*<0.01.


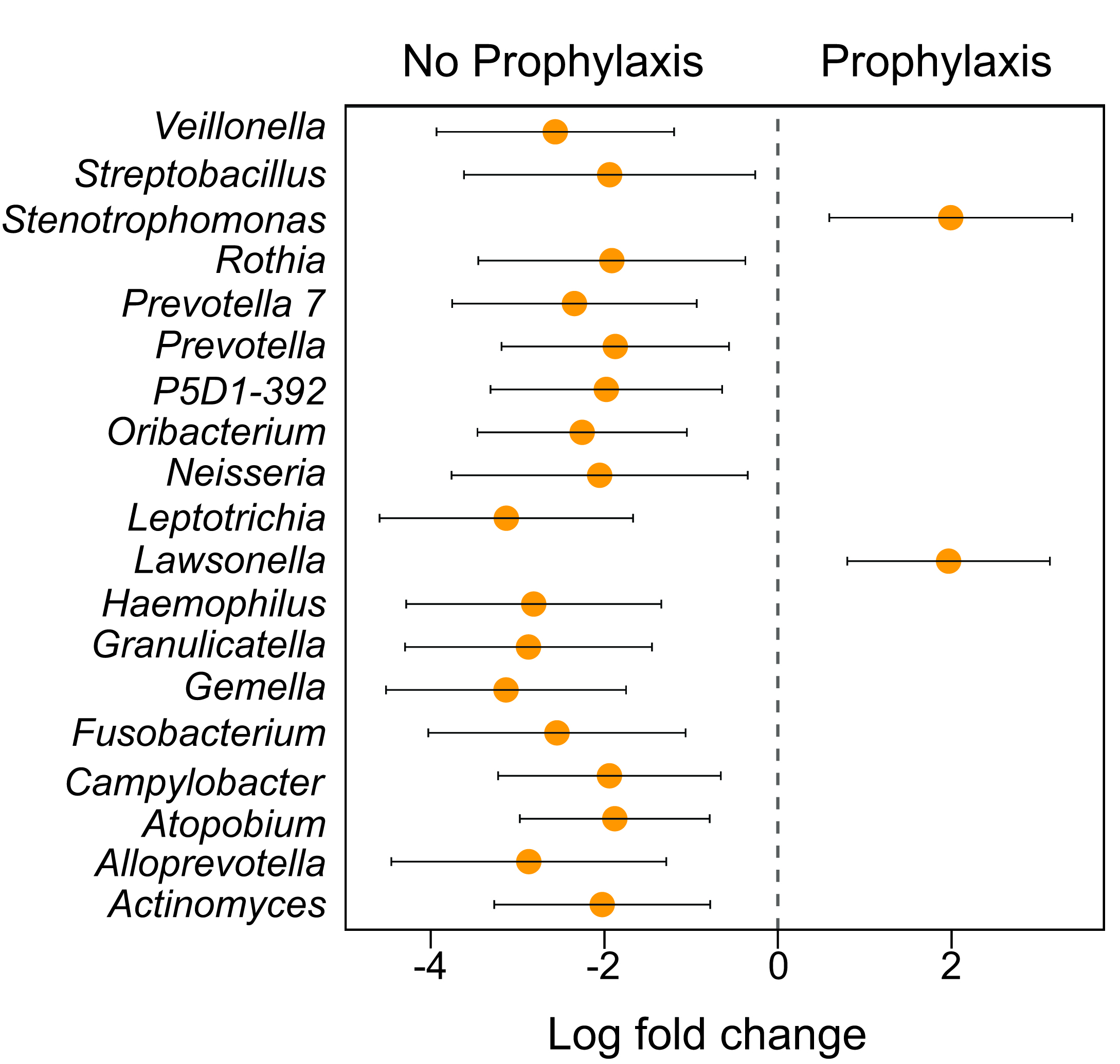


**Figure S30** The dotplot displays the results of the differential abundance analysis between BALF-associated bacterial communities from patients taking anti-staphylococcal prophylaxis or not at the time of BALF collection. Coefficients from the ANCOMBC log linear model with pointwise 95% Bonferroni-corrected confidence intervals are plotted. Only statistically significant features with an absolute Log fold change value higher than 1.8 are represented. Taxonomic entities enriched in BALF samples from patients taking or not penicillin-like antibiotics at the time of BALF collection are represented with positive and negative fold change values respectively.


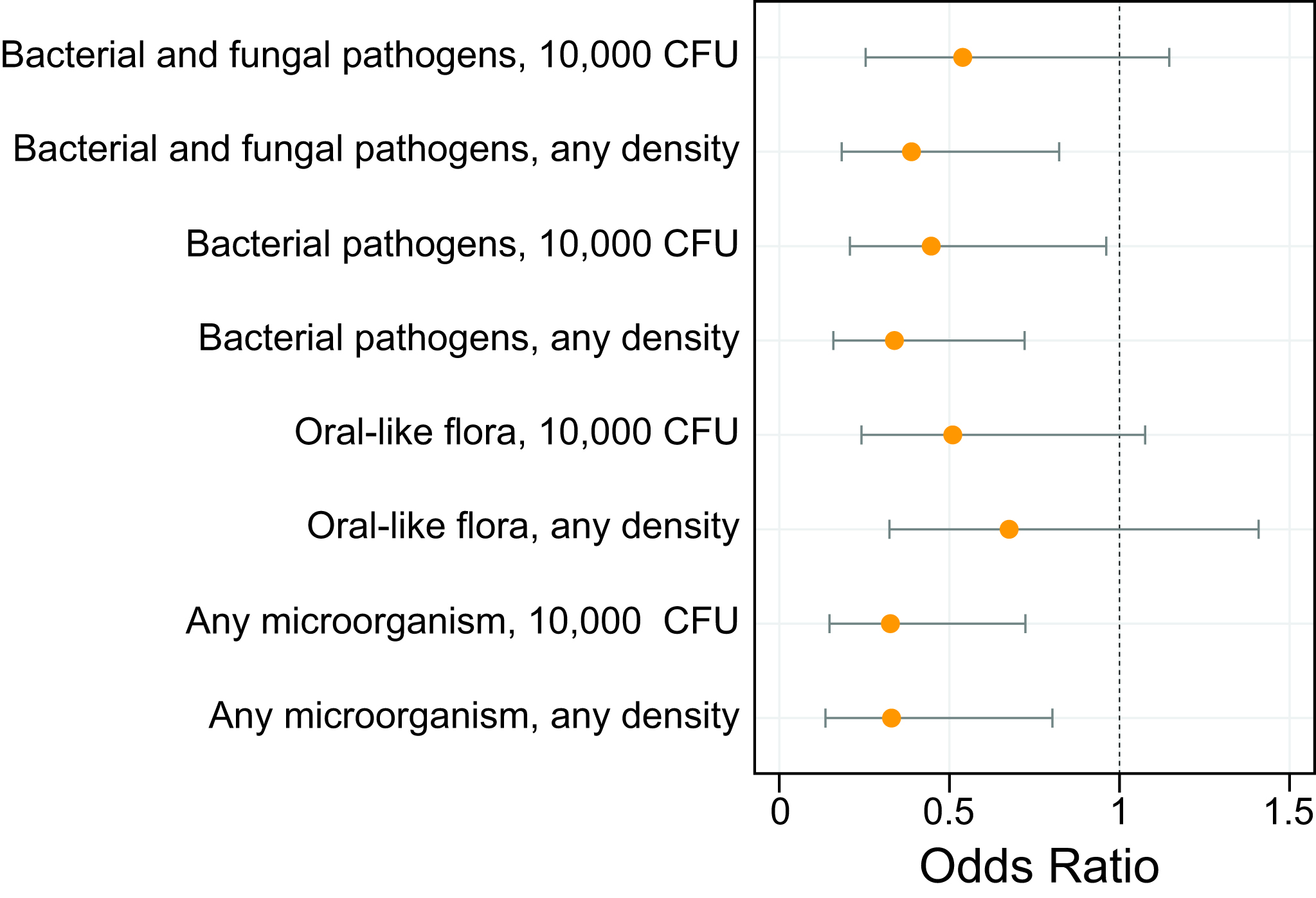


**Figure S31.** The forest plot graph the association between the penicillin-type prophylaxis and the recovery from BALF of viable microorganisms using culture-based methods at any density, or at a density equal or higher than 10,000 cfu. Odds ratios with pointwise 95% confidence intervals are plotted. Oral-like flora represents the growth of these microorganisms: Mixed oral flora, *Streptococcus*, *Streptococcus salivarius*, *Streptococcus viridans*, *Neisseria*, *Rothia mucilaginosa*, *Propionibacterium acnes*, *Corynebacterium*. Bacterial pathogens group represents the following microorganisms: methicillin-resistant *Staphylococcus aureus*, *Pseudomonas aeruginosa*, *Moraxella catharralis*, *Streptococcus pneumoniae*, *Stenotrophomonas maltophilia*, *Staphylococcus aureus*, *Haemophilus influenza*, *Escherichia coli*, *Kebsiella oxytoca*, coagulase negative *Staphylococcus*, *Staphylococcus*, *Haemophilus*, *Haemophilus parainfluenzae*, mixed gram negative, *Enterobacter cloacae*, *Citrobacter freundii*, *Sphingomonas paucimobilis*, *Serratia marescens*. The following microorganisms are considered as fungal pathogens: *Candida albicans*, *Candida sp*, *Aspergillus*, *Aspergillus fumigatus*.


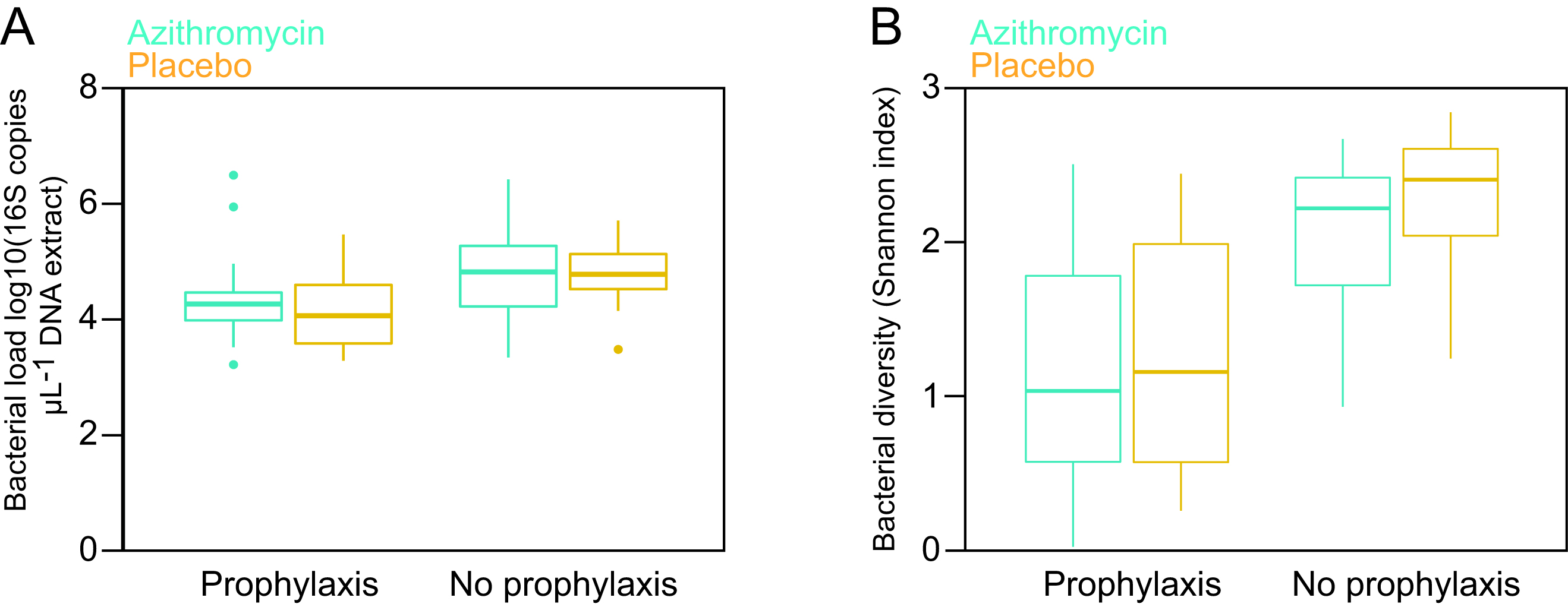


**Figure S32.** **A-B.** Interaction between azithromycin and anti-staphylococcal prophylaxis on bacterial burden (**A**) and diversity (**B**) was evaluated through a two-way ANOVA. Only the effect of prophylaxis was statistically significant at *p*<0.05.

**
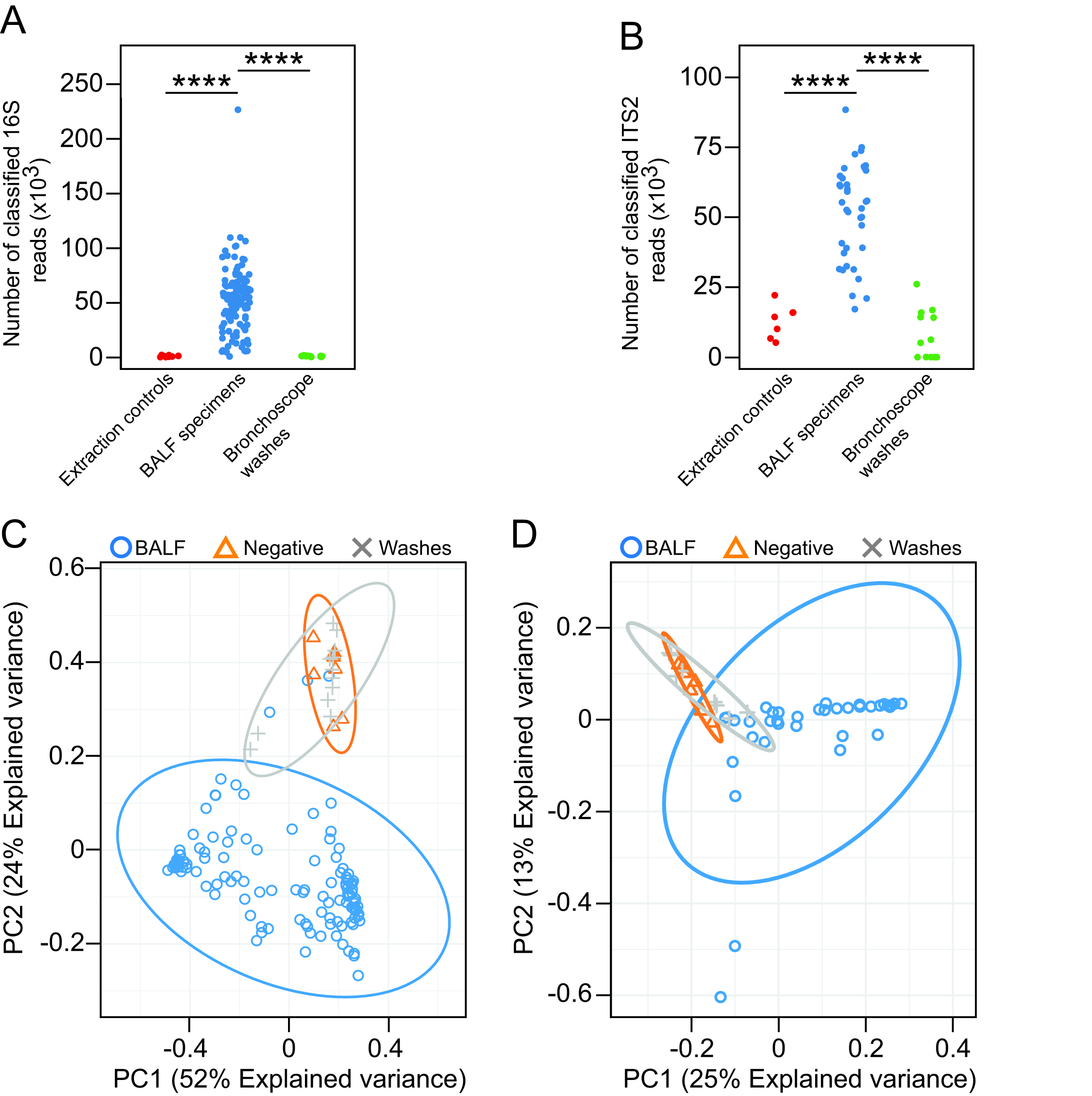
**

**Figure S33.** **A-B.** Dot plots showing the number of 16S (A) and ITS2 (B) reads in BALF samples (blue), negative extraction controls (red) and bronchoscope washes (red). Groups were compared in the context of the Wilcoxon rank-sum test and p-values corrected using the false discovery rate method: ****, *p*<0.0001. **C-D.** Principal component analysis (PCA) plots showing the linear projection of the 16S- (**C**) and ITS2-based (**D**) compositional profiles onto a two-dimensional space. Each sample is labelled based on the group membership: BALF specimens (blue circle), Negative extraction controls (orange triangle) and bronchoscope washes (grey cross). Ellipse plots defines 95% confidence region for the indicated sample groups.

**
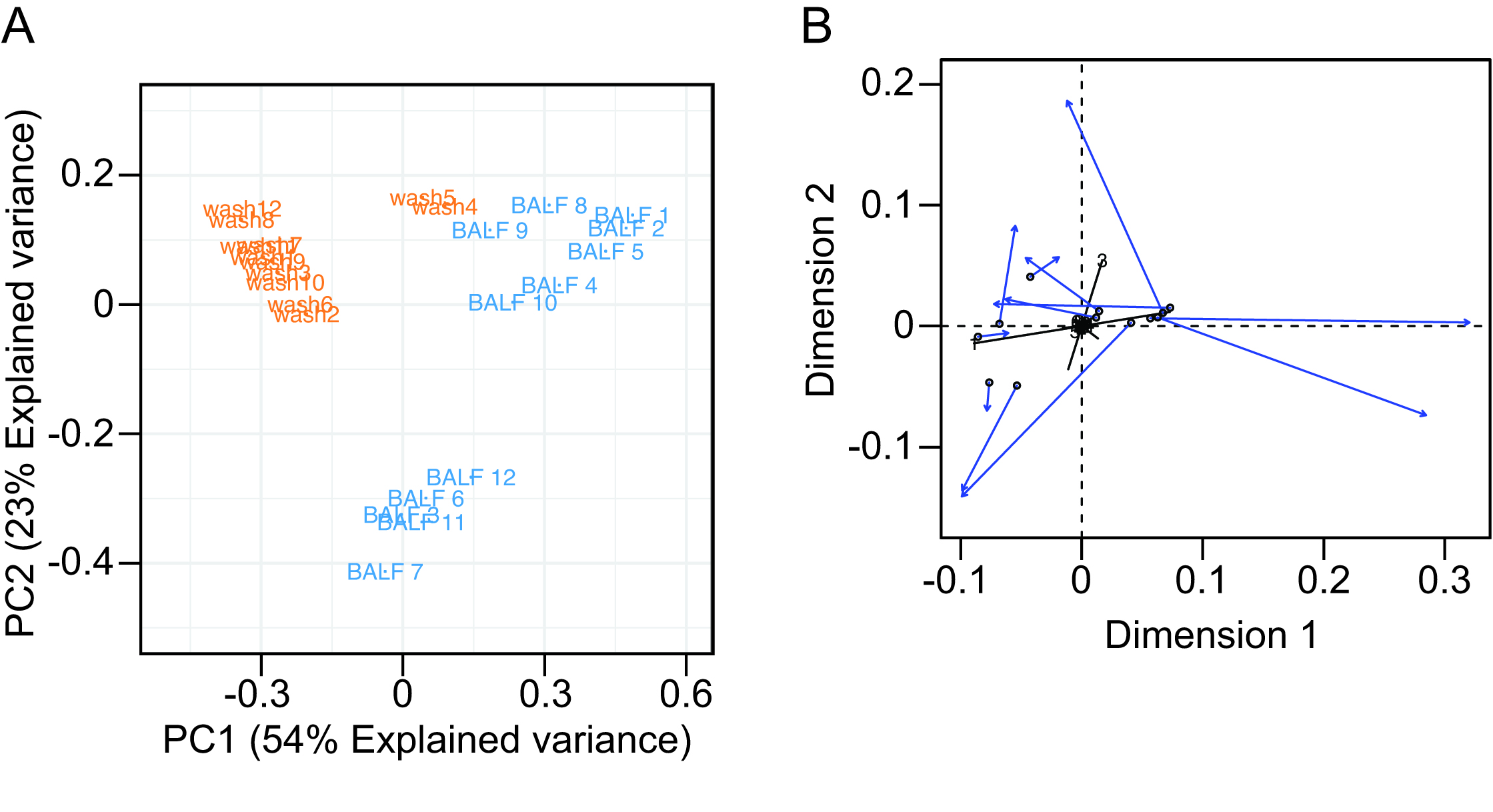
**

**Figure S34­.** **A.** PCA plot showing the linear projection of the BALF samples (BALF, blue coloured) and the corresponding bronchoscope washes (wash, orange coloured) based on their bacterial compositional profiles. Paired BALF and bronchoscope washes are indicated with the same number. **B.** Procrustes analysis of the correlation between the bronchoscope washes and the paired BALF samples. Circles represent the spatial location of the BALF samples in the ordination to be rotated (PCA in BALF specimens), and blue arrows point to location of the paired bronchoscope wash in the target ordination (PCA in bronchoscope washes). Solid black lines indicate the rotation of the indicated axes required to match samples in both ordinations as close as possible.


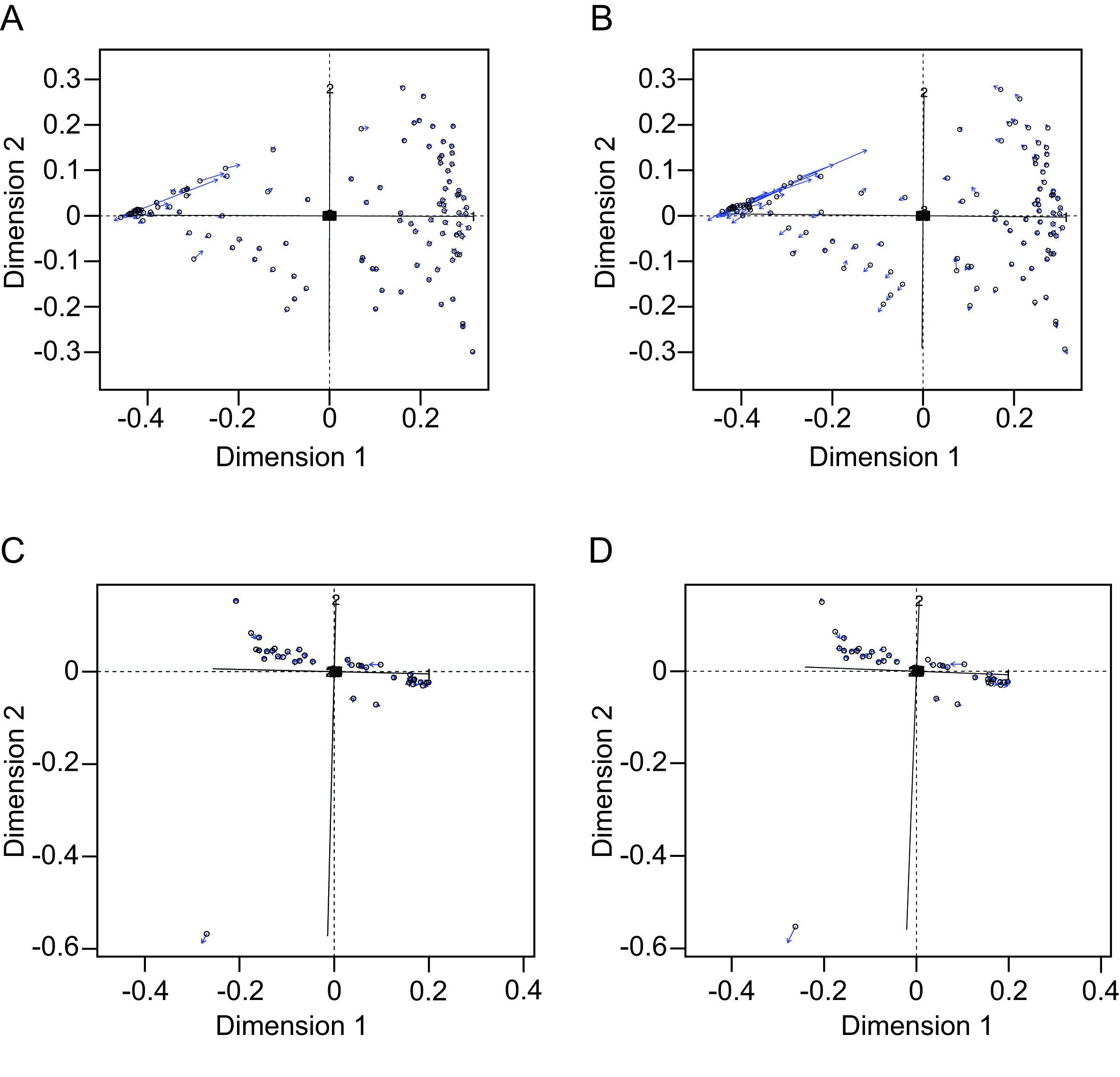


**Figure S35.** **A-D.** Procrustes analysis shows that the filtering steps did not remarkably impact the composition of the original bacterial (**A-B**) and fungal (**C-D**) profiles. Procrustes analyses of the correlation between the unfiltered dataset and the dataset without low counts, singletons and unclassified reads are shown in **A** and **C**. Plots in B and D, represent the degree of match between the microbial profiles in the original dataset and the dataset without low counts, singletons, unclassified reads and putative contaminants. Circles represent the spatial location of the samples in the first ordination (filtered dataset), and blue arrows point to location of the respective sample in the second ordination (unfiltered dataset). Solid black lines indicate the rotation of the indicated axes required to match samples in both ordinations as close as possible.


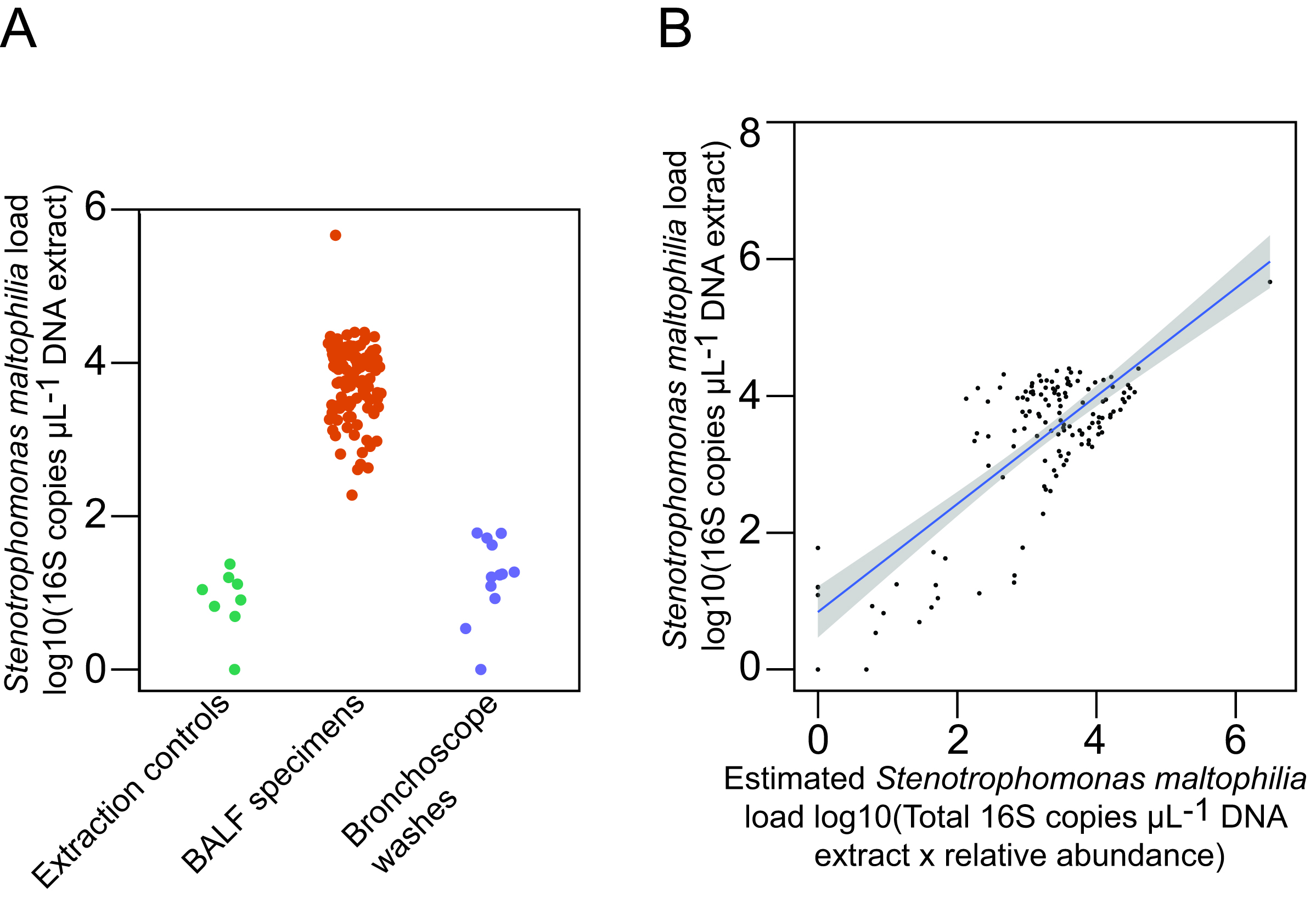


**Figure S36.** **A**. Quantification of *Stenotrophomonas maltophilia* genetic material in the DNA extracts from the indicated sample type. **B**. Relationship between the absolute load of *Stenotrophomonas maltophilia* DNA using a specific qPCR assay, and the estimated abundance (relative abundance multiplied by the total bacterial load). The blue line and shaded areas represent the regression line and 95% confidence interval respectively. The good fit of the model suggests that the estimated burden constitutes a good approximation to the absolute load of *Stenotrophomonas maltophilia* (β 0.79, R^2^ 0.60, F(1,131)=198.10 *p*<0.0001)*.*


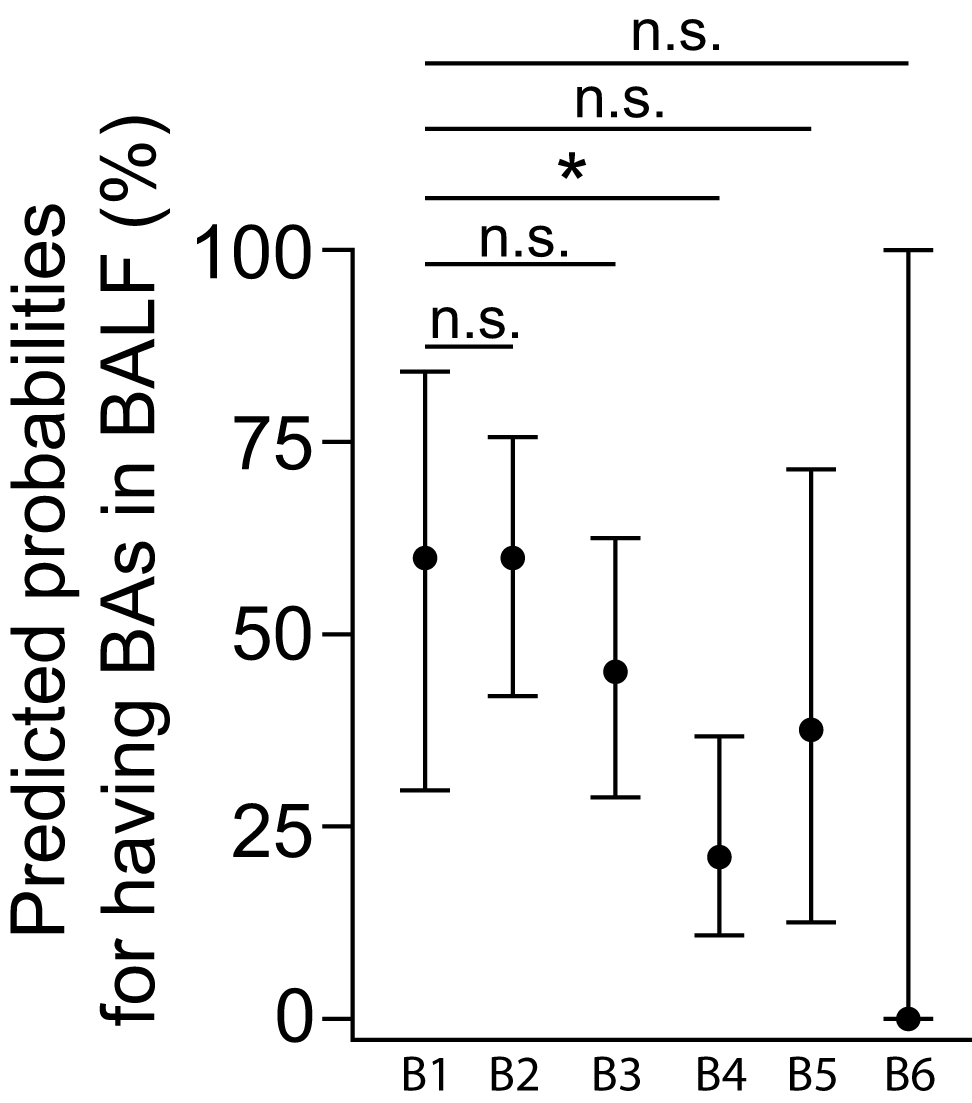


**Figure S37­.** Marginal effect of batch extraction on the odds of detecting bile acids with pointwise 95% confidence intervals, calculated from logistic regression models. Statistical significance was assessed using a Wald test for the difference in log odds between each category and the reference group (Batch 1, B1). *, *p*<0.05; n.s., no significant.

**Table S1.** Patient demographics.

| **Gender** | **Male** | **Female** |  |
| --- | --- | --- | --- |
|  | 72 (59.5%) | 49 (40.5%) |  |
| **CFTR Variant (Zigosity)** | **p. Phe506del (homozygous)** | **p. Phe506del (heterozygous)** | **Other** |
|  | 66 (54.5%) | 48 (39.7%) | 7 (5.8%) |

**Table S2.** Details about the study protocol deviation events. Asterisks indicate a patient who underwent two rounds with macrolides.

| Therapeutic | Study group | Days treated | Treatment end date | BALF collection date |
| --- | --- | --- | --- | --- |
| Azithromycin | Placebo | 28 | 29/11/2013 | 27/02/2014 |
| Azithromycin | Azithromycin | 10 | 9/05/2017 | 29/06/2017 |
| Erythromycin | Placebo | 7 | 29/05/2015 | 30/06/2015 |
| Azithromycin | Placebo* | 4 | 22/08/2014 | 31/10/2014 |
| Erythromycin | Placebo* | 9 | 17/10/2014 | 31/10/2014 |

**Table S3.** Bile acid profiles in BALF (µM). GDCA, glycodeoxycholic acid; GCDCA, glycochenodeoxycholic acid; GUDCA, glycoursodeoxycholic acid; TDCA, taurodeoxycholic acid; TCA, taurocholic acid; CA, cholic acid; TCDCA, taurochenodeoxycholic acid.

| BALF ID | GDCA | GCDCA | GUDCA | TDCA | TCA | CA | TCDCA |
| --- | --- | --- | --- | --- | --- | --- | --- |
| ADL001 | 0 | 0 | 0 | 0 | 0 | 0 | 0 |
| ADL003 | 0 | 0 | 0 | 0 | 0 | 0 | 0 |
| ADL005 | 0 | 0 | 0 | 0 | 0 | 0 | 0 |
| ADL006 | 0 | 0 | 0 | 0 | 0 | 0 | 0 |
| ADL007 | 0 | 0 | 0 | 0 | 0 | 0 | 0 |
| BMC001 | 0 | 0.00736 | 0 | 0 | 0 | 0 | 0 |
| BMC002 | 0 | 0 | 0 | 0 | 0 | 0 | 0 |
| BMC003 | 0 | 0.00508 | 0.00355 | 0 | 0 | 0 | 0 |
| BMC004 | 0 | 0 | 0 | 0 | 0 | 0 | 0 |
| BMC005 | 0 | 0.01828 | 0 | 0 | 0 | 0 | 0 |
| BMC006 | 0 | 0 | 0 | 0 | 0 | 0 | 0 |
| BMC007 | 0 | 0.34090 | 0.00689 | 0.01035 | 0 | 0 | 0.20137 |
| BRC001 | 0 | 0 | 0 | 0 | 0 | 0 | 0 |
| BRC002 | 0 | 0 | 0 | 0 | 0.01292 | 0 | 0 |
| BRC003 | 0 | 0 | 0 | 0 | 0 | 0 | 0 |
| BRC004 | 0 | 0 | 0 | 0 | 0 | 0 | 0 |
| BRC005 | 0 | 0 | 0 | 0 | 0 | 0 | 0 |
| BRC006 | 0 | 0 | 0 | 0 | 0 | 0 | 0 |
| BRC007 | 0 | 0 | 0 | 0 | 0 | 0 | 0 |
| BRC008 | 0 | 0 | 0 | 0 | 0 | 0 | 0 |
| LCC001 | 0 | 0 | 0 | 0 | 0 | 0 | 0 |
| LCC002 | 0 | 0 | 0 | 0 | 0 | 0 | 0 |
| LCC003 | 0 | 0 | 0 | 0 | 0 | 0 | 0 |
| LCC004 | 0 | 0.00427 | 0 | 0 | 0 | 0 | 0 |
| LCC005 | 0 | 0 | 0 | 0 | 0 | 0 | 0 |
| LCC006 | 0 | 0.45676 | 0.00875 | 0 | 0.08517 | 0 | 0 |
| LCC007 | 0 | 0.01063 | 0 | 0 | 0 | 0 | 0 |
| LCC009 | 0 | 0.01428 | 0 | 0 | 0 | 0 | 0 |
| MMC002 | 0 | 0 | 0 | 0 | 0 | 0 | 0 |
| MRC001 | 0 | 0 | 0 | 0 | 0.00459 | 0 | 0 |
| MRC002 | 0 | 0 | 0 | 0 | 0 | 0 | 0 |
| MRC003 | 0 | 0 | 0 | 0 | 0 | 0 | 0 |
| MRC004 | 0 | 0 | 0 | 0 | 0 | 0 | 0 |
| MRC005 | 0 | 0 | 0 | 0 | 0 | 0 | 0 |
| MRC006 | 0 | 0 | 0 | 0 | 0 | 0 | 0 |
| MRC007 | 0 | 0 | 0 | 0 | 0 | 0 | 0 |
| MRC008 | 0 | 0.00887 | 0 | 0 | 0 | 0 | 0 |
| MRC009 | 0 | 0 | 0 | 0 | 0 | 0 | 0 |
| MRC010 | 0 | 0 | 0 | 0 | 0 | 0 | 0 |
| MRC011 | 0 | 0.00812 | 0.00934 | 0 | 0 | 0 | 0 |
| MRC012 | 0 | 0.01394 | 0 | 0 | 0 | 0 | 0 |
| MRC013 | 0 | 0 | 0 | 0 | 0 | 0 | 0 |
| MRC015 | 0 | 0 | 0 | 0 | 0 | 0 | 0 |
| MRC016 | 0 | 0.00812 | 0 | 0 | 0 | 0 | 0 |
| MRC017 | 0 | 0 | 0 | 0 | 0 | 0 | 0 |
| MRC018 | 0 | 0 | 0 | 0 | 0 | 0 | 0 |
| MRC019 | 0 | 0 | 0 | 0 | 0 | 0 | 0 |
| MRC020 | 0 | 0 | 0 | 0 | 0 | 0 | 0 |
| MRC021 | 0 | 0 | 0 | 0 | 0 | 0 | 0 |
| MRC023 | 0 | 0 | 0 | 0 | 0 | 0 | 0 |
| MRC024 | 0 | 0 | 0 | 0 | 0 | 0 | 0 |
| MRC025 | 0 | 0.01241 | 0 | 0 | 0 | 0 | 0 |
| MRC026 | 0 | 0 | 0 | 0 | 0 | 0 | 0 |
| MRC027 | 0 | 0 | 0 | 0 | 0 | 0 | 0 |
| MRC028 | 0 | 0 | 0 | 0 | 0 | 0 | 0 |
| NZA001 | 0 | 0.01382 | 0 | 0 | 0 | 0 | 0 |
| NZA002 | 0 | 0.07249 | 0 | 0.01361 | 0 | 0 | 0 |
| NZA003 | 0 | 0.02187 | 0.00328 | 0 | 0 | 0 | 0 |
| NZA004 | 0 | 0.00504 | 0 | 0 | 0 | 0 | 0 |
| NZA005 | 0 | 0.02557 | 0.01731 | 0 | 0 | 0 | 0 |
| NZA006 | 0 | 0.05879 | 0.02041 | 0 | 0 | 0 | 0 |
| NZA007 | 0 | 0.02736 | 0.00232 | 0 | 0 | 0 | 0 |
| NZA008 | 0 | 0.05115 | 0.00401 | 0 | 0 | 0 | 0 |
| NZA009 | 0 | 0.00293 | 0.00212 | 0 | 0 | 0 | 0 |
| NZA010 | 0 | 0.01669 | 0 | 0 | 0 | 0 | 0 |
| NZA011 | 0 | 0 | 0 | 0 | 0 | 0 | 0 |
| NZA012 | 0 | 0.02198 | 0 | 0 | 0 | 0 | 0 |
| NZA014 | 0 | 0 | 0 | 0 | 0 | 0 | 0 |
| NZA015 | 0 | 0 | 0 | 0 | 0 | 0 | 0 |
| NZA016 | 0 | 0 | 0 | 0 | 0 | 0 | 0 |
| NZA017 | 0 | 0.01509 | 0 | 0.00447 | 0 | 0 | 0 |
| NZA018 | 0 | 0 | 0 | 0 | 0 | 0 | 0 |
| NZA020 | 0 | 0.07769 | 0 | 0 | 0 | 0 | 0 |
| PER001 | 0 | 0 | 0 | 0 | 0 | 0 | 0 |
| PER002 | 0 | 0 | 0 | 0 | 0 | 0 | 0 |
| PER003 | 0 | 0 | 0 | 0 | 0 | 0 | 0 |
| PER004 | 0 | 0 | 0 | 0 | 0 | 0 | 0 |
| PER005 | 0 | 0 | 0 | 0 | 0 | 0 | 0 |
| PER006 | 0 | 0 | 0 | 0 | 0 | 0 | 0 |
| PER007 | 0 | 0 | 0 | 0 | 0 | 0 | 0 |
| PER009 | 0 | 0 | 0 | 0 | 0.00301 | 0 | 0 |
| PER010 | 0 | 0 | 0 | 0 | 0 | 0 | 0 |
| PER011 | 0 | 0 | 0 | 0.00463 | 0 | 0 | 0 |
| PER012 | 0 | 0 | 0 | 0 | 0 | 0 | 0 |
| PER013 | 0 | 0 | 0 | 0 | 0 | 0 | 0 |
| PER014 | 0 | 0 | 0 | 0 | 0 | 0 | 0 |
| PER015 | 0 | 0 | 0 | 0 | 0 | 0 | 0 |
| PER016 | 0 | 0 | 0 | 0 | 0 | 0 | 0 |
| PER019 | 0 | 0 | 0 | 0 | 0 | 0 | 0 |
| PER020 | 0 | 0 | 0 | 0 | 0 | 0 | 0 |
| PER021 | 0 | 0 | 0 | 0 | 0 | 0 | 0 |
| PER022 | 0 | 0 | 0 | 0 | 0 | 0 | 0 |
| PER023 | 0 | 0 | 0 | 0 | 0 | 0.00382 | 0 |
| PER024 | 0 | 0 | 0 | 0 | 0 | 0 | 0 |
| PER025 | 0 | 0 | 0 | 0 | 0 | 0.00398 | 0 |
| PER026 | 0 | 0 | 0 | 0 | 0 | 0 | 0 |
| PER027 | 0 | 0 | 0 | 0 | 0 | 0 | 0 |
| SSC002 | 0 | 0 | 0 | 0 | 0 | 0 | 0 |
| SSC003 | 0 | 0.00708 | 0 | 0 | 0.14407 | 0.04835 | 0 |
| SSC004 | 0.06524 | 0.01615 | 0 | 0 | 0.46942 | 0.03084 | 0 |
| SSC005 | 0.07875 | 0.02183 | 0 | 0.17982 | 0.55238 | 0.22438 | 0.03817 |
| SSC006 | 0 | 0 | 0 | 0 | 0 | 0 | 0 |
| SSC007 | 0 | 0 | 0 | 0 | 0 | 0 | 0 |
| SSC008 | 0.08987 | 0.02322 | 0 | 0 | 0.71304 | 0.20767 | 0 |
| SSC009 | 0 | 0 | 0 | 0 | 0 | 0 | 0 |
| SSC011 | 0 | 0.00601 | 0 | 0.00682 | 0 | 0 | 0 |
| SSC012 | 0 | 0.01525 | 0 | 0 | 0 | 0 | 0 |
| SWC002 | 0 | 0 | 0 | 0 | 0 | 0 | 0 |
| SWC003 | 0 | 0 | 0 | 0 | 0 | 0 | 0 |
| SWC004 | 0 | 0 | 0 | 0 | 0.03877 | 0 | 0 |
| SWC005 | 0 | 0 | 0 | 0 | 0.09890 | 0 | 0 |
| SWC006 | 0 | 0.14617 | 0 | 0.03891 | 0.12134 | 0 | 0 |
| SWC007 | 0.00701 | 0 | 0 | 0 | 0.06304 | 0 | 0 |
| SWC008 | 0 | 0 | 0 | 0 | 0.05389 | 0 | 0 |
| SWC009 | 0 | 0 | 0 | 0 | 0.02983 | 0 | 0 |
| SWC010 | 0.01475 | 0.01108 | 0 | 0 | 0.15395 | 0 | 0 |
| SWC011 | 0.00587 | 0 | 0 | 0 | 0.02862 | 0 | 0 |
| SWC012 | 0 | 0.01222 | 0 | 0 | 0 | 0 | 0 |
| SWC013 | 0 | 0.01480 | 0 | 0 | 0.08547 | 0 | 0 |
| SWC014 | 0 | 0 | 0 | 0 | 0.04247 | 0 | 0 |

**Table S4.** Correspondence between culture-based and molecular-based microbial profiles in BALF. Cultures identified as “mixed oral flora” are not included in this table, as these microorganisms are not identified to the species level.

| BALF ID | Clinical microbiology (CFU) | OTU (% of reads) |
| --- | --- | --- |
| ADL001 | *Streptococcus viridans* (10^3^) | *Streptococcus* (2.37%) |
| ADL006 | *Haemophilus parainfluenzae* (10^5^) | *Haemophilus* (8.73%) |
| ADL006 | *Streptococcus viridans* (10^5^) | *Streptococcus* (20.94%) |
| ADL006 | *Neisseria* (10^5^) | *Neisseria* (9.98%) |
| ADL007 | Coagulase negative *Staphylococcus* (10^3^) | *Staphylococcus* (0.13%) |
| ADL007 | *Streptococcus viridans* (10^3^) | *Streptococcus* (10.29%) |
| BMC001 | *Pseudomonas aeruginosa* (10^6^) | *Pseudomonas* (1.91%) |
| BMC001 | *Staphylococcus aureus* (10^3^) | *Staphylococcus* (0.05%) |
| BMC001 | *Streptococcus* (10^8^) | *Streptococcus* (30.68%) |
| BMC001 | *Neisseria* (10^8^) | *Neisseria* (16.65%) |
| BMC003 | *Streptococcus* (10^7^) | *Streptococcus* (22.72%) |
| BMC003 | *Neisseria* (10^5^) | *Neisseria* (23.59%) |
| BMC004 | *Staphylococcus aureus* (10^3^) | *Staphylococcus* (5.33%) |
| BMC004 | *Haemophilus parainfluenzae* (10^3^) | *Haemophilus* (4.21%) |
| BMC004 | *Streptococcus* (10^4^) | *Streptococcus* (32.36%) |
| BMC004 | *Neisseria* (10^4^) | *Neisseria* (1.65%) |
| BMC005 | *Streptococcus* (10^4^); *Streptococcus viridans* (10^4^) | *Streptococcus* (48.97%) |
| BMC006 | *Staphylococcus aureus* (10^3^) | *Staphylococcus* (10.78%) |
| BMC006 | *Haemophilus influenzae* (10^3^) | *Haemophilus* (3.67%) |
| BMC006 | *Streptococcus* (10^4^) | *Streptococcus* (36.26%) |
| BMC007 | *Pseudomonas aeruginosa* (10^3^) | *Pseudomonas* (0.04%) |
| BMC007 | *Klebsiella oxytoca* (10^3^) | No detected |
| BMC007 | *Streptococcus viridans* (10^7^) | *Streptococcus* (25.15%) |
| BMC007 | *Neisseria* (10^7^) | *Neisseria* (10.33%) |
| BRC001 | *Streptococcus* (10^3^) | *Streptococcus* (36.71%) |
| BRC001 | *Neisseria* (10^3^) | *Neisseria* (5.03%) |
| BRC002 | *Pseudomonas aeruginosa* (10^3^) | *Pseudomonas* (0.39%) |
| BRC002 | *Escherichia coli* (10^3^) | *Escherichia-Shigella* (0.06%) |
| BRC002 | *Klebsiella oxytoca* (10^3^) | No detected |
| BRC002 | Coagulase negative *Staphylococcus* (10^3^) | *Staphylococcus* (0.15%) |
| BRC002 | *Streptococcus* (10^4^) | *Streptococcus* (25.44%) |
| BRC003 | *Escherichia coli* (10^3^) | *Escherichia-Shigella* (0.06%) |
| BRC003 | *Streptococcus* (10^6^) | *Streptococcus* (32.61%) |
| BRC003 | *Neisseria* (10^4^) | *Neisseria* (10.56%) |
| BRC003 | *Corynebacterium* (10^6^) | *Corynebacterium* (0.05%) |
| BRC004 | *Staphylococcus* (10^3^) | *Staphylococcus* (2.12%) |
| BRC004 | *Streptococcus* (10^3^) | *Streptococcus* (1.18%) |
| BRC006 | *Streptococcus* (10^6^); *Streptococcus viridans* (10^6^) | *Streptococcus* (42.14%) |
| BRC006 | *Neisseria* (10^3^) | *Neisseria* (0.83%) |
| BRC007 | *Staphylococcus aureus* (10^4^) | *Staphylococcus* (1.82%) |
| BRC007 | *Haemophilus* (10^6^) | *Haemophilus* (5.66%) |
| BRC007 | *Streptococcus viridans* (10^6^) | *Streptococcus* (21.64%) |
| BRC007 | *Neisseria* (10^6^) | *Neisseria* (21.88%) |
| BRC008 | *Haemophilus influenzae* (10^8^) | *Haemophilus* (13.31%) |
| BRC008 | Coagulase negative *Staphylococcus* (10^6^) | *Staphylococcus* (0.1%) |
| BRC008 | *Streptococcus viridans* (10^8^) | *Streptococcus* (26.10%) |
| BRC008 | *Neisseria* (10^6^) | *Neisseria* (22.68%) |
| LCC001 | *Staphylococcus aureus* (10^4^) | *Staphylococcus* (1.63%) |
| LCC002 | *Staphylococcus aureus* (10^3^) | *Staphylococcus* (0.07%) |
| LCC002 | *Haemophilus influenzae* (10^6^) | *Haemophilus* (1.97%) |
| LCC002 | *Streptococcus viridans* (10^7^) | *Streptococcus* (17.19%) |
| LCC002 | *Neisseria* (10^8^) | *Neisseria* (26.96%) |
| LCC003 | *Haemophilus influenzae* (10^6^) | *Haemophilus* (21.25%) |
| LCC003 | *Streptococcus viridans* (10^6^) | *Streptococcus* (12.39%) |
| LCC003 | *Neisseria* (10^6^) | *Neisseria* (20.80%) |
| LCC004 | *Haemophilus influenzae* (10^7^) | *Haemophilus* (55.84%) |
| LCC004 | *Escherichia coli* (10^3^) | *Escherichia-Shigella* (0.09%) |
| LCC004 | *Streptococcus viridans* (10^6^) | *Streptococcus* (17.54%) |
| LCC005 | *Staphylococcus aureus* (10^3^); Coagulase negative *Staphylococcus* (10^5^) | *Staphylococcus* (0.32%) |
| LCC005 | *Haemophilus* (10^5^) | *Haemophilus* (10.77%) |
| LCC005 | *Streptococcus* (10^5^); *Streptococcus viridans* (10^5^) | *Streptococcus* (28.86%) |
| LCC005 | *Neisseria* (10^5^) | *Neisseria* (11.82%) |
| LCC006 | *Pseudomonas aeruginosa* (10^8^) | *Pseudomonas* (4.56%) |
| LCC007 | *Pseudomonas aeruginosa* (10^6^) | *Pseudomonas* (9.54%) |
| LCC007 | *Streptococcus viridans* (10^4^) | *Streptococcus* (27.70%) |
| LCC009 | *Streptococcus viridans* (10^6^) | *Streptococcus* (53.55%) |
| MMC002 | *Staphylococcus aureus* (10^5^) | *Staphylococcus* (1.36%) |
| MRC005 | *Stenotrophomonas maltophilia* (10^7^) | *Stenotrophomonas* (99.76%) |
| MRC005 | *Aspergillus fumigatus* (10^5^) | No ITS2 amplicon |
| MRC005 | *Propionibacterium acnes* (10^5^) | No detected |
| MRC006 | *Haemophilus parainfluenzae* (10^7^) | *Haemophilus* (4.38%) |
| MRC006 | *Citrobacter freundii* (10^5^) | No detected |
| MRC008 | *Candida albicans* (10^5^) | No ITS2 amplicon |
| MRC010 | *Candida albicans* (10^5^) | No ITS2 amplicon |
| MRC016 | *Escherichia coli* (10^7^) | *Escherichia-Shigella* (0.55%) |
| MRC016 | *Candida albicans* (10^5^) | No ITS2 amplicon |
| MRC020 | *Haemophilus parainfluenzae* (10^5^) | *Haemophilus* (0.05%) |
| MRC023 | *Escherichia coli* (10^6^) | No detected |
| MRC023 | *Enterobacter cloacae* (10^6^) | *Enterobacter* (0.56%) |
| MRC025 | *Pseudomonas aeruginosa* (10^6^) | *Pseudomonas* (4.30%) |
| NZA002 | *Staphylococcus aureus* (10^8^) | *Staphylococcus* (49.14%) |
| NZA002 | *Escherichia coli* (10^8^) | *Escherichia-Shigella* (2.27%) |
| NZA003 | *Staphylococcus aureus* (10^8^) | *Staphylococcus* (10%) |
| NZA005 | *Escherichia coli* (10^8^) | *Escherichia-Shigella* (1.95%) |
| NZA008 | *Staphylococcus aureus* (10^4^) | *Staphylococcus* (0.04%) |
| NZA008 | *Haemophilus influenzae* (10^10^) | *Haemophilus* (5.84%) |
| NZA009 | *Streptococcus pneumoniae* (10^8^) | *Streptococcus* (35.26%) |
| NZA010 | *Streptococcus pneumoniae* (10^8^) | *Streptococcus* (33.05%) |
| NZA011 | *Haemophilus influenzae* (10^4^) | *Haemophilus* (20.11%) |
| NZA012 | *Klebsiella oxytoca* (10^8^) | No detected |
| NZA012 | *Aspergillus* (10^3^) | *Aspergillus* (0.42%) |
| NZA016 | *Serratia marescens* (10^9^) | *Serratia* (0.003%) |
| NZA020 | *Aspergillus fumigatus* (10^3^) | *Aspergillus* (25.33%) |
| SSC002 | *Haemophilus influenzae* (10^4^) | *Haemophilus* (7.04%) |
| SSC006 | *Streptococcus pneumoniae* (10^2^) | *Streptococcus* (1.78%) |
| SSC011 | *Candida albicans* (10^4^) | *Candida albicans* (9.06%) |
| SSC011 | *Aspergillus fumigatus* (10) | *Aspergillus* (1.39%) |
| SWC004 | *Haemophilus influenzae* (10^6^) | *Haemophilus* (28.12%) |
| SWC006 | *Haemophilus influenzae* (10^6^) | *Haemophilus* (5.70%) |
| SWC007 | *Escherichia coli* (10^3^) | *Escherichia-Shigella* (0.14%) |
| SWC010 | *Haemophilus influenzae* (10^6^) | *Haemophilus* (10.51%) |
| SWC012 | Methicillin resistant *Staphylococcus aureus* (10^7^) | *Staphylococcus* (91.29%) |
| SWC012 | *Haemophilus influenzae* (10^7^) | *Haemophilus* (2.58%) |
| SWC013 | *Moraxella catharralis* (10^8^) | *Moraxella* (34.38%) |
| SWC013 | *Escherichia coli* (10^4^) | *Escherichia-Shigella* (0.08%) |
| PER002 | *Klebsiella oxytoca* (10^5^) | No detected |
| PER004 | *Candida albicans* (10^3^) | No ITS2 amplicon |
| PER009 | *Escherichia coli* (10^5^) | *Escherichia-Shigella* (0.22%) |
| PER009 | *Klebsiella oxytoca* (10^6^) | No detected |
| PER011 | *Sphingomonas paucimobilis* (10^5^) | No detected |
| PER016 | *Pseudomonas aeruginosa* (10^7^) | *Pseudomonas* (84.91%) |
| PER020 | *Streptococcus pneumoniae* (10^5^) | *Streptococcus* (11.39%) |
| PER021 | *Haemophilus influenzae* (10^7^) | No detected |

**Table S5.** Descriptive statistics for the bacterial load observed in negative extraction controls, bronchoscope washes and BALF specimens. The negative extraction controls were used as reference group when comparing the bacterial load between sample types.

| Sample type | Mean (DNA copies µL^-1^ of DNA extract) [Standard deviation] | Median (DNA copies µL^-1^  of DNA extract) [Interquartile range] | Wilcoxon test with Bonferroni correction |
| --- | --- | --- | --- |
| Negative extraction controls | 787.53 [205.07] | 767.60 [653.94-881.22] | - |
| Bronchoscope washes | 759.55 [208.19] | 828.60 [552.40-828.60] | *p*>0.05 |
| BALF specimens | 118,615.10 [392,243.60] | 19,057.79 [6,352.59-66,521.57] | *p*<0.0001 |
| BALF specimens (only samples with more than 1,400 DNA copies µL^-1^ of DNA extract) | 127,949.70 [406,234.50] | 24,669.09 [8,285.99-68,569.88] | *p*<0.0001 |

**Table S6.** This table contains information in regards to the relative abundance (mean and standard deviation) and prevalence of the top 11 fungal ASVs obtained from the BALF DNA extracts. The profile contains fungi associated with the nasal (*Aureobasidium pullulans*, *Alternaria*, *Candida albicans*) and oral (*Malassezia*, *Alternaria*, *Candida*, *Aspergillus*, *Filobasidium*, *Aureobasidium* and *Cryptococcus* species) cavities [[3-5](#_ENREF_3)], skin commensals (*Malassezia restricta*, *Malassezia globosa*, *Candida parasilopsis*, *Alternaria*, *Rhodotorula*, *Cryptococcus*, *Aspergillus*) [[6](#_ENREF_6)], and fungi described in the respiratory tract of CF patients (*Malassezia restricta*, *Malassezia globosa* and *Candida albicans*) [[7](#_ENREF_7),[8](#_ENREF_8)]. Interestingly, many of the observed species in the fungal profile have been also described at high prevalence in the indoor environment, and have been proposed to colonize the lungs through environmental exposure [[9](#_ENREF_9)]. *, Formerly known as *Cryptococcus albidus*; **, *Meyerozyma guilliermondii* is the teleomorph specie of *Candida guilliermondii*.

| ASVs | Prevalence: Number of positive samples (%) | Mean relative abundance | Standard deviation |
| --- | --- | --- | --- |
| *Filobasidium* | 31 (83.78%) | 17.02 | 14.74 |
| *Naginishia albida ** | 31 (83.78%) | 13.85 | 11.81 |
| *Aureobasidium pullulans* | 27 (72.97%) | 6.99 | 8.52 |
| *Candida albicans* | 13 (35.13%) | 6.34 | 19.22 |
| *Trichoderma lixii* | 37 (100%) | 5.65 | 4.74 |
| *Malassezia restricta* | 35 (94.59%) | 5.64 | 4.99 |
| *Rhodoturula mucilaginosa* | 31 (83.78%) | 4.93 | 4.86 |
| *Meyerozyma guillermondii *** | 33 (89.19%) | 4.65 | 6.16 |
| *Aspergillus* | 15 (40.54%) | 1.93 | 6.14 |
| *Candida parapsilosis* | 27 (72.97%) | 1.78 | 2.53 |
| *Malassezia arunalokei* | 28 (75.67%) | 1.24 | 1.61 |
| *Malassezia globosa* | 27 (72.97%) | 1.21 | 1.62 |
| *Alternaria* | 14 (37.84%) | 1.74 | 6.77 |

**Table S7.** Absolute read counts for the OTUs detected as contaminants by the R package *decontam* [[10](#_ENREF_10)]. BALF specimens are identified by an alphanumeric code in which the three first letters identify the Centre (PER, BRC, BMC, NZA, SWC, SSC, MRC, MMC, ADL, LCC). Negative extraction controls are identified with “CNT”, while bronchoscope washes are named with “WASH” followed by the code of the paired BALF specimen.

| OTU | Bacteria;Actinobacteriota;  Actinobacteria;  Micrococcales;Micrococcaceae;  Renibacterium; | Bacteria;Proteobacteria;  Alphaproteobacteria;  Rhizobiales;Rhizobiaceae;  Brucella; | Bacteria;Proteobacteria;  Alphaproteobacteria;  Sphingomonadales;  Sphingomonadaceae;  Sphingomonas; | Bacteria;Proteobacteria;Gammaproteobacteria;  Pseudomonadales;  Moraxellaceae;Acinetobacter; |
| --- | --- | --- | --- | --- |
| ADL001 | 3 | 280 | 0 | 534 |
| ADL003 | 24 | 66 | 0 | 309 |
| ADL005 | 0 | 351 | 0 | 959 |
| ADL006 | 17 | 156 | 63 | 863 |
| ADL007 | 0 | 26 | 6 | 178 |
| BMC001 | 0 | 13 | 0 | 16 |
| BMC002 | 21 | 65 | 6 | 717 |
| BMC003 | 3 | 2 | 0 | 17 |
| BMC004 | 3 | 39 | 0 | 102 |
| BMC005 | 11 | 37 | 0 | 180 |
| BMC006 | 0 | 0 | 0 | 6 |
| BMC007 | 0 | 0 | 0 | 0 |
| BRC001 | 0 | 0 | 0 | 11 |
| BRC002 | 0 | 14 | 0 | 128 |
| BRC003 | 8 | 4 | 11 | 66 |
| BRC004 | 0 | 22 | 3 | 94 |
| BRC006 | 0 | 2 | 2 | 15 |
| BRC007 | 0 | 0 | 0 | 17 |
| BRC008 | 0 | 62 | 9 | 310 |
| LCC001 | 0 | 4 | 16 | 33 |
| LCC002 | 0 | 0 | 0 | 3 |
| LCC003 | 0 | 20 | 0 | 8 |
| LCC004 | 0 | 0 | 0 | 2 |
| LCC005 | 0 | 0 | 0 | 0 |
| LCC006 | 0 | 0 | 0 | 2 |
| LCC007 | 0 | 0 | 0 | 0 |
| LCC009 | 2 | 0 | 0 | 37 |
| MMC002 | 0 | 5 | 6 | 7 |
| MRC002 | 56 | 173 | 0 | 656 |
| MRC003 | 0 | 122 | 5 | 1383 |
| MRC004 | 2 | 13 | 0 | 104 |
| MRC005 | 0 | 0 | 0 | 18 |
| MRC006 | 0 | 0 | 0 | 3 |
| MRC007 | 2 | 6 | 2 | 46 |
| MRC008 | 0 | 7 | 3 | 33 |
| MRC009 | 11 | 12 | 14 | 163 |
| MRC010 | 16 | 43 | 0 | 956 |
| MRC011 | 0 | 327 | 129 | 1841 |
| MRC012 | 0 | 245 | 44 | 628 |
| MRC013 | 17 | 158 | 53 | 876 |
| MRC015 | 23 | 296 | 206 | 1882 |
| MRC016 | 0 | 21 | 46 | 217 |
| MRC017 | 0 | 171 | 0 | 731 |
| MRC018 | 0 | 0 | 2 | 23 |
| MRC019 | 0 | 165 | 55 | 574 |
| MRC020 | 0 | 8 | 14 | 63 |
| MRC021 | 21 | 72 | 170 | 433 |
| MRC022 | 0 | 0 | 56 | 56 |
| MRC023 | 22 | 42 | 68 | 517 |
| MRC025 | 7 | 11 | 59 | 183 |
| MRC026 | 0 | 0 | 4 | 28 |
| MRC028 | 22 | 37 | 39 | 788 |
| NZA001 | 0 | 0 | 0 | 0 |
| NZA002 | 0 | 0 | 0 | 4 |
| NZA003 | 0 | 0 | 0 | 2 |
| NZA004 | 0 | 0 | 2 | 2 |
| NZA005 | 0 | 2 | 2 | 19 |
| NZA006 | 0 | 0 | 0 | 6 |
| NZA007 | 0 | 0 | 0 | 0 |
| NZA008 | 0 | 3 | 5 | 9 |
| NZA009 | 11 | 48 | 13 | 313 |
| NZA010 | 0 | 0 | 0 | 0 |
| NZA011 | 0 | 45 | 0 | 88 |
| NZA012 | 0 | 0 | 3 | 6 |
| NZA014 | 0 | 17 | 0 | 291 |
| NZA015 | 0 | 4 | 2 | 28 |
| NZA016 | 0 | 0 | 0 | 3 |
| NZA017 | 0 | 0 | 0 | 2 |
| NZA018 | 0 | 0 | 0 | 4 |
| NZA020 | 0 | 0 | 0 | 0 |
| PER001 | 22 | 35 | 54 | 548 |
| PER002 | 0 | 0 | 3 | 104 |
| PER003 | 0 | 80 | 11 | 291 |
| PER004 | 0 | 16 | 11 | 143 |
| PER005 | 0 | 46 | 29 | 270 |
| PER006 | 0 | 114 | 1078 | 558 |
| PER009 | 0 | 0 | 6 | 19 |
| PER010 | 0 | 726 | 0 | 1289 |
| PER011 | 0 | 4 | 12 | 39 |
| PER012 | 0 | 37 | 82 | 334 |
| PER013 | 0 | 3 | 4 | 141 |
| PER014 | 0 | 70 | 0 | 395 |
| PER015 | 0 | 7 | 4 | 71 |
| PER016 | 0 | 0 | 0 | 6 |
| PER019 | 0 | 27 | 0 | 927 |
| PER020 | 5 | 34 | 0 | 443 |
| PER021 | 7 | 281 | 14 | 1028 |
| PER022 | 4 | 13 | 10 | 667 |
| PER024 | 0 | 149 | 7 | 534 |
| PER025 | 0 | 21 | 7 | 100 |
| PER026 | 5 | 48 | 14 | 309 |
| PER027 | 7 | 32 | 0 | 183 |
| SSC002 | 14 | 221 | 111 | 995 |
| SSC003 | 12 | 3656 | 0 | 1147 |
| SSC004 | 5 | 22 | 40 | 278 |
| SSC006 | 53 | 121 | 0 | 660 |
| SSC007 | 0 | 2 | 6 | 47 |
| SSC009 | 0 | 13 | 10 | 1169 |
| SSC011 | 50 | 163 | 57 | 745 |
| SWC002 | 13 | 31 | 10 | 281 |
| SWC003 | 0 | 163 | 9 | 1190 |
| SWC004 | 2 | 21 | 13 | 99 |
| SWC005 | 0 | 14 | 0 | 44 |
| SWC006 | 0 | 0 | 2 | 4 |
| SWC007 | 2 | 7 | 3 | 19 |
| SWC008 | 0 | 3 | 0 | 13 |
| SWC009 | 0 | 26 | 9 | 86 |
| SWC010 | 0 | 7 | 2 | 35 |
| SWC012 | 0 | 0 | 0 | 0 |
| SWC013 | 0 | 0 | 0 | 3 |
| SWC014 | 0 | 2 | 0 | 7 |
| CNT113 | 0 | 198 | 59 | 1525 |
| CNT13 | 0 | 97 | 41 | 453 |
| CNT192 | 0 | 25 | 5 | 269 |
| CNT262 | 9 | 43 | 7 | 452 |
| CNT272 | 2 | 23 | 3 | 204 |
| CNT63 | 11 | 130 | 9 | 1050 |
| CNT83 | 35 | 104 | 39 | 594 |
| CNTN | 11 | 11 | 0 | 518 |
| WASH_PER003 | 21 | 244 | 30 | 704 |
| WASH_PER007 | 22 | 96 | 9 | 361 |
| WASH_PER009 | 9 | 228 | 36 | 497 |
| WASH_PER012 | 5 | 66 | 60 | 849 |
| WASH_PER014 | 18 | 224 | 137 | 397 |
| WASH_PER015 | 12 | 79 | 7 | 610 |
| WASH_PER016 | 5 | 79 | 0 | 386 |
| WASH_PER019 | 16 | 37 | 13 | 844 |
| WASH_PER021 | 0 | 111 | 164 | 618 |
| WASH_PER023 | 17 | 140 | 23 | 564 |
| WASH_PER025 | 16 | 35 | 6 | 382 |
| WASH_PER026 | 0 | 48 | 0 | 537 |

**Table S8.** Results of the permutational test of significance of the Procrustes analyses (using the function *protest* implemented in the R package *vegan* [[11](#_ENREF_11)]). The great concordance shown between the compared datasets (low M^2^ and high correlation coefficient) suggests that the filtering steps did not remarkably impact the composition of the original microbial profiles (Raw counts).

| Datasets | Procrustes Sum of Squares (M^2^) | Correlation in a symmetric Procrustes rotation | Significance |
| --- | --- | --- | --- |
| Raw counts Vs Removed low counts/singletones/Unclassified/Mithochondria/  Chloroplast | 0.0127 | 0.9936 | 0.0001 |
| Raw counts Vs Removed low counts/singletones/Unclassified Mithochondria/  Chloroplast and contaminants | 0.0305 | 0.9846 | 0.0001 |

**Table S9.** Absolute read counts for the ASVs detected as contaminants by the R package *decontam* in the ITS2-based fungal profiles [[10](#_ENREF_10)]. BALF specimens are identified by an alphanumeric code in which the three first letters identify the Centre (BRC, BMC, NZA, SWC, SSC, MRC, LCC). Negative extraction controls are identified with “CNT”, while bronchoscope washes are named with “WASH” followed by the code of the paired BALF specimen. For simplification ASVs are indicated with numbers and identified in Supplemental Table 9.

| ASVs | 1 | 2 | 3 | 4 | 5 | 6 | 7 | 8 | 9 | 10 | 11 | 12 | 13 | 14 | 15 | 16 | 17 | 18 | 19 | 20 | 21 | 22 | 23 | 24 |
| --- | --- | --- | --- | --- | --- | --- | --- | --- | --- | --- | --- | --- | --- | --- | --- | --- | --- | --- | --- | --- | --- | --- | --- | --- |
| BMC006 | 7 | 0 | 0 | 0 | 272 | 438 | 265 | 0 | 0 | 0 | 0 | 0 | 0 | 0 | 0 | 0 | 0 | 0 | 0 | 0 | 0 | 1734 | 0 | 0 |
| BMC007 | 0 | 0 | 0 | 0 | 0 | 577 | 0 | 0 | 0 | 156 | 0 | 15 | 0 | 0 | 0 | 0 | 17 | 0 | 0 | 0 | 0 | 0 | 0 | 0 |
| BRC001 | 0 | 0 | 0 | 0 | 192 | 500 | 0 | 0 | 0 | 311 | 0 | 0 | 0 | 0 | 0 | 0 | 0 | 0 | 0 | 0 | 0 | 0 | 0 | 0 |
| BRC002 | 2345 | 1833 | 7416 | 0 | 0 | 0 | 0 | 0 | 0 | 0 | 0 | 0 | 0 | 0 | 0 | 0 | 0 | 202 | 0 | 0 | 0 | 0 | 0 | 0 |
| BRC007 | 722 | 146 | 0 | 0 | 0 | 0 | 0 | 0 | 0 | 0 | 0 | 4241 | 0 | 0 | 0 | 0 | 3336 | 0 | 0 | 0 | 0 | 0 | 0 | 0 |
| LCC001 | 0 | 0 | 0 | 0 | 0 | 0 | 0 | 0 | 0 | 0 | 0 | 0 | 0 | 0 | 0 | 0 | 0 | 0 | 0 | 0 | 0 | 0 | 0 | 0 |
| LCC002 | 0 | 375 | 0 | 0 | 0 | 0 | 0 | 0 | 0 | 0 | 0 | 0 | 0 | 0 | 0 | 0 | 0 | 0 | 0 | 0 | 0 | 0 | 0 | 0 |
| LCC003 | 0 | 64 | 0 | 0 | 0 | 0 | 0 | 0 | 0 | 195 | 0 | 0 | 214 | 0 | 0 | 0 | 0 | 0 | 0 | 0 | 0 | 0 | 0 | 0 |
| LCC004 | 369 | 0 | 0 | 0 | 0 | 0 | 0 | 0 | 0 | 0 | 0 | 0 | 0 | 0 | 0 | 0 | 0 | 0 | 0 | 0 | 0 | 0 | 0 | 0 |
| LCC005 | 224 | 75 | 0 | 0 | 0 | 0 | 0 | 0 | 0 | 0 | 0 | 0 | 0 | 0 | 0 | 0 | 0 | 0 | 0 | 0 | 0 | 0 | 0 | 0 |
| MRC018 | 435 | 0 | 0 | 52 | 0 | 0 | 0 | 0 | 0 | 205 | 0 | 0 | 0 | 0 | 0 | 197 | 0 | 0 | 0 | 0 | 0 | 0 | 0 | 0 |
| MRC020 | 0 | 0 | 0 | 0 | 0 | 0 | 0 | 0 | 0 | 0 | 0 | 0 | 0 | 0 | 160 | 0 | 0 | 0 | 0 | 0 | 0 | 0 | 0 | 0 |
| MRC021 | 0 | 0 | 0 | 76 | 0 | 286 | 0 | 309 | 0 | 0 | 0 | 0 | 0 | 0 | 0 | 0 | 0 | 0 | 0 | 0 | 0 | 0 | 0 | 0 |
| MRC025 | 0 | 0 | 0 | 1515 | 0 | 0 | 0 | 236 | 0 | 0 | 0 | 0 | 51 | 0 | 0 | 0 | 0 | 0 | 0 | 0 | 0 | 0 | 0 | 0 |
| MRC026 | 0 | 0 | 0 | 0 | 0 | 0 | 0 | 106 | 0 | 0 | 0 | 0 | 0 | 0 | 0 | 0 | 0 | 0 | 0 | 0 | 0 | 0 | 0 | 0 |
| NZA001 | 0 | 103 | 0 | 0 | 0 | 0 | 0 | 512 | 0 | 0 | 0 | 0 | 113 | 0 | 0 | 0 | 0 | 0 | 0 | 0 | 0 | 0 | 0 | 0 |
| NZA002 | 0 | 319 | 0 | 0 | 0 | 0 | 0 | 1336 | 0 | 0 | 0 | 0 | 0 | 0 | 0 | 0 | 0 | 0 | 0 | 0 | 0 | 0 | 0 | 0 |
| NZA003 | 649 | 3 | 0 | 0 | 0 | 95 | 0 | 0 | 0 | 0 | 0 | 0 | 0 | 0 | 0 | 0 | 0 | 0 | 0 | 0 | 0 | 0 | 0 | 0 |
| NZA004 | 0 | 0 | 0 | 0 | 0 | 0 | 0 | 1160 | 0 | 0 | 0 | 0 | 0 | 0 | 0 | 0 | 0 | 0 | 0 | 0 | 0 | 0 | 0 | 0 |
| NZA006 | 0 | 0 | 0 | 0 | 0 | 0 | 0 | 163 | 0 | 0 | 0 | 0 | 0 | 0 | 0 | 0 | 0 | 0 | 0 | 0 | 0 | 0 | 0 | 0 |
| NZA007 | 2079 | 2140 | 8089 | 57 | 0 | 83 | 0 | 0 | 0 | 0 | 0 | 0 | 0 | 0 | 0 | 0 | 0 | 660 | 0 | 0 | 0 | 0 | 0 | 0 |
| NZA008 | 0 | 0 | 0 | 0 | 0 | 0 | 0 | 0 | 0 | 0 | 0 | 0 | 856 | 0 | 0 | 0 | 0 | 0 | 0 | 0 | 0 | 0 | 0 | 0 |
| NZA009 | 0 | 0 | 0 | 0 | 0 | 0 | 0 | 0 | 0 | 0 | 0 | 0 | 875 | 0 | 0 | 0 | 0 | 0 | 0 | 0 | 0 | 0 | 0 | 0 |
| NZA010 | 0 | 199 | 0 | 0 | 0 | 0 | 0 | 141 | 0 | 0 | 0 | 0 | 1918 | 0 | 0 | 71 | 0 | 0 | 0 | 0 | 0 | 0 | 0 | 0 |
| NZA011 | 0 | 199 | 0 | 0 | 213 | 0 | 0 | 0 | 0 | 0 | 0 | 0 | 0 | 0 | 0 | 0 | 0 | 0 | 0 | 0 | 0 | 0 | 0 | 0 |
| NZA012 | 0 | 0 | 30 | 0 | 0 | 0 | 0 | 211 | 0 | 0 | 0 | 0 | 0 | 0 | 0 | 0 | 0 | 0 | 0 | 0 | 0 | 0 | 0 | 0 |
| NZA015 | 0 | 0 | 0 | 0 | 0 | 262 | 0 | 299 | 0 | 0 | 0 | 0 | 0 | 0 | 0 | 0 | 0 | 0 | 0 | 0 | 0 | 0 | 0 | 0 |
| NZA016 | 0 | 0 | 0 | 0 | 0 | 0 | 0 | 0 | 0 | 0 | 0 | 0 | 0 | 0 | 0 | 0 | 0 | 0 | 0 | 0 | 0 | 0 | 0 | 0 |
| NZA017 | 0 | 0 | 0 | 85 | 0 | 0 | 0 | 0 | 0 | 0 | 0 | 0 | 0 | 0 | 0 | 0 | 0 | 0 | 0 | 0 | 0 | 0 | 0 | 0 |
| NZA018 | 0 | 0 | 0 | 0 | 0 | 0 | 0 | 0 | 0 | 0 | 0 | 0 | 0 | 0 | 0 | 0 | 0 | 0 | 0 | 0 | 0 | 0 | 0 | 0 |
| NZA020 | 239 | 180 | 0 | 0 | 0 | 0 | 0 | 0 | 0 | 0 | 0 | 0 | 0 | 0 | 0 | 0 | 0 | 0 | 0 | 0 | 0 | 0 | 0 | 0 |
| SSC011 | 92 | 0 | 0 | 0 | 0 | 0 | 0 | 0 | 0 | 0 | 0 | 0 | 0 | 0 | 0 | 0 | 0 | 0 | 0 | 0 | 0 | 0 | 0 | 0 |
| SSC012 | 0 | 0 | 0 | 0 | 0 | 0 | 0 | 966 | 0 | 0 | 0 | 0 | 0 | 0 | 35 | 0 | 0 | 0 | 0 | 0 | 0 | 0 | 0 | 0 |
| SWC006 | 265 | 0 | 0 | 0 | 0 | 0 | 0 | 779 | 0 | 0 | 0 | 0 | 0 | 0 | 209 | 0 | 0 | 0 | 0 | 0 | 0 | 0 | 0 | 0 |
| SWC009 | 0 | 0 | 0 | 0 | 0 | 0 | 0 | 0 | 0 | 0 | 0 | 0 | 0 | 0 | 0 | 0 | 0 | 0 | 0 | 0 | 0 | 0 | 0 | 0 |
| SWC010 | 0 | 0 | 0 | 0 | 0 | 470 | 0 | 0 | 0 | 0 | 151 | 0 | 0 | 0 | 0 | 0 | 0 | 0 | 0 | 0 | 0 | 0 | 0 | 0 |
| SWC014 | 0 | 0 | 0 | 0 | 0 | 0 | 0 | 0 | 0 | 236 | 0 | 0 | 0 | 0 | 0 | 0 | 0 | 0 | 0 | 0 | 0 | 0 | 0 | 0 |
| CNT113 | 0 | 0 | 0 | 0 | 0 | 0 | 0 | 0 | 0 | 0 | 0 | 0 | 0 | 0 | 0 | 0 | 0 | 0 | 0 | 0 | 0 | 0 | 0 | 0 |
| CNT13 | 0 | 0 | 0 | 0 | 919 | 0 | 1702 | 0 | 0 | 0 | 0 | 0 | 0 | 0 | 0 | 0 | 0 | 0 | 0 | 0 | 0 | 0 | 0 | 0 |
| CNT192 | 1787 | 587 | 0 | 0 | 882 | 0 | 0 | 0 | 0 | 0 | 0 | 0 | 0 | 0 | 0 | 0 | 0 | 0 | 0 | 0 | 0 | 0 | 0 | 0 |
| CNT262 | 0 | 0 | 0 | 0 | 0 | 0 | 0 | 0 | 0 | 0 | 0 | 0 | 0 | 0 | 0 | 0 | 0 | 0 | 0 | 0 | 0 | 0 | 0 | 0 |
| CNT272 | 1484 | 0 | 0 | 0 | 0 | 0 | 0 | 0 | 0 | 0 | 0 | 0 | 0 | 0 | 0 | 0 | 0 | 0 | 0 | 0 | 0 | 0 | 0 | 0 |
| CNT63 | 0 | 2362 | 0 | 0 | 0 | 0 | 0 | 0 | 0 | 0 | 0 | 0 | 0 | 0 | 0 | 0 | 0 | 0 | 0 | 1081 | 1829 | 0 | 0 | 0 |
| CNT83 | 1320 | 0 | 0 | 0 | 0 | 0 | 0 | 0 | 0 | 1044 | 0 | 0 | 0 | 0 | 0 | 0 | 0 | 0 | 0 | 0 | 0 | 0 | 0 | 0 |
| WASH_PER003 | 0 | 0 | 0 | 0 | 1928 | 0 | 0 | 0 | 0 | 0 | 1798 | 0 | 0 | 0 | 0 | 0 | 0 | 0 | 0 | 0 | 0 | 0 | 0 | 0 |
| WASH_PER007 | 0 | 0 | 0 | 0 | 0 | 0 | 0 | 0 | 0 | 0 | 0 | 0 | 0 | 0 | 0 | 0 | 0 | 0 | 0 | 0 | 0 | 0 | 0 | 0 |
| WASH_PER009 | 0 | 0 | 0 | 0 | 0 | 0 | 0 | 0 | 0 | 0 | 0 | 0 | 0 | 0 | 0 | 0 | 0 | 0 | 0 | 0 | 0 | 0 | 0 | 0 |
| WASH_PER012 | 0 | 0 | 0 | 0 | 0 | 0 | 0 | 0 | 0 | 0 | 0 | 0 | 0 | 0 | 0 | 0 | 0 | 0 | 0 | 0 | 0 | 0 | 0 | 0 |
| WASH_PER014 | 725 | 0 | 0 | 0 | 0 | 0 | 0 | 0 | 0 | 0 | 0 | 0 | 0 | 0 | 0 | 0 | 0 | 0 | 0 | 0 | 0 | 0 | 0 | 0 |
| WASH_PER015 | 885 | 0 | 0 | 0 | 0 | 0 | 0 | 0 | 0 | 0 | 0 | 0 | 0 | 0 | 0 | 0 | 0 | 0 | 0 | 0 | 0 | 0 | 0 | 1498 |
| WASH_PER016 | 0 | 0 | 0 | 0 | 0 | 0 | 0 | 0 | 0 | 0 | 0 | 0 | 0 | 0 | 0 | 0 | 0 | 0 | 0 | 0 | 0 | 0 | 0 | 0 |
| WASH_PER019 | 0 | 0 | 0 | 0 | 0 | 0 | 0 | 0 | 0 | 0 | 0 | 0 | 0 | 0 | 0 | 0 | 0 | 0 | 0 | 0 | 0 | 0 | 0 | 0 |
| WASH_PER021 | 0 | 0 | 0 | 0 | 0 | 0 | 0 | 0 | 0 | 0 | 0 | 0 | 0 | 0 | 0 | 0 | 0 | 0 | 0 | 0 | 0 | 0 | 0 | 0 |
| WASH_PER023 | 414 | 0 | 0 | 0 | 0 | 0 | 0 | 0 | 0 | 1327 | 0 | 0 | 0 | 0 | 0 | 0 | 0 | 0 | 0 | 0 | 0 | 0 | 0 | 0 |
| WASH_PER025 | 0 | 0 | 0 | 0 | 0 | 0 | 0 | 0 | 0 | 0 | 0 | 0 | 0 | 0 | 0 | 0 | 0 | 0 | 0 | 0 | 0 | 0 | 0 | 0 |
| WASH_PER026 | 0 | 0 | 0 | 0 | 0 | 0 | 0 | 0 | 0 | 0 | 0 | 0 | 0 | 0 | 0 | 0 | 0 | 0 | 0 | 0 | 0 | 0 | 0 | 0 |

**Table S10.** Biological classification of the ASVs identified as potential contaminants shown in Supplemental Table 8. Eleven different ASVs were assigned to the *Malassezia restricta* species rank, of which one was identified as potential contaminants. Anagously, eight ASVs were classified as *Aspergillus*, being two of them was predicted as a putative contaminant.

|  | Kingdom | Phylum | Class | Order | Family | Genus | Species | ASV |
| --- | --- | --- | --- | --- | --- | --- | --- | --- |
| 1 | Fungi | Ascomycota | Sordariomycetes | Hypocreales | Nectriaceae | Fusarium | oxysporum | 1 |
| 2 | Fungi | Basidiomycota | Malasseziomycetes | Malasseziales | Malasseziaceae | Malassezia | restricta | 5 |
| 3 | Fungi | Ascomycota | Sordariomycetes | Hypocreales | Nectriaceae | Fusarium | oxysporum | 2 |
| 4 | Fungi | Ascomycota | Sordariomycetes | Sordariales | Chaetomiaceae | Humicola | grisea | 1 |
| 5 | Fungi | Ascomycota | Leotiomycetes | Helotiales | Helotiales Incertae sedis | Cadophora |  | 1 |
| 6 | Fungi | Ascomycota | Leotiomycetes | Thelebolales | Pseudeurotiaceae | Pseudogymnoascus | pannorum | 1 |
| 7 | Fungi | Basidiomycota | Agaricomycetes | Auriculariales | Exidiaceae |  |  | 1 |
| 8 | Fungi | Ascomycota | Sordariomycetes | Sordariales | Chaetomiaceae | Botryotrichum | spirotrichum | 1 |
| 9 | Fungi | Ascomycota | Dothideomycetes | Dothideales | Aureobasidiaceae | Aureobasidium | pullulans | 3 |
| 10 | Fungi | Ascomycota | Eurotiomycetes | Eurotiales | Aspergillaceae | Aspergillus |  | 2 |
| 11 | Fungi | Ascomycota | Sordariomycetes | Sordariales | Lasiosphaeriaceae | Podospora |  | 1 |
| 12 | Fungi | Ascomycota | Sordariomycetes | Microascales | Halosphaeriaceae |  |  | 2 |
| 13 | Fungi | Ascomycota | Sordariomycetes | Hypocreales | Nectriaceae | Nectria | ramulariae | 1 |
| 14 | Fungi | Ascomycota | Sordariomycetes |  |  |  |  | 1 |
| 15 | Fungi | Ascomycota | Sordariomycetes | Trichosphaeriales | Trichosphaeriaceae | Nigrospora | oryzae | 1 |
| 16 | Fungi | Ascomycota | Sordariomycetes | Hypocreales | Cordycipitaceae | Simplicillium | minatense | 1 |
| 17 | Fungi | Ascomycota | Sordariomycetes | Sordariales | Chaetomiaceae |  |  | 1 |
| 18 | Fungi | Ascomycota | Saccharomycetes | Saccharomycetales | Saccharomycetaceae | Saccharomyces |  | 2 |
| 19 | Fungi | Ascomycota | Sordariomycetes | Hypocreales | Nectriaceae | Dactylonectria | macrodidyma | 8 |
| 20 | Fungi | Glomeromycota | Glomeromycetes | Glomerales | Glomeraceae | Rhizophagus | irregularis | 4 |
| 21 | Fungi | Ascomycota | Pezizomycetes | Pezizales | Pyronemataceae | Pseudaleuria |  | 1 |
| 22 | Fungi | Ascomycota | Sordariomycetes | Microascales | Microascaceae |  |  | 1 |
| 23 | Fungi | Ascomycota | Eurotiomycetes | Eurotiales | Aspergillaceae | Aspergillus |  | 7 |
| 24 | Fungi | Ascomycota | Eurotiomycetes | Onygenales | Onygenales Incertae sedis | Chrysosporium | pseudomerdarium | 3 |

**Table S11.** Results of the permutational test of significance of the Procrustes analyses (using the function *protest* implemented in the R package *vegan* [[11](#_ENREF_11)]). The great concordance shown between the compared datasets (low M^2^ and high correlation coefficient) suggests that the filtering steps did not remarkably impact the composition of the original microbial profiles (Raw counts).

| Datasets | Procrustes Sum of Squares (M^2^) | Correlation in a symmetric Procrustes rotation | Significance |
| --- | --- | --- | --- |
| Raw counts Vs Removed low counts/singletones/Unclassified/Classified at Kingdom level only | 0.011 | 0.995 | 0.0001 |
| Raw counts Vs Removed low counts/singletones/Unclassified/Classified at Kingdom level only and contaminants | 0.015 | 0.993 | 0.0001 |

**Table S12.** Technical information related to the TaqMan® assay for *Malassezia restricta.*

| *Malassezia restricta* |  |
| --- | --- |
| Oligonucleotides | Forward *5’-GGCGGCCAAGCAGTGTT* |
|  | Reverse *5’-TTTAGGTGACATAGCAAATGACGTATC* |
|  | Probe 5’-FAM-*TTCTCCTGGCATGGCA*-MGB |
| Amplicon length | 62 bp |
| Limit of detection | 0.02 fg (22 molecules) gBlocks™ Gene Fragment |
| Calibration curve | Slope: -3.1838 |
|  | Y-intercept: 38.3  PCR efficiency: 106.15%  R^2^ 0.99  Linear dynamic range (# molecules): 0.02 fg (22)-2ng (2.14x10^9^) of a 426 bp gBlocks™ Gene Fragment |

**Table S13.** System information and R packages used in this study.

R version 4.0.2 (2020-06-22)

Platform: x86_64-apple-darwin17.0 (64-bit)

Running under: macOS Mojave 10.14

| R Package | Version |
| --- | --- |
| colorspace | 2.0-1 |
| viridisLite | 0.4.0 |
| microbiome | 1.12.0 |
| sfsmisc | 1.1-11 |
| DescTools | 0.99.41 |
| forcats | 0.5.1 |
| dplyr | 1.0.6 |
| readr | 1.4.0 |
| tibble | 3.1.2 |
| MASS | 7.3-54 |
| vegan | 2.5-7 |
| permute | 0.9-5 |
| IRanges | 2.24.1 |
| BiocGenerics | 0.36.1 |
| phyloseq | 1.36.0 |
| viridis | 0.6.1 |
| htmlTable | 2.2.1 |
| ANCOMBC | 1.0.5 |
| rstatix | 0.7.0 |
| stringr | 1.4.0 |
| purr | 0.3.4 |
| tidyr | 1.1.3 |
| tidyverse | 1.3.1 |
| mixOmics | 6.14.1 |
| decontam | 1.10.0 |
| lattice | 0.20-44 |
| DirichletMultinomial | 1.32.0 |
| S4Vectors | 0.28.1 |
| ggplot2 | 3.3.3 |
| ggeffects | 1.1.0 |
| car | 3.0-10 |

**References**

1. Rosenow, T.; Oudraad, M.C.; Murray, C.P.; Turkovic, L.; Kuo, W.; de Bruijne, M.; Ranganathan, S.C.; Tiddens, H.A.; Stick, S.M.; Australian Respiratory Early Surveillance Team for Cystic, F. PRAGMA-CF. A Quantitative Structural Lung Disease Computed Tomography Outcome in Young Children with Cystic Fibrosis. *American journal of respiratory and critical care medicine* **2015**, *191*, 1158-1165, doi:10.1164/rccm.201501-0061OC.

2. Jorth, P.; Ehsan, Z.; Rezayat, A.; Caldwell, E.; Pope, C.; Brewington, J.J.; Goss, C.H.; Benscoter, D.; Clancy, J.P.; Singh, P.K. Direct Lung Sampling Indicates That Established Pathogens Dominate Early Infections in Children with Cystic Fibrosis. *Cell reports* **2019**, *27*, 1190-1204 e1193, doi:10.1016/j.celrep.2019.03.086.

3. Buzina, W.; Braun, H.; Freudenschuss, K.; Lackner, A.; Habermann, W.; Stammberger, H. Fungal biodiversity -- as found in nasal mucus. *Medical Mycology* **2003**, *41*, 149-161, doi:10.1080/mmy.41.2.149.161.

4. Dupuy, A.K.; David, M.S.; Li, L.; Heider, T.N.; Peterson, J.D.; Montano, E.A.; Dongari-Bagtzoglou, A.; Diaz, P.I.; Strausbaugh, L.D. Redefining the human oral mycobiome with improved practices in amplicon-based taxonomy: discovery of Malassezia as a prominent commensal *PLoS One* **2014**, *9*, e90899, doi:10.1371/journal.pone.0090899.

5. Limon, J.J.; Skalski, J.H.; Underhill, D.M. Commensal Fungi in health and disease. *Cell host & microbe* **2017**, *22*, 156-165, doi:10.1016/j.chom.2017.07.002

6. Findley, K.; Oh, J.; Yang, J.; Conlan, S.; Deming, C.; Meyer, J.A.; Schoenfeld, D.; Nomicos, E.; Park, M.; Program, N.C.S., et al. Topographic diversity of fungal and bacterial communities in human skin. *Nature* **2013**, *498*, 367-370, doi:10.1038/nature12171

7. Willger, S.D.; Grim, S.L.; Dolben, E.L.; Shipunova, A.; Hampton, T.H.; Morrison, H.G.; Filkins, L.M.; O'Toole, G.A.; Moulton, L.A.; Ashare, A., et al. Characterization and quantification of the fungal microbiome in serial samples from individuals with cystic fibrosis. *Microbiome* **2014**, *2*, 40, doi:10.1186/2049-2618-2-40.

8. Soret, P.; Vandeborght, L.E.; Francis, F.; Coron, N.; Enaud, R.; Avalos, M.; Schaeverbeke, T.; Berger, P.; Fayon, M.; Thiebaut, R., et al. Respiratory mycobiome and suggestion of interkingdom network during acute pulmonary exacerbation in cystic fibrosis. *Scientific reports* **2020**, *10*, 3589, doi:10.1038/s41598-020-60015-4.

9. Rubio-Portillo, E.; Orts, D.; Llorca, E.; Fernandez, C.; Anton, J.; Ferrer, C.; Galvez, B.; Esteban, V.; Revelles, E.; Perez-Martin, C., et al. The domestic environment and the lung mycobiome. *Microorganisms* **2020**, *8*, 1717, doi:10.3390/microorganisms8111717.

10. Davis, N.M.; Proctor, D.M.; Holmes, S.P.; Relman, D.A.; Callahan, B.J. Simple statistical identification and removal of contaminant sequences in marker-gene and metagenomics data. *Microbiome* **2018**, *6*, 226, doi:10.1186/s40168-018-0605-2.

11. Jari Oksanen, F.G.B., Michael Friendly, Roeland Kindt, Pierre; Legendre, D.M., Peter R. Minchin, R. B. O'Hara, Gavin L. Simpson, Peter; Solymos, M.H.H.S., Eduard Szoecs and Helene Wagner. vegan: Community Ecology Package. R package version 2.4-0. Availabe online: https://CRAN.R-project.org/package=vegan (accessed on
